# Association of Bitter Metabolites and Flavonoid Synthesis Pathway in Jujube Fruit

**DOI:** 10.3389/fnut.2022.901756

**Published:** 2022-05-31

**Authors:** Qianqian Shi, Xi Li, Jiangtao Du, Yu Liu, Bingqi Shen, Xingang Li

**Affiliations:** ^1^College of Forestry, Northwest Agriculture and Forestry University, Xianyang, China; ^2^Key Comprehensive Laboratory of Forestry of Shaanxi Province, Northwest Agriculture and Forestry University, Xianyang, China

**Keywords:** nutrition, transcriptome, metabolome, *FLS* gene, jujube fruit

## Abstract

Jujube is rich in nutrients and can be eaten fresh or made into dried fruit, candied fruit, and preserved fruit. Its slightly bitter peel affects nutritional value and commercial value, but the mechanism of the formation of bitter substances is still unclear. We dynamically analyzed the biosynthesis of jujube peel bitterness and related nutrient metabolites through the transcriptome and metabolome. The results demonstrated that flavonoids were the main bitter substances in ‘Junzao’ jujube fruit skins and a total of 11,106 differentially expressed genes and 94 differentially abundant flavonoid metabolites were identified. Expression patterns of genes in the flavonoid synthesis pathway showed that *flavonol synthase* (*FLS*) expression was significantly correlated with quercetin content. Transient overexpression and virus induced gene silencing (VIGS) of *ZjFLS1* and *ZjFLS2* in jujube fruits and sour jujube seedlings significantly affected flavonol accumulation, especially the content of quercetin-3-O-rutinoside. Moreover, *in vitro* enzymatic reactions showed that *ZjFLS1* and *ZjFLS2* could catalyze the formation of quercetin from dihydroquercetin. These findings indicate that *ZjFLS* gene is the key gene in the biosynthesis of bitter substances in jujube fruit skins and provide basis for the research on the development of functional nutrients in jujube and the synthesis mechanism of bitter compounds.

## Introduction

Jujube (*Ziziphus jujuba* Mill.) is an economically important tree species in the family Rhamnoides and is widely planted worldwide because of its strong adaptability. It has more than 3,000 years of cultivation history, and more than 700 commercial cultivars are currently grown. Jujube fruit is nutritious, and dried jujube is also used as a Chinese medicine (1). The fruit contains a variety of functional substances that are beneficial to the human body, such as organic acids, phenols, flavonoids, saponins, and alkaloids with anti-tumor, anti-inflammatory, immunity-promoting, and calming effects (2, 3). Nonetheless, the bitter taste of jujube fruit skins has gradually attracted attention owing to the increased yield of jujube fruits and the development of jujube products. It has been reported that the bitterness of ‘Junzao’ fruit is caused by nitrogen oxides, flavonoids, saponins, and other compounds. Flavonoids, in particular, contribute significantly to the formation of fruit bitterness (4, 5).

Coffee and tea are also well-known bitter beverages, but their bitter components are different. The bitter substances in coffee beans are mainly caffeine and trigonelline; furaneol, piperazines, and trigonelline pyrolysis products are also produced during the roasting process (6). However, the bitter substances in tea are primarily tea polyphenols, flavonoids, rutin, anthocyanins, and theaflavins (7). Polyphenols and alkaloids are the main bitter substances in cocoa beans. Their polyphenols are mainly anthocyanins, procyanidins, and catechins, and their alkaloids are mainly caffeine and theobromine (8). Bitter substances in some vegetables are also related to flavonols. Bitter melon, which is famous for its bitter taste, has anti-inflammatory, anti-diabetic, and anti-cancer effects (9). The bitter components of the bitter gourd are mainly triterpene glycosides, which have beneficial antioxidant and immune regulation effects (10). A large number of flavonoid glycosides with quercetin and kaempferol as aglycone structures were detected in *Brassica oleracea* (11). Carrots do not typically have a bitter taste, but at times they may synthesize bitter compounds such as amino acids, phenols, terpenoids, and others, thereby spoiling their taste (12, 13). The *Citrus* genus contains many important fruits such as lemon, pomelo, grapefruit, sweet orange, and others. Limonin, a triterpene compound, is not only the main flavor compound in citrus fruits but also the primary source of bitterness in their processed products (14, 15). Bitterness contributes to the unique flavor and aroma of plant-derived foods, but bitter substances in fruits can also harm their taste and commercial value. Therefore, studying bitter substances in fruits is useful for revealing the underlying mechanisms of their formation and providing guidance for their development and utilization as functional ingredients.

‘Junzao’ is one of the most productive jujube cultivars and is favored by consumers because of its good quality. Jujube fruit skins have a slightly bitter taste in both the fresh and dried state (16). The bitterness of ‘Junzao’ fruit is masked by its high sugar content, which diverts attention from its bitter taste. However, the specific molecular mechanisms of their formation are still not clear. Here, we identified and verified key genes for the formation of bitterness in jujube skins using a combination of transcriptomics, metabolomics, prokaryotic expression, and *in vitro* enzymatic assays. Our findings provide a theoretical basis for the development of jujube fruit bitter components and the genetic improvement of jujube fruit quality.

## Materials and Methods

### Plant Materials

Fruits of *Z. jujuba* Mill. ‘Junzao’ were collected from the Jujube Experimental Station of Northwest Agriculture and Forestry University in Qingjian County, Shaanxi Province, China. Jujube fruit skins of different developmental stages were collected at 30, 90, and 110 days after pollination (DAP). At least 20 fruits were collected from each tree, and fruits from three trees were collected at each developmental stage as three biological replicate samples. In addition, we also collected stems, young leaves, buds, petals, and ovaries. The jujube fruit skins were immediately frozen in liquid nitrogen and stored in an ultra-low temperature freezer at −80°C. *Nicotiana benthamiana* was cultivated in a light incubator with a photoperiod of 16 h light (24°C) and 8 h darkness (18°C).

### Flavonoid Measurement

Powdered jujube fruit skin samples (0.5 g) were placed in 4 mL of 1% hydrochloric acid-methanol extraction solution for 20 min with ultrasonication, then moved to a 4°C refrigerator for 24 h of extraction in the dark. The extracts were centrifuged at 12,000 rpm for 15 min, and the supernatant was filtered through a 0.22-μm organic filter membrane for further analysis. The measurement of total flavonoids was performed using a previously published method (17). Absorbance was measured at 510 nm. Flavonoid content was calculated from a calibration curve, using rutin as the standard (75–1,000 mg/L) to obtain rutin equivalents (RE).

### Metabolomics Analysis

Jujube skin samples from different developmental stages (DAP30, DAP90, and DAP110) were sent to MetWare Biotechnology Co., Ltd., (Wuhan, China) for metabolomic analysis. The extraction of samples, detection of flavonoid metabolites, and quantitative analysis of metabolites were performed using previously reported methods (18, 19). In a nutshell, 100 mg jujube skin powder was added into 1.0 mL 70% methanol solution containing 0.1 mg/L lidocaine, and the solution was refrigerated at 4°C for overnight. The extract was filtered by 0.22 μm microporous membrane and analyzed by liquid chromatography electrospray ionization tandem mass spectrometry (LC-ESI-MS/MS) The metabolite quantitative monitoring mode is set as multireaction monitoring (MRM). Metabolites with variable importance in projection (VIP) values ≥ 1 and | log_2_(fold change)| values ≥ 1 were considered to be differentially abundant (20).

### Transcriptome and Differential Expression Analysis

Total RNA was extracted from jujube fruit skin samples (three biological replicates each from DAP30, DAP90, and DAP110) using a Tiangen RNAprep Pure Kit (Beijing, China). Total RNA that passed quality checks for library construction was sent to the Novogene Bioinformatics Technology Co., Ltd., (Beijing, China) for RNA-seq analysis on the Illumina HiSeq 4000 platform (Illumina, San Diego, CA, United States). Clean reads were mapped to the ‘Junzao’ reference genome using HISAT2 software with default parameters (21). BLAST was used to compare the assembled sequences to the NR, Swiss-Prot, KEGG, EuKaryotic Orthologous Groups (KOG), eggNOG, Gene Ontology (GO), and Pfam databases, and 29,116 genes were annotated. Gene expression was calculated as fragments per kilobase per million mapped reads (FPKM). Genes with an adjusted *p*-value < 0.05 and a | log_2_(fold change) | value ≥ 1 as determined by the DESeq R package were defined as DEGs. DEGs were assigned to different metabolic pathways by KEGG annotation to further characterize their potential functions.

### Weighted Gene Co-expression Network Analysis

The weighted gene co-expression network analysis (WGCNA) software package was used for WGCNA (22). A co-expression network was constructed based on correlations between each pair of genes. Each node represents a gene, and each edge represents the strength of the co-expression relationship. Genes with similar expression patterns were clustered into modules, and modules were characterized by functional enrichment analysis. Only genes with an FPKM value ≥ 1 were used in the analysis. The similarity threshold for controlling module fusion was 0.5, the minModuleSize parameter was set to 30, and the upper limit for the number of genes displayed in the Cytoscape and VisANT interaction network was set to the default value of 150.

### Quantitative Real-Time PCR Analysis

DNase-treated RNA from jujube skins of different developmental stages was reverse transcribed using the TaKaRa PrimeScript RT Reagent Kit (TaKaRa, Dalian, China). Primer 5.0 software was used to design specific primers for quantitative real-time PCR, and all primers are listed in Supplementary Table 1. The TaKaRa Fluorescence Quantitative PCR Kit with SYBR Premix Ex Taq II was used to detect gene expression levels on the LightCycler 96 fluorescence quantitative PCR instrument. *UBQ1* and *UBQ*2 were used as internal reference genes (23), and data were analyzed using the 2^–ΔΔCT^ method (24). The qRT–PCR reaction program was 95°C for 30 s followed by 40 cycles of 94°C for 5 s, 58°C for 30 s, and 72°C for 30 s. There were three biological replicates and three technical replicates of each sample type.

### Transient Expression in *Ziziphus jujube* Fruit and Sour Jujube Seedlings

To further verify the functions of *ZjFLS1* and *ZjFLS2*, the CDS sequences of *ZjFLS1* and *ZjFLS2* without termination codons were cloned into the PC2300-GFP vector with C-terminal green fluorescent protein (GFP). *Agrobacterium* containing PC2300-ZjFLS1-GFP and PC2300-ZjFLS2-GFP was used to overexpress these genes in jujube fruits and sour jujube seedlings; the PC2300-GFP empty vector was used as the control. For virus induced gene silencing (VIGS), the specific fragments of *ZjFLS1* and *ZjFLS2* were cloned into the pTRV2 vector using the primers listed in Supplementary Table 2, and all fusion vectors were transformed into *A. tumefaciens* strain GV3101 (25). A mixture of *Agrobacterium* cells (100 μL) containing pTRV2-*ZjFLS1* + TRV1 and pTRV2-*ZjFLS2* + TRV1 was injected into the flesh of jujube fruit at the white maturity stage. There were approximately 20 fruits per treatment. After injection, the fruits were placed in a light incubator. Meanwhile, the mixed bacterial solution was also vacuum-infiltrated into 4-week-old sour jujube seedlings for 8 min (approximately 15 seedlings per treatment), and the pTRV1 + pTRV2 empty vector was used as the control. Treated fruits and sour jujube seedlings were cultured for 10 days and then sampled for flavonol measurement.

### Prokaryotic Expression and *in vitro* Enzymatic Reactions

The full-length ORFs of *ZjFLS1* and *ZjFLS2* were inserted between the *Nde*I and *Xho*I restriction sites of the prokaryotic expression vector pET-28b (primers listed in Supplementary Table 3). The resulting fusion vector plasmids pET28b-ZjFLS1 and pET28b-ZjFLS2 were transferred into *E. coli* strain BL21 cells, and positive strains were verified by PCR. These recombinant cells and cells that contained the empty vector pET28b (control) were cultured at 37°C for 16 h in 50 mL LB liquid medium that contained 1 mM isopropyl β-D-1-thiogalactopyranoside (IPTG). After induction, cells were harvested in 200 μL of 50 mM phosphate-buffered saline (PBS, pH 7.5) and then disrupted using ultrasound. After centrifugation (12,000 rpm for 5 min), the supernatant containing the recombinant proteins was collected. The soluble protein expression of ZjFLS1 and ZjFLS2 was verified by sodium dodecyl sulfate polyacrylamide gel electrophoresis (SDS–PAGE). The purification and quantification of the recombinant proteins were carried out as reported previously (26).

The enzymatic activities of *ZjFLS1* and *ZjFLS2* were assessed in a 500 μL reaction solution that contained 20 mM Tris–HCl buffer (pH 7.5), 40 μM ferrous sulfate, 1 mM 2-ketoglutarate, 1 mg sodium ascorbic acid, 0.25 mg catalase, 50 μg purified protein, and 20 μM dihydroquercetin as a substrate. The reaction solution was incubated at 30°C for 30 min, and then an equal volume of saturated EDTA was added to terminate the reaction. The reaction mixture was centrifuged at 12,000 rpm for 10 min, and the supernatant was filtered through a 0.22 μm organic filter membrane and analyzed by HPLC.

### Statistical Analysis

All data were statistically analyzed by SPSS23.0 (SPSS, IBM Corporation, United States.). One-way analysis of variance (ANOVA) and multiple comparisons using Student’s *t*-test. The difference is significant at *p* < 0.05 level.

## Results

### Metabolomic Analysis of Flavonoids During Jujube Fruit Development

**FIGURE 1:**
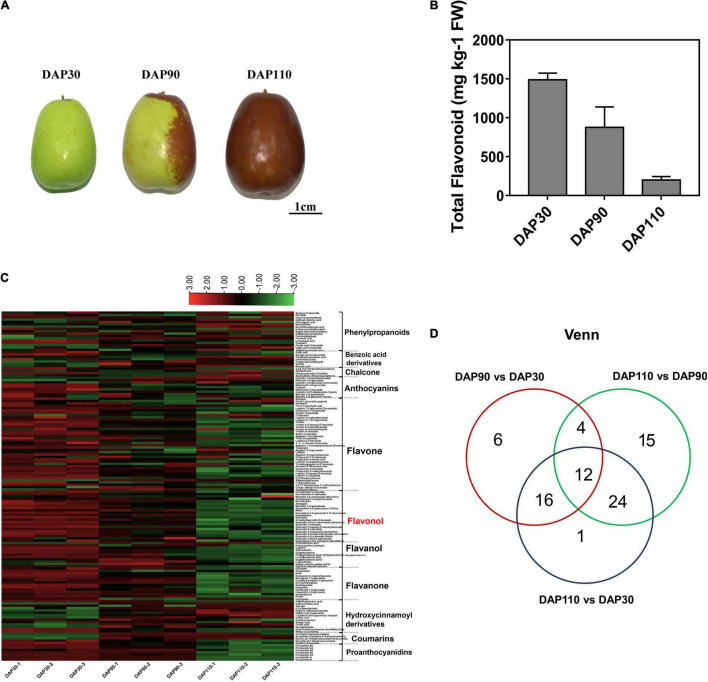
Flavonoid contents of jujube fruits at three developmental stages. **(A)** ‘Junzao’ jujube fruit at three developmental stages. **(B)** Total flavonoid content of jujube fruit skins at three developmental stages. **(C)** Heat map of differentially abundant metabolites in jujube fruit skins at three developmental stages. **(D)** Venn diagram of differentially abundant metabolites in jujube fruit skins at three developmental stages.

To identify the bitter compounds in the fruit skin of ‘Junzao’ jujube, total flavonoid content and individual flavonoid metabolites were measured in jujube fruit skins at different developmental stages (Figure 1A). The total flavonoid content of jujube skin gradually decreased during fruit development, from 1487.62 mg kg^–1^ FW at thirty days after pollination (DAP30) to 198.63 mg kg^–1^ FW at DAP110 (Figure 1B). Heat map and cluster analysis were performed on metabolites from all samples, and the three biological replicate samples from each developmental stage clustered together, indicating that samples from each stage showed good reproducibility (Figure 1C and Supplementary Figure 1). Metabolomic analysis identified 151 flavonoids, including 17 phenylpropanoids, 9 anthocyanins, 40 flavones, 28 flavonols, 9 flavanols, 14 flavanones, and 14 hydroxycinnamoyl derivatives. There were 38 differentially abundant flavonoid metabolites (DFMs) between DAP30 and DAP90, and most of these metabolites, particularly the flavonols, flavanols, and flavanones, decreased in abundance over time. There were 55 DFMs between DAP90 and DAP110. Interestingly, most flavonols, flavanols, and flavanones also decreased in abundance from DAP30 to DAP110. Among the downregulated metabolites, quercetin, quercetin O-hexoside, and quercetin 3-*O*-a-L-rhamnoside showed the greatest decreases, and their contents decreased by more than 10-fold (Figure 1C). A Venn diagram of DFMs from different developmental stages showed that 12 DFMs were common to all stage comparisons, and these included the three flavonol compounds above (Figure 1D). These results indicated that flavonoid contents decreased over the course of jujube fruit development, a finding that may be related to changes in bitterness during fruit skin development.

### Transcriptome Analysis Reveals Changes in Flavonoid Biological Processes During Jujube Development

**FIGURE 2:**
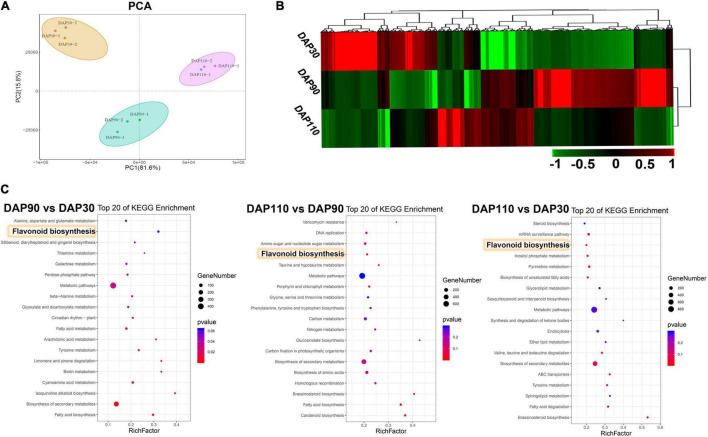
Transcriptome analysis during the development of ‘Junzao’ jujube fruit skins. **(A)** Principal component analysis of the transcriptome data. **(B)** Expression patterns of DEGs at three developmental stages of jujube fruit skins. **(C)** KEGG enrichment analysis of DEGs from three comparisons of jujube fruit skins at different developmental stages (DAP90 vs. DAP30, DAP110 vs. DAP90, and DAP110 vs. DAP30). Yellow rectangles highlight the flavonoid biosynthesis pathway.

RNA sequencing was performed on ‘Junzao’ fruit skins from three developmental stages (DAP30, DAP90, and DAP110; three biological replicates per stage) to explore the molecular mechanisms of bitter substance formation. Between 50,000,750 and 60,735,776 clean reads were obtained per sample, and 90.87–92.25% of the clean reads mapped to the jujube reference genome. Approximately 89.64% of the bases from clean reads of all nine libraries had quality scores of at least Q30 (0.02% error rate), indicating that the reads were of high quality. Their GC content was 43.50% (Supplementary Table 4). A principal component analysis (PCA) showed that the nine samples clustered into three groups by developmental stage, highlighting the reproducibility of the samples and the reliability of the data (Figure 2A). Differentially expressed genes (DEGs) were defined based on thresholds of | log_2_(fold-change) | > 1 and adjusted *p* < 0.01. A total of 13,286 DEGs were obtained (Figure 2B). The number of downregulated genes was greater than that of upregulated genes in the pairwise comparisons of DAP 90 vs. DAP30, DAP110 vs. DAP90, and DAP110 vs. DAP30 (Supplementary Figure 2 and Supplementary Table 5). KEGG and GO enrichment analysis of the DEGs showed that a large number of DEGs belonged to the flavonoid biosynthesis pathway and the secondary metabolite biosynthesis pathway (Figure 2C and Supplementary Figure 3). The expression levels of DEGs from flavonoid biosynthesis pathways were significantly downregulated over the course of fruit skin development, consistent with the observed decrease in flavonoid content. This interesting phenomenon implies that flavonoid content decreases during development and may be associated with changes in the bitterness of ‘Junzao’ fruit skins.

### Network Analysis for the Construction of Gene Co-expression Module*s*

**FIGURE 3:**
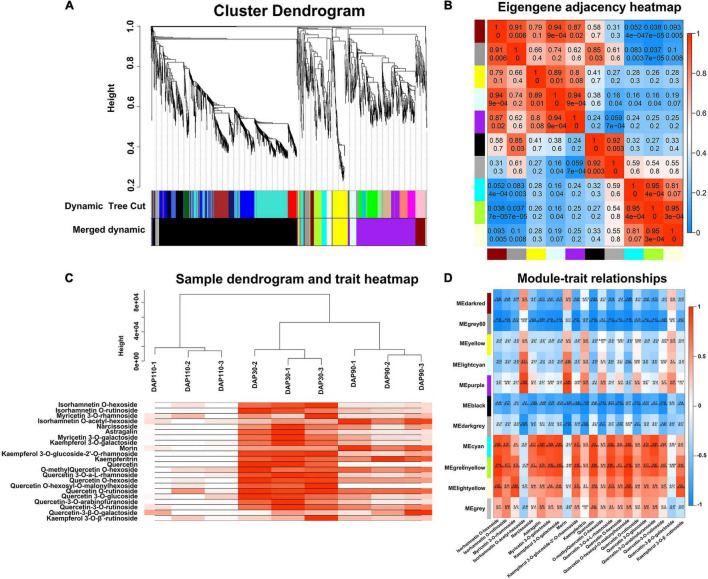
Weighted gene co-expression network analysis of differentially expressed genes. **(A)** Cluster dendrogram. **(B)** Eigengene adjacency heatmap. **(C)** Sample dendrogram and trait heatmap. **(D)** Heatmap of module and trait correlation.

We performed WGCNA of 5,279 non-redundant DEGs to investigate the gene regulation network of flavonoid biosynthesis during ‘Junzao’ skin development. The DEGs were clustered into 10 main branches, each one representing a module and marked with a different color. Each module contained highly related gene clusters, and the genes in a given module were co-expressed (Figure 3A). The genes in different modules showed different expression patterns, indicating that they may have different roles in flavonoid biosynthesis. Genes within a module had the same expression pattern, and the gene network was divided into different modules based on expression similarity to identify hub genes. The eigengene adjacency heatmap showed high correlations among the darkred, grey60, yellow, lightcyan, and purple modules, and the correlation between the black and darkgrey modules was >0.92. Moreover, the cyan, greenyellow, and lightyellow modules were also highly correlated (*R*^2^ > 0.81) (Figure 3B). To further illustrate the distribution of flavonols during jujube fruit development, 22 flavonols were used to analyze the correlation of modular traits, and a sample tree diagram and trait heat map were constructed. The fruit skin samples at DAP30 had the highest correlation with flavonols, followed by DAP90; by contrast, DAP110 samples had a low correlation with flavonols (Figure 3C). The cyan, greenyellow, and lightyellow modules contained 68, 77, and 43 genes, respectively, that were significantly positively correlated with flavonols, and their correlation coefficients (*R*^2^) with 22 flavonol compounds were generally >0.6 (Figure 3D). In general, the cyan, greenyellow, and lightyellow modules were highly correlated with flavonols and contained many genes involved in the flavonol biosynthesis pathway. These are potential candidate genes for the study of flavonol biosynthesis in jujube.

### The Expression Pattern of Flavonoid Synthesis-Related Genes During Jujube Fruit Development

**FIGURE 4:**
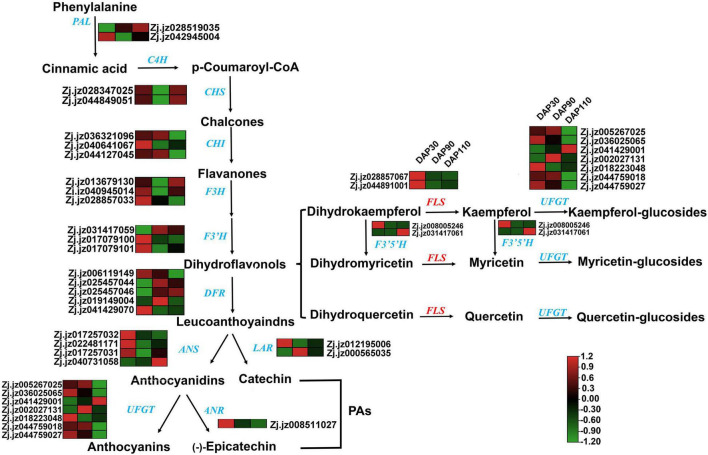
The flavonoid synthesis pathway and related gene expression patterns in jujube skins. Gene names are given in blue or red italics, and arrows represent enzymatic reactions.

Transcriptomic and metabolomic analysis showed that the flavonoid synthesis pathway contained a large number of DEGs and differentially abundant metabolites during jujube fruit skin development. Studies have shown that bitter components in plants are often related to flavonoids (16). Therefore, we explored the relationship between flavonoids and bitter substances in ‘Junzao’ jujube fruit skin development by analyzing the expression levels of genes involved in flavonoid synthesis, including *PAL*, *CHS*, *CHI*, *F3H*, *F3’H*, *LAR*, *ANR*, *FLS*, and *UFGT*. Most of these genes decreased in expression as the fruit matured, consistent with the trend in total flavonoid content (Figure 4). The accuracy of the transcriptome data was also verified by RT–qPCR, and the RT–qPCR results were consistent with the transcriptome sequencing results (Supplementary Figure 4).

**FIGURE 5:**
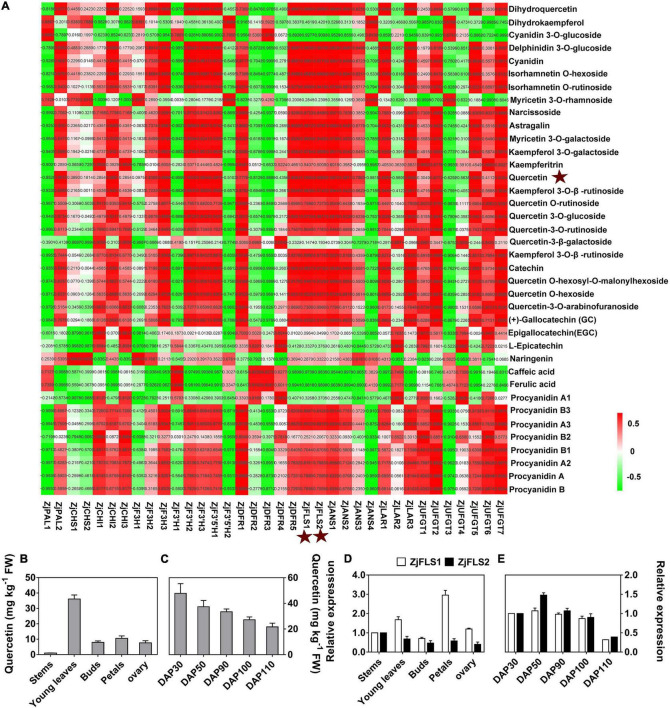
Correlation analysis of flavonoid metabolites and flavonoid-related genes. **(A)** Correlation analysis between flavonoid content and flavonoid-related gene expression. **(B)** Quercetin content in different tissues. **(C)** Quercetin content during jujube fruit skin development. **(D)** Relative expression levels of *ZjFLS1* and *ZjFLS2* in different tissues. **(E)** Relative expression levels of *ZjFLS1* and *ZjFLS2* during jujube fruit skin development.

To identify key genes for the synthesis of bitter substances during jujube skin development, we performed a correlation analysis of the expression levels of 36 flavonoid synthesis genes and the content of 38 flavonoids at different developmental stages (Figure 5A and Supplementary Table 6). The correlations between expression of the flavonoid synthase genes *ZjFLS1* (Zj.jz028857067) and *ZjFLS2* (Zj.jz044891001) and quercetin content had *R*^2^ values of 1.0000 and 0.9989, respectively, indicating that the *ZjFLS* genes may play an important role in flavonol synthesis and may act as switch genes to control bitter substance formation during jujube fruit skin development.

Next, we performed qRT–PCR and HPLC analysis to further verify the relationship between candidate gene expression (*ZjFLS1* and *ZjFLS2*) and quercetin (flavonol) content in different tissues and during fruit skin development. The quercetin content was significantly higher in young leaves than in other tissues, and it decreased gradually throughout the different stages of fruit skin development (Figures 5B,C). Moreover, the expression levels of *ZjFLS1* and *ZjFLS2* differed among different tissues, and the expression of *ZjFLS1* was significantly higher in young leaves and petals than in other tissues. The relative expression levels of *ZjFLS1* and *ZjFLS2* decreased continuously during fruit skin development, consistent with trends in quercetin content (Figures 5D,E).

### Functional Analysis of *ZjFLS1* and *ZjFLS2* by Transient Overexpression Assays and Virus Induced Gene Silencing in Jujube Fruit and Sour Jujube Seedlings

**FIGURE 6:**
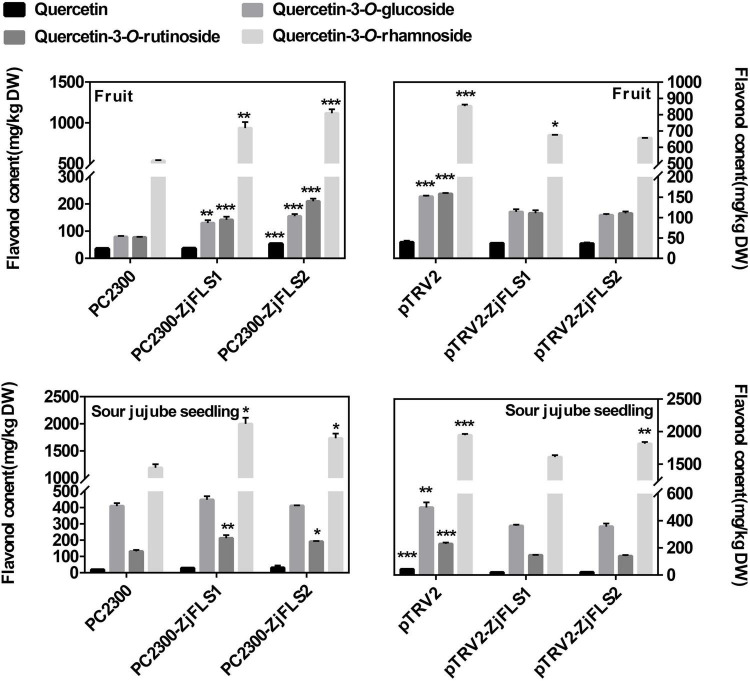
Flavonol contents of jujube fruit and sour jujube seeds 10 days after infiltration. Error bars show the means (*n* = 3). Data were analyzed with Student’s *t*-test (*P < 0.05, **P < 0.01, ***P < 0.001).

To further explore the functions of *ZjFLS1* and *ZjFLS2* in jujube flavonol biosynthesis, we transiently infected jujube fruits and sour jujube seedlings with the fusion vectors pC2300-*ZjFLS1*-GFP, pC2300-*ZjFLS2*-GFP, pTRV2-*ZjFLS1*, and pTRV2-*ZjFLS2*; pC2300 and pTRV1 + pTRV2 empty vectors served as controls. The metabolite content of the different treatments was measured by HPLC, and the results showed that transient overexpression of *ZjFLS1* and *ZjFLS2* significantly increased the content of quercetin-3-*O*-rutinoside in jujube fruits and jujube seedlings. In addition, VIGS experiments showed that transient silencing of *ZjFLS1* and *ZjFLS2* in jujube fruits and jujube seedlings significantly inhibited the synthesis of quercetin-3-*O*-glucoside and quercetin-3-*O*-rutinoside (Figure 6).

### *In vitro* Recombinant *ZjFLS1* and *ZjFLS2* Protein Activities

**FIGURE 7:**
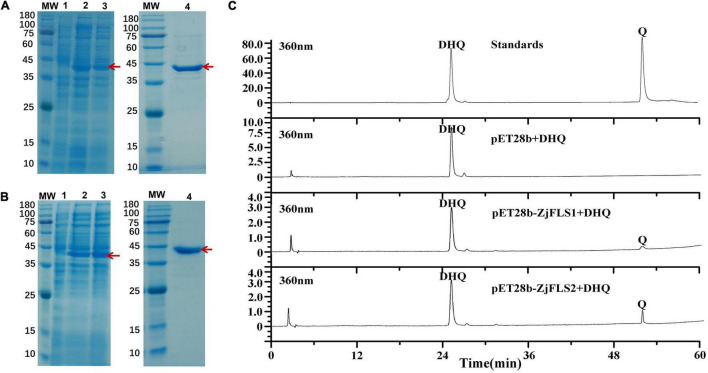
Prokaryotic expression and detection of enzymatic reaction products of ZjFLS1 and ZjFLS2 *in vitro*. **(A)** Analysis of ZjFLS1 recombinant protein by SDS–PAGE. **(B)** Analysis of ZjFLS2 recombinant protein by SDS–PAGE. MW, Molecular weight marker. Lane 1, non-induced recombinant bacteria (Control). Lanes 2–3, induced proteins of recombinant bacteria. Lane 4, the final protein sample. **(C)** Detection of enzymatic reaction products *in vitro*.

To verify the biochemical properties of *ZjFLS1* and *ZjFLS2*, the full-length ORFs of *ZjFLS1* and *ZjFLS2* were cloned into the pET-28b prokaryotic expression vector with a His-tag, and the vectors were individually transformed into *E. coli* BL21. *ZjFLS1* and *ZjFLS2* recombinant proteins were successfully induced with 1 mM IPTG at 37°C for 16 h. Most of the induced proteins were soluble and were purified by affinity chromatography (Figures 7A,B). We performed *in vitro* enzyme activity assays to measure the activity of the ZjFLS1 and ZjFLS2 recombinant proteins. Dihydroquercetin was used as the substrate in a reaction system with catalase, Fe^2+^, 2-oxoglutarate, ascorbate, and purified protein. The pET-28b empty vector protein was used as the control, and the reaction product was detected by HPLC. No new product was detected in the control group, but newly formed quercetin product was detected in the dihydroquercetin (DHQ) reaction system that contained *ZjFLS1* or *ZjFLS2* recombinant protein, indicating that the purified proteins could catalyze the formation of quercetin from dihydroquercetin (Figure 7C).

## Discussion

The flavors of fruits are mainly sweet and sour, but some fruits such as citrus, apples, and jujube also have bitter flavors. The bitter taste not only causes some fruits to taste bad but also reduces their commodity value. However, flavonoids, which are the main source of bitterness in fruits, are natural antioxidants that contribute to human health (27, 28), and studying their synthesis in fruits helps to reveal the mechanisms by which bitter substances are formed. Flavonoids polymerize to form odorless substances during fruit ripening, leading to a continuous decrease in bitterness and astringency; the content of bitter and astringent substances is therefore significantly lower in mature fruits than in immature fruits (29). The primary bitter substances in citrus are limonin and flavanone compounds (30). Flavonols are a class of secondary metabolites widely distributed in plants; some, such as quercetin and quercetin glycosides, have a bitter taste. Flavonols are also functional substances that protect plants from UV damage (31) and act as signaling substances to regulate the transport of growth hormones (32). They have antioxidant, anti-inflammatory, anti-cancer, and antibacterial effects in humans and animals and are widely used in clinical treatment (33–35).

Here, we used a combination of transcriptomics, metabolomics, prokaryotic expression, and *in vitro* enzyme assays to study the mechanism by which bitter substances are synthesized in jujube fruit skin. Based on the results, we speculate that the flavonoid pathway has an important function in the formation of bitterness in jujube fruit skin. A total of 11,106 DEGs and 94 differentially abundant flavonoid metabolites were identified in jujube fruit skins at different developmental stages. KEGG pathway enrichment analysis indicated that the DEGs were enriched in the secondary metabolite and flavonoid synthesis pathways. A large number of secondary metabolites were produced during fruit development, and their contents and the expression of their related genes changed over the course of development. The total flavonoid and quercetin contents of ‘Junzao’ jujube decreased as the fruit matured, which may be the main reason for the decrease in skin bitterness. A correlation analysis between the expression levels of flavonoid synthesis genes and the levels of individual flavonoid metabolites showed that *ZjFLS* expression was most highly correlated with quercetin content. Comprehensive analysis of the transcriptome and metabolome indicated that the contents of metabolites in the flavonoid synthesis pathway were highly correlated with the expression of related genes. However, correlations between flavonoid contents and gene expression patterns were low or even negative, suggesting that mRNA contents were not indicative of biosynthetic reaction rates. Biosynthetic reactions are also regulated by protein translation and by a variety of transcription factors, creating potential inconsistencies between transcriptome and metabolome data.

Study of the flavonoid synthesis pathway combined with intergroup correlation analysis of gene expression patterns and flavonoid contents revealed that *FLS* was a key gene for flavonol synthesis. Its encoded protein catalyzes hydroxylation at the C3 position of the flavonoid structure; it uses dihydroflavonol as a substrate to form quercetin, myricetin, and kaempferol (36). Furthermore, transient overexpression and silencing of *ZjFLS1* and *ZjFLS2* in jujube fruits and sour jujube seedlings significantly affected flavonol accumulation (Figure 6), especially that of quercetin-3-*O*-rutinoside (rutin), a bitter substance. These results further confirm that *ZjFLS1* and *ZjFLS2* are involved in the accumulation of flavonols (bitter substances) in jujube fruits. Intergroup correlation analysis also showed that *ZjF3’H2* was highly correlated with flavonol contents; *ZjF3’H2* produces dihydroflavonol substrates for flavonol synthesis and is therefore another key gene for flavonol synthesis.

We cloned and sequenced *FLS1* and *FLS2* and analyzed their phylogenetic relationships; they were highly homologous to FLS sequences from 23 other species. Plant FLS sequences are highly conserved, and *FLS* genes have been characterized in many higher plants, such as *Lactuca sativa* (37), tea (38), and *Oryza sativa* (39). SDS–PAGE showed that the molecular weights of ZjFLS1 and ZjFLS2 induced by IPTG were between 35 and 45 kDa, and soluble proteins were obtained for *in vitro* enzymatic assays. The ZjFLS1 and ZjFLS2 proteins catalyzed the formation of quercetin from dihydroquercetin, providing further evidence that ZjFLS is a key control gene for flavonol synthesis. Indeed, the ability of FLS recombinant protein to catalyze the formation of quercetin from dihydroquercetin has been documented in a number of species, confirming that it has dioxygenase activity in the flavonoid biosynthesis pathway (39, 40). Here, the successful cloning and functional identification of *ZjFLS* genes helps to resolve the pathway of flavonol biosynthesis in jujube.

## Conclusion

To investigate the molecular mechanisms of bitter substance formation during ripening and development of jujube fruit, we studied the bitter compounds in ‘Junzao’ jujube fruit skin using transcriptomics and metabolomics, and we identified the candidate flavonol-synthesis genes *ZjFLS1* and *ZjFLS2*. The key role of *ZjFLS* genes in jujube flavonol biosynthesis was confirmed by multiple molecular approaches such as clustering analysis, transient overexpression, transient silencing, prokaryotic expression, and *in vitro* enzyme activity assays. Our results support the notion that flavonoids are the main bitter substances in ‘Junzao’ jujube fruit skins and provide insight into the molecular mechanism of their formation. The research results will provide theoretical reference for the development of functional nutrients of jujube fruit and the improvement of jujube fruit quality.

## Data Availability Statement

The data presented in the study are deposited in the NCBI repository, accession number PRJNA830712 (https://www.ncbi.nlm.nih.gov/sra/PRJNA830712).

## Author Contributions

QS: conceptualization, methodology, software, investigation, writing – original draft, and review and editing. XL: validation and formal analysis. JD and YL: visualization and software. BS: software. XgL: resources, writing – review and editing, and supervision. All authors contributed to the article and approved the submitted version.

## Conflict of Interest

The authors declare that the research was conducted in the absence of any commercial or financial relationships that could be construed as a potential conflict of interest.

## Publisher’s Note

All claims expressed in this article are solely those of the authors and do not necessarily represent those of their affiliated organizations, or those of the publisher, the editors and the reviewers. Any product that may be evaluated in this article, or claim that may be made by its manufacturer, is not guaranteed or endorsed by the publisher.

## Supplementary Material

Supplementary Figure 1 Principal component analysis (PCA) of jujube skin metabolites at different development stages.

Supplementary Figure 2 Volcano plot of the genes in the fruit skins of the cultivars ‘Junzao’ in this comparison group of DAP90 vs DAP50, DAP110 vs. DAP50 and DAP110 vs DAP90.

Supplementary Figure 3 Enrichment analysis of GO term in differentially expressed genes. **(A)** DAP90 vs. DAP50, **(B)** DAP110 vs. DAP50, **(C)** DAP110 vs. DAP90.

Supplementary Figure 4 Relative expression levels of flavonoid synthesis pathway genes in jujube skins at different developmental stages. The R^2^ is the square of Pearson’s correlation coefficient.
