# Supplementary material for: Association of Bitter Metabolites and Flavonoid Synthesis Pathway in Jujube Fruit
Source: Front Nutr. 2022 May 31;9:901756. doi: 10.3389/fnut.2022.901756 (PMC9194943; doi:10.3389/fnut.2022.901756)
Supplement: Supplementary file 1 [file Table_1.DOCX]

**Table S1**

The primers related to flavonoid synthesis were analyzed by qRT-PCR.

| **Primer name** | **Primer Sequence(5'-3')** |
| --- | --- |
| PAL-F | CCCAATGGCGAATACCTCA |
| PAL-R | GACAAGATTTCCGACAGCACA |
| 4CL-F | GTGGGTCGGATCAAGGAGC |
| 4CL-R | TCGCCATACTTATCATCAGGGA |
| CHS-F | ATCACCGCCGTCACTTTCCG |
| CHS-R | CCGTCTATGGCTCCATCCGAGT |
| CHI-F | AATCCCTTTCCCTCCTCA |
| CHI-R | GCTCACTTCCCTGTTTACCT |
| F3H-F | TTGGGTTTCTATGACAGCG |
| F3H-R | TCTCAATTCGGGAGGGTAC |
| F3’H-F | GGAAGATCAGCTCCGTACACCT |
| F3’H-R | GACCTAGCATCACCCTCCCTAAA |
| LAR-F | AATGGCAGTGTCCTTGTTGTT |
| LAR-R | CTTGGAGTGCTTTAATGGTGG |
| ANR-F | GAAGGGCTATGCCGTCAAT |
| ANR-R | CCTCAGTTAGATCAGCACCAA |
| FLS1-F | CAAAAGAAACCATCCCAGAAGA |
| FLS1-R | ATGGACTCGAGATTCTACCTCCAT |
| FLS2-F | AACTCCAGCCCCGAAACCCTT |
| FLS2-R | TTCCAAGGCTTTCACCACTTCCAT |
| UBQ1-F | TGGATGATTCTGGCAAAG |
| UBQ1-R | GTAATGGCGGTCAAAGTG |
| UBQ2-F | CACCCGTTACTTGCTTTC |
| UBQ2-R | CTCTTCCCATTGTCCTCC |
