# Supplementary material for: Association of Bitter Metabolites and Flavonoid Synthesis Pathway in Jujube Fruit
Source: Front Nutr. 2022 May 31;9:901756. doi: 10.3389/fnut.2022.901756 (PMC9194943; doi:10.3389/fnut.2022.901756)
Supplement: Supplementary file 2 [file Table_2.DOCX]

**Table S2**

The Primers for transient silencing analysis.

| **Primer name** | **Primer Sequence(5'-3')** |
| --- | --- |
| pTRV2-ZjFLS1-SacI-F | TGTGAGTAAGGTTACCGAATTC TCTAGA GTATGCCAAGCCACCAGAT |
| pTRV2-ZjFLS1-XBaI-R | ATGCCCGGGCCTCGAGACGCGT GAGCTC CAAGCCCTACTCCCACCGA |
| pTRV2-ZjFLS2-SacI-F | TGTGAGTAAGGTTACCGAATTC TCTAGA TTCCATTGATCGACCTCTCGG |
| pTRV2-ZjFLS2-XBaI-R | ATGCCCGGGCCTCGAGACGCGT GAGCTC CTGCCCTCCTCACTTTCCTCT |
