# Supplementary material for: Association of Bitter Metabolites and Flavonoid Synthesis Pathway in Jujube Fruit
Source: Front Nutr. 2022 May 31;9:901756. doi: 10.3389/fnut.2022.901756 (PMC9194943; doi:10.3389/fnut.2022.901756)
Supplement: Supplementary file 3 [file Table_3.DOCX]

**Table S3**

The primers of prokaryotic expression analysis.

| **Primer name** | **Primer Sequence(5'-3')** |
| --- | --- |
| pET-28b-ZjFLS1-F | tgccgcgcggcagccatatgGAGGTAGAATCTCGAGTCCAT |
| pET-28b-ZjFLS1-R | tggtggtggtggtgctcgagTCACTGGGGAATCTTGTTGAGCT |
| pET-28b-ZjFLS2-F | tgccgcgcggcagccatatgGAAGTGGTGAAAGCCTTGGAA |
| pET-28b-ZjFLS2-R | tggtggtggtggtgctcgagCTATATCCTGAAGTGCTCAACTTGGATG |
