# Supplementary material for: Association of Bitter Metabolites and Flavonoid Synthesis Pathway in Jujube Fruit
Source: Front Nutr. 2022 May 31;9:901756. doi: 10.3389/fnut.2022.901756 (PMC9194943; doi:10.3389/fnut.2022.901756)
Supplement: Supplementary file 4 [file Table_4.DOCX]

**Table S4**

RNA-seq data quality.

| **Sample name** | **Clean reads** | **GC Content(%)** | **Q30 (%)** | **Error rate(%)** | **Total mapped(%)** |
| --- | --- | --- | --- | --- | --- |
| DAP30-1 | 56143744 | 43.26 | 90 | 0.02 | 51020207(90.87%) |
| DAP30-2 | 51944360 | 43.9 | 89.72 | 0.02 | 47434718 (91.32%) |
| DAP30-3 | 60735776 | 43.5 | 89.74 | 0.02 | 55368418 (91.16%) |
| DAP90-1 | 55964776 | 43.75 | 89.92 | 0.02 | 51245761 (91.57%) |
| DAP90-2 | 57931336 | 43.7 | 90.01 | 0.02 | 53102487 (91.66%) |
| DAP90-3 | 59368308 | 43.72 | 90.48 | 0.02 | 54337446 (91.53%) |
| DAP110-1 | 50000750 | 44.22 | 88.38 | 0.02 | 45720107 (91.44%) |
| DAP110-2 | 51772344 | 43.88 | 89.63 | 0.02 | 49132658(91.97%) |
| DAP110-3 | 56101474 | 43.68 | 88.89 | 0.02 | 51752606 (92.25%) |
