# Supplementary material for: Association of Bitter Metabolites and Flavonoid Synthesis Pathway in Jujube Fruit
Source: Front Nutr. 2022 May 31;9:901756. doi: 10.3389/fnut.2022.901756 (PMC9194943; doi:10.3389/fnut.2022.901756)
Supplement: Supplementary file 5 [file Table_5.DOCX]

**Table S5**

Correlation analysis of flavonoid metabolites and related genes.

| Compounds | Gene ID | gene name | cor | p_value |
| --- | --- | --- | --- | --- |
| Dihydroquercetin | Zj.jz028519035 | ZjPAL1 | -0.818572 | 0.389532 |
| Dihydrokaempferol | Zj.jz028519035 | ZjPAL1 | 0.8567319 | 0.3449823 |
| Cyanidin 3-O-glucoside | Zj.jz028519035 | ZjPAL1 | 0.9862396 | 0.1057327 |
| Delphinidin 3-O-glucoside | Zj.jz028519035 | ZjPAL1 | -0.789902 | 0.4202623 |
| Cyanidin | Zj.jz028519035 | ZjPAL1 | -0.928031 | 0.2430012 |
| Isorhamnetin O-hexoside | Zj.jz028519035 | ZjPAL1 | -0.82098 | 0.386855 |
| Isorhamnetin O-rutinoside | Zj.jz028519035 | ZjPAL1 | -0.968185 | 0.1610159 |
| Myricitrin | Zj.jz028519035 | ZjPAL1 | 0.7427983 | 0.4669962 |
| Narcissoside | Zj.jz028519035 | ZjPAL1 | -0.999206 | 0.0253689 |
| Astragalin | Zj.jz028519035 | ZjPAL1 | -0.925347 | 0.247548 |
| Myricetin 3-O-galactoside | Zj.jz028519035 | ZjPAL1 | -0.964558 | 0.1699981 |
| Kaempferol 3-O-galactoside | Zj.jz028519035 | ZjPAL1 | -0.940901 | 0.2199612 |
| Kaempferitrin | Zj.jz028519035 | ZjPAL1 | -0.900102 | 0.2869831 |
| Quercetin | Zj.jz028519035 | ZjPAL1 | -0.852754 | 0.3498614 |
| Kaempferol 3-O-β-rutinoside | Zj.jz028519035 | ZjPAL1 | -0.932955 | 0.2344412 |
| Quercetin O-rutinoside | Zj.jz028519035 | ZjPAL1 | -0.98707 | 0.1024852 |
| Quercetin 3-O-glucoside | Zj.jz028519035 | ZjPAL1 | -0.949932 | 0.2023034 |
| Quercetin 3-O-rutinoside | Zj.jz028519035 | ZjPAL1 | -0.996252 | 0.055135 |
| Quercetin-3-β-O-galactoside | Zj.jz028519035 | ZjPAL1 | -0.390114 | 0.744871 |
| Kaempferol 3-O-β-rutinoside | Zj.jz028519035 | ZjPAL1 | -0.995455 | 0.0607217 |
| Catechin | Zj.jz028519035 | ZjPAL1 | -0.934953 | 0.2308833 |
| Quercetin O-hexosyl-O-malonylhexoside | Zj.jz028519035 | ZjPAL1 | -0.974327 | 0.144565 |
| Quercetin O-hexoside | Zj.jz028519035 | ZjPAL1 | -0.971236 | 0.1530607 |
| Quercetin-3-O-arabinofuranoside | Zj.jz028519035 | ZjPAL1 | -0.979504 | 0.1291157 |
| (+)-Gallocatechin (GC) | Zj.jz028519035 | ZjPAL1 | -0.983951 | 0.1142097 |
| Epigallocatechin (EGC) | Zj.jz028519035 | ZjPAL1 | -0.601452 | 0.5891782 |
| L-Epicatechin | Zj.jz028519035 | ZjPAL1 | -0.208072 | 0.8665621 |
| Naringenin | Zj.jz028519035 | ZjPAL1 | 0.2539285 | 0.8365544 |
| Caffeic acid | Zj.jz028519035 | ZjPAL1 | 0.7126639 | 0.494977 |
| Ferulic acid | Zj.jz028519035 | ZjPAL1 | 0.7359372 | 0.4734837 |
| Procyanidin A1 | Zj.jz028519035 | ZjPAL1 | -0.214168 | 0.8625919 |
| Procyanidin B3 | Zj.jz028519035 | ZjPAL1 | -0.999648 | 0.0169038 |
| Procyanidin A3 | Zj.jz028519035 | ZjPAL1 | -0.99454 | 0.0665538 |
| Procyanidin B2 | Zj.jz028519035 | ZjPAL1 | -0.719824 | 0.4884454 |
| Procyanidin B1 | Zj.jz028519035 | ZjPAL1 | -0.971213 | 0.1531227 |
| Procyanidin A2 | Zj.jz028519035 | ZjPAL1 | -0.997729 | 0.0429174 |
| procyanidin A | Zj.jz028519035 | ZjPAL1 | -0.993635 | 0.0718682 |
| Procyanidin B | Zj.jz028519035 | ZjPAL1 | -0.993002 | 0.0753582 |
| Dihydroquercetin | Zj.jz042945004 | ZjPAL2 | 0.9770513 | 0.1366496 |
| Dihydrokaempferol | Zj.jz042945004 | ZjPAL2 | -0.200997 | 0.8711639 |
| Cyanidin 3-O-glucoside | Zj.jz042945004 | ZjPAL2 | -0.789723 | 0.4204489 |
| Delphinidin 3-O-glucoside | Zj.jz042945004 | ZjPAL2 | 0.9861911 | 0.1059193 |
| Cyanidin | Zj.jz042945004 | ZjPAL2 | 0.9026887 | 0.2831804 |
| Isorhamnetin O-hexoside | Zj.jz042945004 | ZjPAL2 | 0.976147 | 0.1393266 |
| Isorhamnetin O-rutinoside | Zj.jz042945004 | ZjPAL2 | 0.8399524 | 0.3651657 |
| Myricitrin | Zj.jz042945004 | ZjPAL2 | -0.010716 | 0.9931778 |
| Narcissoside | Zj.jz042945004 | ZjPAL2 | 0.7062036 | 0.5008127 |
| Astragalin | Zj.jz042945004 | ZjPAL2 | 0.9057388 | 0.2786336 |
| Myricetin 3-O-galactoside | Zj.jz042945004 | ZjPAL2 | 0.847525 | 0.3561835 |
| Kaempferol 3-O-galactoside | Zj.jz042945004 | ZjPAL2 | 0.8865282 | 0.3062204 |
| Kaempferitrin | Zj.jz042945004 | ZjPAL2 | 0.2892854 | 0.8131647 |
| Quercetin | Zj.jz042945004 | ZjPAL2 | 0.9618903 | 0.1763202 |
| Kaempferol 3-O-β-rutinoside | Zj.jz042945004 | ZjPAL2 | 0.8968215 | 0.2917404 |
| Quercetin O-rutinoside | Zj.jz042945004 | ZjPAL2 | 0.550772 | 0.6286668 |
| Quercetin 3-O-glucoside | Zj.jz042945004 | ZjPAL2 | 0.8733557 | 0.3238782 |
| Quercetin 3-O-rutinoside | Zj.jz042945004 | ZjPAL2 | 0.6112716 | 0.5813166 |
| Quercetin-3-β-O-galactoside | Zj.jz042945004 | ZjPAL2 | -0.413021 | 0.7289474 |
| Kaempferol 3-O-β-rutinoside | Zj.jz042945004 | ZjPAL2 | 0.7444119 | 0.4654599 |
| Catechin | Zj.jz042945004 | ZjPAL2 | 0.8943351 | 0.2952983 |
| Quercetin O-hexosyl-O-malonylhexoside | Zj.jz042945004 | ZjPAL2 | 0.8256506 | 0.3816165 |
| Quercetin O-hexoside | Zj.jz042945004 | ZjPAL2 | 0.8331059 | 0.3731209 |
| Quercetin-3-O-arabinofuranoside | Zj.jz042945004 | ZjPAL2 | 0.5153857 | 0.6552973 |
| (+)-Gallocatechin (GC) | Zj.jz042945004 | ZjPAL2 | 0.7978211 | 0.4119718 |
| Epigallocatechin (EGC) | Zj.jz042945004 | ZjPAL2 | -0.180217 | 0.8846402 |
| L-Epicatechin | Zj.jz042945004 | ZjPAL2 | -0.578526 | 0.6072563 |
| Naringenin | Zj.jz042945004 | ZjPAL2 | 0.5394505 | 0.637264 |
| Caffeic acid | Zj.jz042945004 | ZjPAL2 | -0.998799 | 0.0312046 |
| Ferulic acid | Zj.jz042945004 | ZjPAL2 | -0.996576 | 0.0526979 |
| Procyanidin A1 | Zj.jz042945004 | ZjPAL2 | -0.573428 | 0.6112265 |
| Procyanidin B3 | Zj.jz042945004 | ZjPAL2 | 0.696727 | 0.5092778 |
| Procyanidin A3 | Zj.jz042945004 | ZjPAL2 | 0.7504976 | 0.4596278 |
| Procyanidin B2 | Zj.jz042945004 | ZjPAL2 | -0.022974 | 0.985373 |
| Procyanidin B1 | Zj.jz042945004 | ZjPAL2 | 0.4827111 | 0.6793043 |
| Procyanidin A2 | Zj.jz042945004 | ZjPAL2 | 0.6263466 | 0.569099 |
| procyanidin A | Zj.jz042945004 | ZjPAL2 | 0.5902608 | 0.5980498 |
| Procyanidin B | Zj.jz042945004 | ZjPAL2 | 0.5858267 | 0.6015398 |
| Dihydroquercetin | Zj.jz028347025 | ZjCHS1 | 0.4455911 | 0.7059871 |
| Dihydrokaempferol | Zj.jz028347025 | ZjCHS1 | 0.6380307 | 0.5594986 |
| Cyanidin 3-O-glucoside | Zj.jz028347025 | ZjCHS1 | -0.016043 | 0.9897864 |
| Delphinidin 3-O-glucoside | Zj.jz028347025 | ZjCHS1 | 0.4882692 | 0.6752569 |
| Cyanidin | Zj.jz028347025 | ZjCHS1 | 0.2295977 | 0.8525179 |
| Isorhamnetin O-hexoside | Zj.jz028347025 | ZjCHS1 | 0.4418226 | 0.7086641 |
| Isorhamnetin O-rutinoside | Zj.jz028347025 | ZjCHS1 | 0.1027007 | 0.9345032 |
| Myricitrin | Zj.jz028347025 | ZjCHS1 | 0.7730258 | 0.4374846 |
| Narcissoside | Zj.jz028347025 | ZjCHS1 | -0.109969 | 0.9298498 |
| Astragalin | Zj.jz028347025 | ZjCHS1 | 0.236543 | 0.8479712 |
| Myricetin 3-O-galactoside | Zj.jz028347025 | ZjCHS1 | 0.1167246 | 0.925521 |
| Kaempferol 3-O-galactoside | Zj.jz028347025 | ZjCHS1 | 0.1942307 | 0.8755579 |
| Kaempferitrin | Zj.jz028347025 | ZjCHS1 | -0.56533 | 0.6174978 |
| Quercetin | Zj.jz028347025 | ZjCHS1 | 0.3889761 | 0.7456578 |
| Kaempferol 3-O-β-rutinoside | Zj.jz028347025 | ZjCHS1 | 0.2164905 | 0.8610779 |
| Quercetin O-rutinoside | Zj.jz028347025 | ZjCHS1 | -0.306034 | 0.8019957 |
| Quercetin 3-O-glucoside | Zj.jz028347025 | ZjCHS1 | 0.1669509 | 0.8932157 |
| Quercetin 3-O-rutinoside | Zj.jz028347025 | ZjCHS1 | -0.234444 | 0.8493458 |
| Quercetin-3-β-O-galactoside | Zj.jz028347025 | ZjCHS1 | -0.968735 | 0.1596098 |
| Kaempferol 3-O-β-rutinoside | Zj.jz028347025 | ZjCHS1 | -0.054632 | 0.9652026 |
| Catechin | Zj.jz028347025 | ZjCHS1 | 0.211031 | 0.8646358 |
| Quercetin O-hexosyl-O-malonylhexoside | Zj.jz028347025 | ZjCHS1 | 0.076965 | 0.9509541 |
| Quercetin O-hexoside | Zj.jz028347025 | ZjCHS1 | 0.0902631 | 0.9424584 |
| Quercetin-3-O-arabinofuranoside | Zj.jz028347025 | ZjCHS1 | -0.345579 | 0.7753651 |
| (+)-Gallocatechin (GC) | Zj.jz028347025 | ZjCHS1 | 0.0293549 | 0.9813094 |
| Epigallocatechin (EGC) | Zj.jz028347025 | ZjCHS1 | -0.879837 | 0.3153027 |
| L-Epicatechin | Zj.jz028347025 | ZjCHS1 | -0.998227 | 0.0379187 |
| Naringenin | Zj.jz028347025 | ZjCHS1 | 0.9943131 | 0.0679265 |
| Caffeic acid | Zj.jz028347025 | ZjCHS1 | -0.587096 | 0.6005422 |
| Ferulic acid | Zj.jz028347025 | ZjCHS1 | -0.559436 | 0.6220355 |
| Procyanidin A1 | Zj.jz028347025 | ZjCHS1 | -0.997836 | 0.0418889 |
| Procyanidin B3 | Zj.jz028347025 | ZjCHS1 | -0.123175 | 0.9213846 |
| Procyanidin A3 | Zj.jz028347025 | ZjCHS1 | -0.045483 | 0.9710347 |
| Procyanidin B2 | Zj.jz028347025 | ZjCHS1 | -0.793957 | 0.4160355 |
| Procyanidin B1 | Zj.jz028347025 | ZjCHS1 | -0.380712 | 0.7513582 |
| Procyanidin A2 | Zj.jz028347025 | ZjCHS1 | -0.215746 | 0.8615635 |
| procyanidin A | Zj.jz028347025 | ZjCHS1 | -0.259912 | 0.8326127 |
| Procyanidin B | Zj.jz028347025 | ZjCHS1 | -0.265202 | 0.8291226 |
| Dihydroquercetin | Zj.jz044849051 | ZjCHS2 | 0.2423229 | 0.8441814 |
| Dihydrokaempferol | Zj.jz044849051 | ZjCHS2 | 0.7888976 | 0.4213043 |
| Cyanidin 3-O-glucoside | Zj.jz044849051 | ZjCHS2 | 0.1996803 | 0.8720193 |
| Delphinidin 3-O-glucoside | Zj.jz044849051 | ZjCHS2 | 0.2888547 | 0.8134511 |
| Cyanidin | Zj.jz044849051 | ZjCHS2 | 0.0145887 | 0.9907122 |
| Isorhamnetin O-hexoside | Zj.jz044849051 | ZjCHS2 | 0.238241 | 0.8468583 |
| Isorhamnetin O-rutinoside | Zj.jz044849051 | ZjCHS2 | -0.113945 | 0.9273026 |
| Myricitrin | Zj.jz044849051 | ZjCHS2 | 0.891512 | 0.2992904 |
| Narcissoside | Zj.jz044849051 | ZjCHS2 | -0.321456 | 0.7916555 |
| Astragalin | Zj.jz044849051 | ZjCHS2 | 0.0217296 | 0.9861654 |
| Myricetin 3-O-galactoside | Zj.jz044849051 | ZjCHS2 | -0.099917 | 0.9362847 |
| Kaempferol 3-O-galactoside | Zj.jz044849051 | ZjCHS2 | -0.0216 | 0.9862478 |
| Kaempferitrin | Zj.jz044849051 | ZjCHS2 | -0.729717 | 0.4793035 |
| Quercetin | Zj.jz044849051 | ZjCHS2 | 0.1814344 | 0.883852 |
| Kaempferol 3-O-β-rutinoside | Zj.jz044849051 | ZjCHS2 | 0.0011432 | 0.9992722 |
| Quercetin O-rutinoside | Zj.jz044849051 | ZjCHS2 | -0.503893 | 0.6638014 |
| Quercetin 3-O-glucoside | Zj.jz044849051 | ZjCHS2 | -0.049319 | 0.96859 |
| Quercetin 3-O-rutinoside | Zj.jz044849051 | ZjCHS2 | -0.438314 | 0.7111516 |
| Quercetin-3-β-O-galactoside | Zj.jz044849051 | ZjCHS2 | -0.999434 | 0.0214156 |
| Kaempferol 3-O-β-rutinoside | Zj.jz044849051 | ZjCHS2 | -0.268403 | 0.8270083 |
| Catechin | Zj.jz044849051 | ZjCHS2 | -0.004445 | 0.99717 |
| Quercetin O-hexosyl-O-malonylhexoside | Zj.jz044849051 | ZjCHS2 | -0.139577 | 0.9108517 |
| Quercetin O-hexoside | Zj.jz044849051 | ZjCHS2 | -0.12635 | 0.9193473 |
| Quercetin-3-O-arabinofuranoside | Zj.jz044849051 | ZjCHS2 | -0.539574 | 0.6371709 |
| (+)-Gallocatechin (GC) | Zj.jz044849051 | ZjCHS2 | -0.186615 | 0.8804964 |
| Epigallocatechin (EGC) | Zj.jz044849051 | ZjCHS2 | -0.961551 | 0.1771085 |
| L-Epicatechin | Zj.jz044849051 | ZjCHS2 | -0.987621 | 0.1002755 |
| Naringenin | Zj.jz044849051 | ZjCHS2 | 0.9939147 | 0.0702678 |
| Caffeic acid | Zj.jz044849051 | ZjCHS2 | -0.398969 | 0.7387364 |
| Ferulic acid | Zj.jz044849051 | ZjCHS2 | -0.367789 | 0.7602297 |
| Procyanidin A1 | Zj.jz044849051 | ZjCHS2 | -0.98858 | 0.0963053 |
| Procyanidin B3 | Zj.jz044849051 | ZjCHS2 | -0.334019 | 0.7831904 |
| Procyanidin A3 | Zj.jz044849051 | ZjCHS2 | -0.259567 | 0.8328404 |
| Procyanidin B2 | Zj.jz044849051 | ZjCHS2 | -0.906266 | 0.2778412 |
| Procyanidin B1 | Zj.jz044849051 | ZjCHS2 | -0.570932 | 0.6131639 |
| Procyanidin A2 | Zj.jz044849051 | ZjCHS2 | -0.420985 | 0.7233692 |
| procyanidin A | Zj.jz044849051 | ZjCHS2 | -0.461785 | 0.6944184 |
| Procyanidin B | Zj.jz044849051 | ZjCHS2 | -0.466641 | 0.6909284 |
| Dihydroquercetin | Zj.jz036321096 | ZjCHI1 | 0.225186 | 0.855402 |
| Dihydrokaempferol | Zj.jz036321096 | ZjCHI1 | -0.982025 | 0.1208877 |
| Cyanidin 3-O-glucoside | Zj.jz036321096 | ZjCHI1 | -0.623276 | 0.5716027 |
| Delphinidin 3-O-glucoside | Zj.jz036321096 | ZjCHI1 | 0.1779108 | 0.8861323 |
| Cyanidin | Zj.jz036321096 | ZjCHI1 | 0.4415308 | 0.7088712 |
| Isorhamnetin O-hexoside | Zj.jz036321096 | ZjCHI1 | 0.229281 | 0.852725 |
| Isorhamnetin O-rutinoside | Zj.jz036321096 | ZjCHI1 | 0.5531046 | 0.626886 |
| Myricitrin | Zj.jz036321096 | ZjCHI1 | -0.999998 | 0.0011262 |
| Narcissoside | Zj.jz036321096 | ZjCHI1 | 0.7167706 | 0.4912389 |
| Astragalin | Zj.jz036321096 | ZjCHI1 | 0.4351114 | 0.713418 |
| Myricetin 3-O-galactoside | Zj.jz036321096 | ZjCHI1 | 0.5412954 | 0.6358681 |
| Kaempferol 3-O-galactoside | Zj.jz036321096 | ZjCHI1 | 0.4737069 | 0.6858312 |
| Kaempferitrin | Zj.jz036321096 | ZjCHI1 | 0.96078 | 0.1788869 |
| Quercetin | Zj.jz036321096 | ZjCHI1 | 0.2854237 | 0.8157314 |
| Kaempferol 3-O-β-rutinoside | Zj.jz036321096 | ZjCHI1 | 0.4535549 | 0.7003112 |
| Quercetin O-rutinoside | Zj.jz036321096 | ZjCHI1 | 0.8414671 | 0.3633848 |
| Quercetin 3-O-glucoside | Zj.jz036321096 | ZjCHI1 | 0.4979489 | 0.6681734 |
| Quercetin 3-O-rutinoside | Zj.jz036321096 | ZjCHI1 | 0.7989909 | 0.410735 |
| Quercetin-3-β-O-galactoside | Zj.jz036321096 | ZjCHI1 | 0.9054941 | 0.279001 |
| Kaempferol 3-O-β-rutinoside | Zj.jz036321096 | ZjCHI1 | 0.6769626 | 0.5265917 |
| Catechin | Zj.jz036321096 | ZjCHI1 | 0.4585285 | 0.6967534 |
| Quercetin O-hexosyl-O-malonylhexoside | Zj.jz036321096 | ZjCHI1 | 0.574446 | 0.6104351 |
| Quercetin O-hexoside | Zj.jz036321096 | ZjCHI1 | 0.5634718 | 0.6189307 |
| Quercetin-3-O-arabinofuranoside | Zj.jz036321096 | ZjCHI1 | 0.8633261 | 0.3367543 |
| (+)-Gallocatechin (GC) | Zj.jz036321096 | ZjCHI1 | 0.6128081 | 0.5800798 |
| Epigallocatechin (EGC) | Zj.jz036321096 | ZjCHI1 | 0.9813003 | 0.1233082 |
| L-Epicatechin | Zj.jz036321096 | ZjCHI1 | 0.8083775 | 0.4006921 |
| Naringenin | Zj.jz036321096 | ZjCHI1 | -0.835217 | 0.3706844 |
| Caffeic acid | Zj.jz036321096 | ZjCHI1 | -0.061463 | 0.960847 |
| Ferulic acid | Zj.jz036321096 | ZjCHI1 | -0.095119 | 0.9393537 |
| Procyanidin A1 | Zj.jz036321096 | ZjCHI1 | 0.8120329 | 0.3967219 |
| Procyanidin B3 | Zj.jz036321096 | ZjCHI1 | 0.7259791 | 0.4827738 |
| Procyanidin A3 | Zj.jz036321096 | ZjCHI1 | 0.6701916 | 0.5324238 |
| Procyanidin B2 | Zj.jz036321096 | ZjCHI1 | 0.9993713 | 0.0225754 |
| Procyanidin B1 | Zj.jz036321096 | ZjCHI1 | 0.8817381 | 0.3127473 |
| Procyanidin A2 | Zj.jz036321096 | ZjCHI1 | 0.7873039 | 0.4229526 |
| procyanidin A | Zj.jz036321096 | ZjCHI1 | 0.8145191 | 0.3940018 |
| Procyanidin B | Zj.jz036321096 | ZjCHI1 | 0.8176872 | 0.3905118 |
| Dihydroquercetin | Zj.jz040641067 | ZjCHI2 | 0.9945585 | 0.0664432 |
| Dihydrokaempferol | Zj.jz040641067 | ZjCHI2 | -0.307586 | 0.8009575 |
| Cyanidin 3-O-glucoside | Zj.jz040641067 | ZjCHI2 | -0.852441 | 0.3502425 |
| Delphinidin 3-O-glucoside | Zj.jz040641067 | ZjCHI2 | 0.9984269 | 0.0357129 |
| Cyanidin | Zj.jz040641067 | ZjCHI2 | 0.9445618 | 0.212974 |
| Isorhamnetin O-hexoside | Zj.jz040641067 | ZjCHI2 | 0.9941117 | 0.0691202 |
| Isorhamnetin O-rutinoside | Zj.jz040641067 | ZjCHI2 | 0.8945733 | 0.2949592 |
| Myricitrin | Zj.jz040641067 | ZjCHI2 | -0.120701 | 0.9229714 |
| Narcissoside | Zj.jz040641067 | ZjCHI2 | 0.7798346 | 0.4306063 |
| Astragalin | Zj.jz040641067 | ZjCHI2 | 0.9468827 | 0.2084272 |
| Myricetin 3-O-galactoside | Zj.jz040641067 | ZjCHI2 | 0.9007897 | 0.2859771 |
| Kaempferol 3-O-galactoside | Zj.jz040641067 | ZjCHI2 | 0.9320632 | 0.236014 |
| Kaempferitrin | Zj.jz040641067 | ZjCHI2 | 0.3928789 | 0.7429583 |
| Quercetin | Zj.jz040641067 | ZjCHI2 | 0.9861405 | 0.1061138 |
| Kaempferol 3-O-β-rutinoside | Zj.jz040641067 | ZjCHI2 | 0.9400618 | 0.221534 |
| Quercetin O-rutinoside | Zj.jz040641067 | ZjCHI2 | 0.6392856 | 0.5584604 |
| Quercetin 3-O-glucoside | Zj.jz040641067 | ZjCHI2 | 0.921657 | 0.2536718 |
| Quercetin 3-O-rutinoside | Zj.jz040641067 | ZjCHI2 | 0.6946594 | 0.5111102 |
| Quercetin-3-β-O-galactoside | Zj.jz040641067 | ZjCHI2 | -0.310281 | 0.7991538 |
| Kaempferol 3-O-β-rutinoside | Zj.jz040641067 | ZjCHI2 | 0.8133769 | 0.3952535 |
| Catechin | Zj.jz040641067 | ZjCHI2 | 0.9381414 | 0.2250918 |
| Quercetin O-hexosyl-O-malonylhexoside | Zj.jz040641067 | ZjCHI2 | 0.882727 | 0.3114101 |
| Quercetin O-hexoside | Zj.jz040641067 | ZjCHI2 | 0.8889188 | 0.3029145 |
| Quercetin-3-O-arabinofuranoside | Zj.jz040641067 | ZjCHI2 | 0.6065688 | 0.5850909 |
| (+)-Gallocatechin (GC) | Zj.jz040641067 | ZjCHI2 | 0.8593271 | 0.3417654 |
| Epigallocatechin (EGC) | Zj.jz040641067 | ZjCHI2 | -0.070867 | 0.9548467 |
| L-Epicatechin | Zj.jz040641067 | ZjCHI2 | -0.485242 | 0.6774627 |
| Naringenin | Zj.jz040641067 | ZjCHI2 | 0.443504 | 0.7074704 |
| Caffeic acid | Zj.jz040641067 | ZjCHI2 | -0.998124 | 0.0390018 |
| Ferulic acid | Zj.jz040641067 | ZjCHI2 | -0.999622 | 0.0175085 |
| Procyanidin A1 | Zj.jz040641067 | ZjCHI2 | -0.47978 | 0.6814329 |
| Procyanidin B3 | Zj.jz040641067 | ZjCHI2 | 0.7714422 | 0.4390714 |
| Procyanidin A3 | Zj.jz040641067 | ZjCHI2 | 0.818672 | 0.3894214 |
| Procyanidin B2 | Zj.jz040641067 | ZjCHI2 | 0.0871931 | 0.9444206 |
| Procyanidin B1 | Zj.jz040641067 | ZjCHI2 | 0.576164 | 0.6090979 |
| Procyanidin A2 | Zj.jz040641067 | ZjCHI2 | 0.7083358 | 0.4988926 |
| procyanidin A | Zj.jz040641067 | ZjCHI2 | 0.6755142 | 0.5278434 |
| Procyanidin B | Zj.jz040641067 | ZjCHI2 | 0.6714618 | 0.5313334 |
| Dihydroquercetin | Zj.jz044127045 | ZjCHI3 | 0.225186 | 0.855402 |
| Dihydrokaempferol | Zj.jz044127045 | ZjCHI3 | -0.982025 | 0.1208877 |
| Cyanidin 3-O-glucoside | Zj.jz044127045 | ZjCHI3 | -0.623276 | 0.5716027 |
| Delphinidin 3-O-glucoside | Zj.jz044127045 | ZjCHI3 | 0.1779108 | 0.8861323 |
| Cyanidin | Zj.jz044127045 | ZjCHI3 | 0.4415308 | 0.7088712 |
| Isorhamnetin O-hexoside | Zj.jz044127045 | ZjCHI3 | 0.229281 | 0.852725 |
| Isorhamnetin O-rutinoside | Zj.jz044127045 | ZjCHI3 | 0.5531046 | 0.626886 |
| Myricitrin | Zj.jz044127045 | ZjCHI3 | -0.999998 | 0.0011262 |
| Narcissoside | Zj.jz044127045 | ZjCHI3 | 0.7167706 | 0.4912389 |
| Astragalin | Zj.jz044127045 | ZjCHI3 | 0.4351114 | 0.713418 |
| Myricetin 3-O-galactoside | Zj.jz044127045 | ZjCHI3 | 0.5412954 | 0.6358681 |
| Kaempferol 3-O-galactoside | Zj.jz044127045 | ZjCHI3 | 0.4737069 | 0.6858312 |
| Kaempferitrin | Zj.jz044127045 | ZjCHI3 | 0.96078 | 0.1788869 |
| Quercetin | Zj.jz044127045 | ZjCHI3 | 0.2854237 | 0.8157314 |
| Kaempferol 3-O-β-rutinoside | Zj.jz044127045 | ZjCHI3 | 0.4535549 | 0.7003112 |
| Quercetin O-rutinoside | Zj.jz044127045 | ZjCHI3 | 0.8414671 | 0.3633848 |
| Quercetin 3-O-glucoside | Zj.jz044127045 | ZjCHI3 | 0.4979489 | 0.6681734 |
| Quercetin 3-O-rutinoside | Zj.jz044127045 | ZjCHI3 | 0.7989909 | 0.410735 |
| Quercetin-3-β-O-galactoside | Zj.jz044127045 | ZjCHI3 | 0.9054941 | 0.279001 |
| Kaempferol 3-O-β-rutinoside | Zj.jz044127045 | ZjCHI3 | 0.6769626 | 0.5265917 |
| Catechin | Zj.jz044127045 | ZjCHI3 | 0.4585285 | 0.6967534 |
| Quercetin O-hexosyl-O-malonylhexoside | Zj.jz044127045 | ZjCHI3 | 0.574446 | 0.6104351 |
| Quercetin O-hexoside | Zj.jz044127045 | ZjCHI3 | 0.5634718 | 0.6189307 |
| Quercetin-3-O-arabinofuranoside | Zj.jz044127045 | ZjCHI3 | 0.8633261 | 0.3367543 |
| (+)-Gallocatechin (GC) | Zj.jz044127045 | ZjCHI3 | 0.6128081 | 0.5800798 |
| Epigallocatechin (EGC) | Zj.jz044127045 | ZjCHI3 | 0.9813003 | 0.1233082 |
| L-Epicatechin | Zj.jz044127045 | ZjCHI3 | 0.8083775 | 0.4006921 |
| Naringenin | Zj.jz044127045 | ZjCHI3 | -0.835217 | 0.3706844 |
| Caffeic acid | Zj.jz044127045 | ZjCHI3 | -0.061463 | 0.960847 |
| Ferulic acid | Zj.jz044127045 | ZjCHI3 | -0.095119 | 0.9393537 |
| Procyanidin A1 | Zj.jz044127045 | ZjCHI3 | 0.8120329 | 0.3967219 |
| Procyanidin B3 | Zj.jz044127045 | ZjCHI3 | 0.7259791 | 0.4827738 |
| Procyanidin A3 | Zj.jz044127045 | ZjCHI3 | 0.6701916 | 0.5324238 |
| Procyanidin B2 | Zj.jz044127045 | ZjCHI3 | 0.9993713 | 0.0225754 |
| Procyanidin B1 | Zj.jz044127045 | ZjCHI3 | 0.8817381 | 0.3127473 |
| Procyanidin A2 | Zj.jz044127045 | ZjCHI3 | 0.7873039 | 0.4229526 |
| procyanidin A | Zj.jz044127045 | ZjCHI3 | 0.8145191 | 0.3940018 |
| Procyanidin B | Zj.jz044127045 | ZjCHI3 | 0.8176872 | 0.3905118 |
| Dihydroquercetin | Zj.jz013679130 | ZjF3H1 | 0.1592939 | 0.8981565 |
| Dihydrokaempferol | Zj.jz013679130 | ZjF3H1 | 0.8381033 | 0.3673292 |
| Cyanidin 3-O-glucoside | Zj.jz013679130 | ZjF3H1 | 0.28194 | 0.8180442 |
| Delphinidin 3-O-glucoside | Zj.jz013679130 | ZjF3H1 | 0.2067445 | 0.8674262 |
| Cyanidin | Zj.jz013679130 | ZjF3H1 | -0.070137 | 0.9553127 |
| Isorhamnetin O-hexoside | Zj.jz013679130 | ZjF3H1 | 0.1551412 | 0.9008335 |
| Isorhamnetin O-rutinoside | Zj.jz013679130 | ZjF3H1 | -0.197666 | 0.8733274 |
| Myricitrin | Zj.jz013679130 | ZjF3H1 | 0.9266706 | 0.2453152 |
| Narcissoside | Zj.jz013679130 | ZjF3H1 | -0.400489 | 0.7376804 |
| Astragalin | Zj.jz013679130 | ZjF3H1 | -0.063011 | 0.9598594 |
| Myricetin 3-O-galactoside | Zj.jz013679130 | ZjF3H1 | -0.183816 | 0.8823096 |
| Kaempferol 3-O-galactoside | Zj.jz013679130 | ZjF3H1 | -0.106185 | 0.9322727 |
| Kaempferitrin | Zj.jz013679130 | ZjF3H1 | -0.784997 | 0.4253284 |
| Quercetin | Zj.jz013679130 | ZjF3H1 | 0.0975057 | 0.9378271 |
| Kaempferol 3-O-β-rutinoside | Zj.jz013679130 | ZjF3H1 | -0.083543 | 0.9467527 |
| Quercetin O-rutinoside | Zj.jz013679130 | ZjF3H1 | -0.575228 | 0.6098263 |
| Quercetin 3-O-glucoside | Zj.jz013679130 | ZjF3H1 | -0.133721 | 0.9146149 |
| Quercetin 3-O-rutinoside | Zj.jz013679130 | ZjF3H1 | -0.512854 | 0.6571765 |
| Quercetin-3-β-O-galactoside | Zj.jz013679130 | ZjF3H1 | -0.998692 | 0.0325595 |
| Kaempferol 3-O-β-rutinoside | Zj.jz013679130 | ZjF3H1 | -0.349014 | 0.7730332 |
| Catechin | Zj.jz013679130 | ZjF3H1 | -0.089111 | 0.9431948 |
| Quercetin O-hexosyl-O-malonylhexoside | Zj.jz013679130 | ZjF3H1 | -0.222929 | 0.8568765 |
| Quercetin O-hexoside | Zj.jz013679130 | ZjF3H1 | -0.2099 | 0.8653722 |
| Quercetin-3-O-arabinofuranoside | Zj.jz013679130 | ZjF3H1 | -0.608933 | 0.5831957 |
| (+)-Gallocatechin (GC) | Zj.jz013679130 | ZjF3H1 | -0.26914 | 0.8265212 |
| Epigallocatechin (EGC) | Zj.jz013679130 | ZjF3H1 | -0.981353 | 0.1231333 |
| L-Epicatechin | Zj.jz013679130 | ZjF3H1 | -0.97079 | 0.1542506 |
| Naringenin | Zj.jz013679130 | ZjF3H1 | 0.9810166 | 0.1242429 |
| Caffeic acid | Zj.jz013679130 | ZjF3H1 | -0.319885 | 0.7927115 |
| Ferulic acid | Zj.jz013679130 | ZjF3H1 | -0.287721 | 0.8142048 |
| Procyanidin A1 | Zj.jz013679130 | ZjF3H1 | -0.972267 | 0.1502804 |
| Procyanidin B3 | Zj.jz013679130 | ZjF3H1 | -0.412638 | 0.7292152 |
| Procyanidin A3 | Zj.jz013679130 | ZjF3H1 | -0.340414 | 0.7788653 |
| Procyanidin B2 | Zj.jz013679130 | ZjF3H1 | -0.938806 | 0.2238661 |
| Procyanidin B1 | Zj.jz013679130 | ZjF3H1 | -0.638405 | 0.5591888 |
| Procyanidin A2 | Zj.jz013679130 | ZjF3H1 | -0.496285 | 0.6693941 |
| procyanidin A | Zj.jz013679130 | ZjF3H1 | -0.535239 | 0.6404433 |
| Procyanidin B | Zj.jz013679130 | ZjF3H1 | -0.539861 | 0.6369532 |
| Dihydroquercetin | Zj.jz040945014 | ZjF3H2 | 0.8685337 | 0.3301257 |
| Dihydrokaempferol | Zj.jz040945014 | ZjF3H2 | 0.1013619 | 0.93536 |
| Cyanidin 3-O-glucoside | Zj.jz040945014 | ZjF3H2 | -0.56995 | 0.613925 |
| Delphinidin 3-O-glucoside | Zj.jz040945014 | ZjF3H2 | 0.8914373 | 0.2993954 |
| Cyanidin | Zj.jz040945014 | ZjF3H2 | 0.7325538 | 0.4766565 |
| Isorhamnetin O-hexoside | Zj.jz040945014 | ZjF3H2 | 0.8664419 | 0.3328027 |
| Isorhamnetin O-rutinoside | Zj.jz040945014 | ZjF3H2 | 0.6390665 | 0.5586417 |
| Myricitrin | Zj.jz040945014 | ZjF3H2 | 0.2890126 | 0.8133461 |
| Narcissoside | Zj.jz040945014 | ZjF3H2 | 0.4619655 | 0.6942888 |
| Astragalin | Zj.jz040945014 | ZjF3H2 | 0.7373967 | 0.4721097 |
| Myricetin 3-O-galactoside | Zj.jz040945014 | ZjF3H2 | 0.6498546 | 0.5496596 |
| Kaempferol 3-O-galactoside | Zj.jz040945014 | ZjF3H2 | 0.7074439 | 0.4996965 |
| Kaempferitrin | Zj.jz040945014 | ZjF3H2 | -0.010431 | 0.9933592 |
| Quercetin | Zj.jz040945014 | ZjF3H2 | 0.835983 | 0.3697963 |
| Kaempferol 3-O-β-rutinoside | Zj.jz040945014 | ZjF3H2 | 0.723335 | 0.4852165 |
| Quercetin O-rutinoside | Zj.jz040945014 | ZjF3H2 | 0.2757572 | 0.8221429 |
| Quercetin 3-O-glucoside | Zj.jz040945014 | ZjF3H2 | 0.6875707 | 0.5173543 |
| Quercetin 3-O-rutinoside | Zj.jz040945014 | ZjF3H2 | 0.3464225 | 0.7747927 |
| Quercetin-3-β-O-galactoside | Zj.jz040945014 | ZjF3H2 | -0.666631 | 0.5354713 |
| Kaempferol 3-O-β-rutinoside | Zj.jz040945014 | ZjF3H2 | 0.5104793 | 0.658936 |
| Catechin | Zj.jz040945014 | ZjF3H2 | 0.7194648 | 0.4887743 |
| Quercetin O-hexosyl-O-malonylhexoside | Zj.jz040945014 | ZjF3H2 | 0.6189797 | 0.5750926 |
| Quercetin O-hexoside | Zj.jz040945014 | ZjF3H2 | 0.6294055 | 0.566597 |
| Quercetin-3-O-arabinofuranoside | Zj.jz040945014 | ZjF3H2 | 0.2353184 | 0.8487734 |
| (+)-Gallocatechin (GC) | Zj.jz040945014 | ZjF3H2 | 0.5808406 | 0.6054479 |
| Epigallocatechin (EGC) | Zj.jz040945014 | ZjF3H2 | -0.466313 | 0.6911642 |
| L-Epicatechin | Zj.jz040945014 | ZjF3H2 | -0.796105 | 0.4137802 |
| Naringenin | Zj.jz040945014 | ZjF3H2 | 0.7667069 | 0.4437879 |
| Caffeic acid | Zj.jz040945014 | ZjF3H2 | -0.938365 | 0.2246807 |
| Ferulic acid | Zj.jz040945014 | ZjF3H2 | -0.926163 | 0.246174 |
| Procyanidin A1 | Zj.jz040945014 | ZjF3H2 | -0.792316 | 0.4177504 |
| Procyanidin B3 | Zj.jz040945014 | ZjF3H2 | 0.4501319 | 0.7027539 |
| Procyanidin A3 | Zj.jz040945014 | ZjF3H2 | 0.5183353 | 0.6531039 |
| Procyanidin B2 | Zj.jz040945014 | ZjF3H2 | -0.321097 | 0.7918969 |
| Procyanidin B1 | Zj.jz040945014 | ZjF3H2 | 0.1985087 | 0.8727804 |
| Procyanidin A2 | Zj.jz040945014 | ZjF3H2 | 0.3643607 | 0.7625751 |
| procyanidin A | Zj.jz040945014 | ZjF3H2 | 0.3216489 | 0.7915259 |
| Procyanidin B | Zj.jz040945014 | ZjF3H2 | 0.3164532 | 0.7950159 |
| Dihydroquercetin | Zj.jz028857033 | ZjF3H3 | 0.9899016 | 0.0905497 |
| Dihydrokaempferol | Zj.jz028857033 | ZjF3H3 | -0.530558 | 0.6439646 |
| Cyanidin 3-O-glucoside | Zj.jz028857033 | ZjF3H3 | -0.95428 | 0.1932496 |
| Delphinidin 3-O-glucoside | Zj.jz028857033 | ZjF3H3 | 0.9819085 | 0.12128 |
| Cyanidin | Zj.jz028857033 | ZjF3H3 | 0.9961362 | 0.0559811 |
| Isorhamnetin O-hexoside | Zj.jz028857033 | ZjF3H3 | 0.9904889 | 0.0878727 |
| Isorhamnetin O-rutinoside | Zj.jz028857033 | ZjF3H3 | 0.9766086 | 0.1379663 |
| Myricitrin | Zj.jz028857033 | ZjF3H3 | -0.359377 | 0.7659785 |
| Narcissoside | Zj.jz028857033 | ZjF3H3 | 0.9090529 | 0.2736134 |
| Astragalin | Zj.jz028857033 | ZjF3H3 | 0.996738 | 0.0514343 |
| Myricetin 3-O-galactoside | Zj.jz028857033 | ZjF3H3 | 0.9795451 | 0.1289842 |
| Kaempferol 3-O-galactoside | Zj.jz028857033 | ZjF3H3 | 0.9923063 | 0.0790211 |
| Kaempferitrin | Zj.jz028857033 | ZjF3H3 | 0.6054762 | 0.5859654 |
| Quercetin | Zj.jz028857033 | ZjF3H3 | 0.996808 | 0.0508791 |
| Kaempferol 3-O-β-rutinoside | Zj.jz028857033 | ZjF3H3 | 0.9948654 | 0.0645411 |
| Quercetin O-rutinoside | Zj.jz028857033 | ZjF3H3 | 0.80766 | 0.4014675 |
| Quercetin 3-O-glucoside | Zj.jz028857033 | ZjF3H3 | 0.988491 | 0.0966789 |
| Quercetin 3-O-rutinoside | Zj.jz028857033 | ZjF3H3 | 0.8492431 | 0.3541173 |
| Quercetin-3-β-O-galactoside | Zj.jz028857033 | ZjF3H3 | -0.06883 | 0.9561467 |
| Kaempferol 3-O-β-rutinoside | Zj.jz028857033 | ZjF3H3 | 0.9307788 | 0.2382606 |
| Catechin | Zj.jz028857033 | ZjF3H3 | 0.9942842 | 0.0680989 |
| Quercetin O-hexosyl-O-malonylhexoside | Zj.jz028857033 | ZjF3H3 | 0.9707267 | 0.1544172 |
| Quercetin O-hexoside | Zj.jz028857033 | ZjF3H3 | 0.9738455 | 0.1459216 |
| Quercetin-3-O-arabinofuranoside | Zj.jz028857033 | ZjF3H3 | 0.7822949 | 0.428098 |
| (+)-Gallocatechin (GC) | Zj.jz028857033 | ZjF3H3 | 0.9581752 | 0.1847725 |
| Epigallocatechin (EGC) | Zj.jz028857033 | ZjF3H3 | 0.1747749 | 0.8881605 |
| L-Epicatechin | Zj.jz028857033 | ZjF3H3 | -0.257116 | 0.8344556 |
| Naringenin | Zj.jz028857033 | ZjF3H3 | 0.2112958 | 0.8644633 |
| Caffeic acid | Zj.jz028857033 | ZjF3H3 | -0.952982 | 0.1959947 |
| Ferulic acid | Zj.jz028857033 | ZjF3H3 | -0.962668 | 0.1745014 |
| Procyanidin A1 | Zj.jz028857033 | ZjF3H3 | -0.251084 | 0.8384258 |
| Procyanidin B3 | Zj.jz028857033 | ZjF3H3 | 0.9034321 | 0.2820785 |
| Procyanidin A3 | Zj.jz028857033 | ZjF3H3 | 0.9340889 | 0.2324285 |
| Procyanidin B2 | Zj.jz028857033 | ZjF3H3 | 0.3277375 | 0.7874277 |
| Procyanidin B1 | Zj.jz028857033 | ZjF3H3 | 0.7582544 | 0.452105 |
| Procyanidin A2 | Zj.jz028857033 | ZjF3H3 | 0.8592192 | 0.3418997 |
| procyanidin A | Zj.jz028857033 | ZjF3H3 | 0.8350731 | 0.3708505 |
| Procyanidin B | Zj.jz028857033 | ZjF3H3 | 0.8320447 | 0.3743405 |
| Dihydroquercetin | Zj.jz031417059 | ZjF3'H1 | -0.975498 | 0.1412182 |
| Dihydrokaempferol | Zj.jz031417059 | ZjF3'H1 | 0.1939617 | 0.8757325 |
| Cyanidin 3-O-glucoside | Zj.jz031417059 | ZjF3'H1 | 0.7852999 | 0.4250175 |
| Delphinidin 3-O-glucoside | Zj.jz031417059 | ZjF3'H1 | -0.984977 | 0.1104879 |
| Cyanidin | Zj.jz031417059 | ZjF3'H1 | -0.899578 | 0.287749 |
| Isorhamnetin O-hexoside | Zj.jz031417059 | ZjF3'H1 | -0.974564 | 0.1438951 |
| Isorhamnetin O-rutinoside | Zj.jz031417059 | ZjF3'H1 | -0.836036 | 0.3697342 |
| Myricitrin | Zj.jz031417059 | ZjF3'H1 | 0.0035399 | 0.9977464 |
| Narcissoside | Zj.jz031417059 | ZjF3'H1 | -0.701105 | 0.5053813 |
| Astragalin | Zj.jz031417059 | ZjF3'H1 | -0.902674 | 0.2832022 |
| Myricetin 3-O-galactoside | Zj.jz031417059 | ZjF3'H1 | -0.843694 | 0.3607521 |
| Kaempferol 3-O-galactoside | Zj.jz031417059 | ZjF3'H1 | -0.883185 | 0.310789 |
| Kaempferitrin | Zj.jz031417059 | ZjF3'H1 | -0.282408 | 0.8177333 |
| Quercetin | Zj.jz031417059 | ZjF3'H1 | -0.959903 | 0.1808888 |
| Kaempferol 3-O-β-rutinoside | Zj.jz031417059 | ZjF3'H1 | -0.893624 | 0.296309 |
| Quercetin O-rutinoside | Zj.jz031417059 | ZjF3'H1 | -0.544768 | 0.6332354 |
| Quercetin 3-O-glucoside | Zj.jz031417059 | ZjF3'H1 | -0.869838 | 0.3284468 |
| Quercetin 3-O-rutinoside | Zj.jz031417059 | ZjF3'H1 | -0.605576 | 0.5858852 |
| Quercetin-3-β-O-galactoside | Zj.jz031417059 | ZjF3'H1 | 0.4195457 | 0.7243788 |
| Kaempferol 3-O-β-rutinoside | Zj.jz031417059 | ZjF3'H1 | -0.739601 | 0.4700285 |
| Catechin | Zj.jz031417059 | ZjF3'H1 | -0.891101 | 0.2998668 |
| Quercetin O-hexosyl-O-malonylhexoside | Zj.jz031417059 | ZjF3'H1 | -0.821581 | 0.3861851 |
| Quercetin O-hexoside | Zj.jz031417059 | ZjF3'H1 | -0.829115 | 0.3776895 |
| Quercetin-3-O-arabinofuranoside | Zj.jz031417059 | ZjF3'H1 | -0.509223 | 0.6598659 |
| (+)-Gallocatechin (GC) | Zj.jz031417059 | ZjF3'H1 | -0.793474 | 0.4165404 |
| Epigallocatechin (EGC) | Zj.jz031417059 | ZjF3'H1 | 0.1872708 | 0.8800717 |
| L-Epicatechin | Zj.jz031417059 | ZjF3'H1 | 0.5843645 | 0.6026877 |
| Naringenin | Zj.jz031417059 | ZjF3'H1 | -0.545479 | 0.6326954 |
| Caffeic acid | Zj.jz031417059 | ZjF3'H1 | 0.9984216 | 0.0357732 |
| Ferulic acid | Zj.jz031417059 | ZjF3'H1 | 0.9959569 | 0.0572665 |
| Procyanidin A1 | Zj.jz031417059 | ZjF3'H1 | 0.5792924 | 0.6066579 |
| Procyanidin B3 | Zj.jz031417059 | ZjF3'H1 | -0.691561 | 0.5138464 |
| Procyanidin A3 | Zj.jz031417059 | ZjF3'H1 | -0.745736 | 0.4641964 |
| Procyanidin B2 | Zj.jz031417059 | ZjF3'H1 | 0.0301478 | 0.9808044 |
| Procyanidin B1 | Zj.jz031417059 | ZjF3'H1 | -0.476414 | 0.6838729 |
| Procyanidin A2 | Zj.jz031417059 | ZjF3'H1 | -0.620736 | 0.5736676 |
| procyanidin A | Zj.jz031417059 | ZjF3'H1 | -0.584453 | 0.6026184 |
| Procyanidin B | Zj.jz031417059 | ZjF3'H1 | -0.579996 | 0.6061084 |
| Dihydroquercetin | Zj.jz017079100 | ZjF3'H2 | 0.9982697 | 0.0374555 |
| Dihydrokaempferol | Zj.jz017079100 | ZjF3'H2 | -0.458102 | 0.6970588 |
| Cyanidin 3-O-glucoside | Zj.jz017079100 | ZjF3'H2 | -0.926062 | 0.2463438 |
| Delphinidin 3-O-glucoside | Zj.jz017079100 | ZjF3'H2 | 0.9942696 | 0.0681858 |
| Cyanidin | Zj.jz017079100 | ZjF3'H2 | 0.985358 | 0.1090753 |
| Isorhamnetin O-hexoside | Zj.jz017079100 | ZjF3'H2 | 0.9985082 | 0.0347785 |
| Isorhamnetin O-rutinoside | Zj.jz017079100 | ZjF3'H2 | 0.9553018 | 0.1910606 |
| Myricitrin | Zj.jz017079100 | ZjF3'H2 | -0.280389 | 0.8190728 |
| Narcissoside | Zj.jz017079100 | ZjF3'H2 | 0.8711823 | 0.3267076 |
| Astragalin | Zj.jz017079100 | ZjF3'H2 | 0.9865506 | 0.1045286 |
| Myricetin 3-O-galactoside | Zj.jz017079100 | ZjF3'H2 | 0.9593777 | 0.1820784 |
| Kaempferol 3-O-galactoside | Zj.jz017079100 | ZjF3'H2 | 0.9785436 | 0.1321153 |
| Kaempferitrin | Zj.jz017079100 | ZjF3'H2 | 0.5370734 | 0.6390596 |
| Quercetin | Zj.jz017079100 | ZjF3'H2 | 0.9999939 | 0.0022151 |
| Kaempferol 3-O-β-rutinoside | Zj.jz017079100 | ZjF3'H2 | 0.9829765 | 0.1176353 |
| Quercetin O-rutinoside | Zj.jz017079100 | ZjF3'H2 | 0.7557329 | 0.4545617 |
| Quercetin 3-O-glucoside | Zj.jz017079100 | ZjF3'H2 | 0.9724531 | 0.1497731 |
| Quercetin 3-O-rutinoside | Zj.jz017079100 | ZjF3'H2 | 0.8023069 | 0.4072115 |
| Quercetin-3-β-O-galactoside | Zj.jz017079100 | ZjF3'H2 | -0.151697 | 0.9030525 |
| Kaempferol 3-O-β-rutinoside | Zj.jz017079100 | ZjF3'H2 | 0.8970893 | 0.2913548 |
| Catechin | Zj.jz017079100 | ZjF3'H2 | 0.9819343 | 0.1211932 |
| Quercetin O-hexosyl-O-malonylhexoside | Zj.jz017079100 | ZjF3'H2 | 0.9473443 | 0.2075115 |
| Quercetin O-hexoside | Zj.jz017079100 | ZjF3'H2 | 0.9515331 | 0.1990158 |
| Quercetin-3-O-arabinofuranoside | Zj.jz017079100 | ZjF3'H2 | 0.7276853 | 0.4811923 |
| (+)-Gallocatechin (GC) | Zj.jz017079100 | ZjF3'H2 | 0.9310048 | 0.2378668 |
| Epigallocatechin (EGC) | Zj.jz017079100 | ZjF3'H2 | 0.092146 | 0.9412547 |
| L-Epicatechin | Zj.jz017079100 | ZjF3'H2 | -0.336725 | 0.7813614 |
| Naringenin | Zj.jz017079100 | ZjF3'H2 | 0.2919841 | 0.8113691 |
| Caffeic acid | Zj.jz017079100 | ZjF3'H2 | -0.974913 | 0.1429005 |
| Ferulic acid | Zj.jz017079100 | ZjF3'H2 | -0.981871 | 0.1214072 |
| Procyanidin A1 | Zj.jz017079100 | ZjF3'H2 | -0.330846 | 0.7853316 |
| Procyanidin B3 | Zj.jz017079100 | ZjF3'H2 | 0.8645771 | 0.3351727 |
| Procyanidin A3 | Zj.jz017079100 | ZjF3'H2 | 0.9010994 | 0.2855227 |
| Procyanidin B2 | Zj.jz017079100 | ZjF3'H2 | 0.2478957 | 0.8405219 |
| Procyanidin B1 | Zj.jz017079100 | ZjF3'H2 | 0.7013084 | 0.5051992 |
| Procyanidin A2 | Zj.jz017079100 | ZjF3'H2 | 0.813614 | 0.3949939 |
| procyanidin A | Zj.jz017079100 | ZjF3'H2 | 0.7863421 | 0.4239447 |
| Procyanidin B | Zj.jz017079100 | ZjF3'H2 | 0.7829435 | 0.4274348 |
| Dihydroquercetin | Zj.jz017079101 | ZjF3'H3 | 0.9988773 | 0.0301701 |
| Dihydrokaempferol | Zj.jz017079101 | ZjF3'H3 | -0.361273 | 0.7646845 |
| Cyanidin 3-O-glucoside | Zj.jz017079101 | ZjF3'H3 | -0.880831 | 0.3139695 |
| Delphinidin 3-O-glucoside | Zj.jz017079101 | ZjF3'H3 | 0.9999996 | 0.0005601 |
| Cyanidin | Zj.jz017079101 | ZjF3'H3 | 0.9617265 | 0.176701 |
| Isorhamnetin O-hexoside | Zj.jz017079101 | ZjF3'H3 | 0.9986692 | 0.0328471 |
| Isorhamnetin O-rutinoside | Zj.jz017079101 | ZjF3'H3 | 0.9185723 | 0.2586862 |
| Myricitrin | Zj.jz017079101 | ZjF3'H3 | -0.177036 | 0.8866984 |
| Narcissoside | Zj.jz017079101 | ZjF3'H3 | 0.814217 | 0.3943332 |
| Astragalin | Zj.jz017079101 | ZjF3'H3 | 0.963659 | 0.1721542 |
| Myricetin 3-O-galactoside | Zj.jz017079101 | ZjF3'H3 | 0.9240573 | 0.249704 |
| Kaempferol 3-O-galactoside | Zj.jz017079101 | ZjF3'H3 | 0.9511822 | 0.1997409 |
| Kaempferitrin | Zj.jz017079101 | ZjF3'H3 | 0.444609 | 0.7066853 |
| Quercetin | Zj.jz017079101 | ZjF3'H3 | 0.9939884 | 0.0698408 |
| Kaempferol 3-O-β-rutinoside | Zj.jz017079101 | ZjF3'H3 | 0.9579554 | 0.185261 |
| Quercetin O-rutinoside | Zj.jz017079101 | ZjF3'H3 | 0.6820384 | 0.5221873 |
| Quercetin 3-O-glucoside | Zj.jz017079101 | ZjF3'H3 | 0.942257 | 0.2173988 |
| Quercetin 3-O-rutinoside | Zj.jz017079101 | ZjF3'H3 | 0.7344961 | 0.4748372 |
| Quercetin-3-β-O-galactoside | Zj.jz017079101 | ZjF3'H3 | -0.255641 | 0.8354268 |
| Kaempferol 3-O-β-rutinoside | Zj.jz017079101 | ZjF3'H3 | 0.845185 | 0.3589805 |
| Catechin | Zj.jz017079101 | ZjF3'H3 | 0.9563369 | 0.1888188 |
| Quercetin O-hexosyl-O-malonylhexoside | Zj.jz017079101 | ZjF3'H3 | 0.908053 | 0.2751371 |
| Quercetin O-hexoside | Zj.jz017079101 | ZjF3'H3 | 0.9135616 | 0.2666414 |
| Quercetin-3-O-arabinofuranoside | Zj.jz017079101 | ZjF3'H3 | 0.6508589 | 0.5488179 |
| (+)-Gallocatechin (GC) | Zj.jz017079101 | ZjF3'H3 | 0.8870566 | 0.3054924 |
| Epigallocatechin (EGC) | Zj.jz017079101 | ZjF3'H3 | -0.013949 | 0.9911197 |
| L-Epicatechin | Zj.jz017079101 | ZjF3'H3 | -0.434662 | 0.7137357 |
| Naringenin | Zj.jz017079101 | ZjF3'H3 | 0.3917445 | 0.7437435 |
| Caffeic acid | Zj.jz017079101 | ZjF3'H3 | -0.993018 | 0.0752748 |
| Ferulic acid | Zj.jz017079101 | ZjF3'H3 | -0.996434 | 0.0537815 |
| Procyanidin A1 | Zj.jz017079101 | ZjF3'H3 | -0.429037 | 0.7177059 |
| Procyanidin B3 | Zj.jz017079101 | ZjF3'H3 | 0.8064255 | 0.4027984 |
| Procyanidin A3 | Zj.jz017079101 | ZjF3'H3 | 0.8500458 | 0.3531483 |
| Procyanidin B2 | Zj.jz017079101 | ZjF3'H3 | 0.1437814 | 0.9081476 |
| Procyanidin B1 | Zj.jz017079101 | ZjF3'H3 | 0.6217736 | 0.5728248 |
| Procyanidin A2 | Zj.jz017079101 | ZjF3'H3 | 0.7473836 | 0.4626195 |
| procyanidin A | Zj.jz017079101 | ZjF3'H3 | 0.7164075 | 0.4915704 |
| Procyanidin B | Zj.jz017079101 | ZjF3'H3 | 0.712572 | 0.4950604 |
| Dihydroquercetin | Zj.jz008005246 | ZjF3'5'H1 | 0.9999886 | 0.003038 |
| Dihydrokaempferol | Zj.jz008005246 | ZjF3'5'H1 | -0.400673 | 0.7375524 |
| Cyanidin 3-O-glucoside | Zj.jz008005246 | ZjF3'5'H1 | -0.900202 | 0.2868374 |
| Delphinidin 3-O-glucoside | Zj.jz008005246 | ZjF3'5'H1 | 0.9990541 | 0.0276922 |
| Cyanidin | Zj.jz008005246 | ZjF3'5'H1 | 0.9725278 | 0.1495689 |
| Isorhamnetin O-hexoside | Zj.jz008005246 | ZjF3'5'H1 | 0.9999597 | 0.005715 |
| Isorhamnetin O-rutinoside | Zj.jz008005246 | ZjF3'5'H1 | 0.9345783 | 0.2315541 |
| Myricitrin | Zj.jz008005246 | ZjF3'5'H1 | -0.218808 | 0.8595663 |
| Narcissoside | Zj.jz008005246 | ZjF3'5'H1 | 0.838213 | 0.3672011 |
| Astragalin | Zj.jz008005246 | ZjF3'5'H1 | 0.9741655 | 0.1450221 |
| Myricetin 3-O-galactoside | Zj.jz008005246 | ZjF3'5'H1 | 0.9395046 | 0.2225719 |
| Kaempferol 3-O-galactoside | Zj.jz008005246 | ZjF3'5'H1 | 0.963468 | 0.1726088 |
| Kaempferitrin | Zj.jz008005246 | ZjF3'5'H1 | 0.4823686 | 0.6795532 |
| Quercetin | Zj.jz008005246 | ZjF3'5'H1 | 0.9977505 | 0.0427087 |
| Kaempferol 3-O-β-rutinoside | Zj.jz008005246 | ZjF3'5'H1 | 0.9693099 | 0.1581289 |
| Quercetin O-rutinoside | Zj.jz008005246 | ZjF3'5'H1 | 0.7125776 | 0.4950552 |
| Quercetin 3-O-glucoside | Zj.jz008005246 | ZjF3'5'H1 | 0.9556698 | 0.1902667 |
| Quercetin 3-O-rutinoside | Zj.jz008005246 | ZjF3'5'H1 | 0.7627422 | 0.4477051 |
| Quercetin-3-β-O-galactoside | Zj.jz008005246 | ZjF3'5'H1 | -0.214219 | 0.8625589 |
| Kaempferol 3-O-β-rutinoside | Zj.jz008005246 | ZjF3'5'H1 | 0.8671893 | 0.3318484 |
| Catechin | Zj.jz008005246 | ZjF3'5'H1 | 0.9679209 | 0.1616867 |
| Quercetin O-hexosyl-O-malonylhexoside | Zj.jz008005246 | ZjF3'5'H1 | 0.9250742 | 0.248005 |
| Quercetin O-hexoside | Zj.jz008005246 | ZjF3'5'H1 | 0.9300599 | 0.2395094 |
| Quercetin-3-O-arabinofuranoside | Zj.jz008005246 | ZjF3'5'H1 | 0.6826144 | 0.5216858 |
| (+)-Gallocatechin (GC) | Zj.jz008005246 | ZjF3'5'H1 | 0.9059207 | 0.2783603 |
| Epigallocatechin (EGC) | Zj.jz008005246 | ZjF3'5'H1 | 0.0286659 | 0.9817482 |
| L-Epicatechin | Zj.jz008005246 | ZjF3'5'H1 | -0.395896 | 0.7408678 |
| Naringenin | Zj.jz008005246 | ZjF3'5'H1 | 0.352188 | 0.7708755 |
| Caffeic acid | Zj.jz008005246 | ZjF3'5'H1 | -0.98709 | 0.1024069 |
| Ferulic acid | Zj.jz008005246 | ZjF3'5'H1 | -0.991934 | 0.0809136 |
| Procyanidin A1 | Zj.jz008005246 | ZjF3'5'H1 | -0.390162 | 0.744838 |
| Procyanidin B3 | Zj.jz008005246 | ZjF3'5'H1 | 0.8308877 | 0.3756663 |
| Procyanidin A3 | Zj.jz008005246 | ZjF3'5'H1 | 0.8717149 | 0.3260163 |
| Procyanidin B2 | Zj.jz008005246 | ZjF3'5'H1 | 0.1858142 | 0.8810155 |
| Procyanidin B1 | Zj.jz008005246 | ZjF3'5'H1 | 0.6545779 | 0.5456928 |
| Procyanidin A2 | Zj.jz008005246 | ZjF3'5'H1 | 0.7750121 | 0.4354874 |
| procyanidin A | Zj.jz008005246 | ZjF3'5'H1 | 0.7454825 | 0.4644383 |
| Procyanidin B | Zj.jz008005246 | ZjF3'5'H1 | 0.7418173 | 0.4679283 |
| Dihydroquercetin | Zj.jz031417061 | ZjF3'5'H2 | -0.455729 | 0.6987571 |
| Dihydrokaempferol | Zj.jz031417061 | ZjF3'5'H2 | 0.998423 | 0.0357572 |
| Cyanidin 3-O-glucoside | Zj.jz031417061 | ZjF3'5'H2 | 0.7949846 | 0.4149578 |
| Delphinidin 3-O-glucoside | Zj.jz031417061 | ZjF3'5'H2 | -0.412248 | 0.7294874 |
| Cyanidin | Zj.jz031417061 | ZjF3'5'H2 | -0.646785 | 0.5522263 |
| Isorhamnetin O-hexoside | Zj.jz031417061 | ZjF3'5'H2 | -0.459468 | 0.6960801 |
| Isorhamnetin O-rutinoside | Zj.jz031417061 | ZjF3'5'H2 | -0.739376 | 0.470241 |
| Myricitrin | Zj.jz031417061 | ZjF3'5'H2 | 0.9694479 | 0.1577712 |
| Narcissoside | Zj.jz031417061 | ZjF3'5'H2 | -0.865034 | 0.334594 |
| Astragalin | Zj.jz031417061 | ZjF3'5'H2 | -0.641321 | 0.556773 |
| Myricetin 3-O-galactoside | Zj.jz031417061 | ZjF3'5'H2 | -0.729803 | 0.4792232 |
| Kaempferol 3-O-galactoside | Zj.jz031417061 | ZjF3'5'H2 | -0.673957 | 0.5291863 |
| Kaempferitrin | Zj.jz031417061 | ZjF3'5'H2 | -0.99939 | 0.022242 |
| Quercetin | Zj.jz031417061 | ZjF3'5'H2 | -0.510276 | 0.6590865 |
| Kaempferol 3-O-β-rutinoside | Zj.jz031417061 | ZjF3'5'H2 | -0.656981 | 0.5436663 |
| Quercetin O-rutinoside | Zj.jz031417061 | ZjF3'5'H2 | -0.947732 | 0.2067399 |
| Quercetin 3-O-glucoside | Zj.jz031417061 | ZjF3'5'H2 | -0.694187 | 0.5115285 |
| Quercetin 3-O-rutinoside | Zj.jz031417061 | ZjF3'5'H2 | -0.921402 | 0.2540901 |
| Quercetin-3-β-O-galactoside | Zj.jz031417061 | ZjF3'5'H2 | -0.774855 | 0.4356459 |
| Kaempferol 3-O-β-rutinoside | Zj.jz031417061 | ZjF3'5'H2 | -0.835853 | 0.3699468 |
| Catechin | Zj.jz031417061 | ZjF3'5'H2 | -0.661184 | 0.5401084 |
| Quercetin O-hexosyl-O-malonylhexoside | Zj.jz031417061 | ZjF3'5'H2 | -0.756526 | 0.4537901 |
| Quercetin O-hexoside | Zj.jz031417061 | ZjF3'5'H2 | -0.747732 | 0.4622858 |
| Quercetin-3-O-arabinofuranoside | Zj.jz031417061 | ZjF3'5'H2 | -0.960246 | 0.1801093 |
| (+)-Gallocatechin (GC) | Zj.jz031417061 | ZjF3'5'H2 | -0.786837 | 0.4234348 |
| Epigallocatechin (EGC) | Zj.jz031417061 | ZjF3'5'H2 | -0.904858 | 0.2799531 |
| L-Epicatechin | Zj.jz031417061 | ZjF3'5'H2 | -0.640641 | 0.557337 |
| Naringenin | Zj.jz031417061 | ZjF3'5'H2 | 0.6761094 | 0.5273293 |
| Caffeic acid | Zj.jz031417061 | ZjF3'5'H2 | 0.3027328 | 0.804202 |
| Ferulic acid | Zj.jz031417061 | ZjF3'5'H2 | 0.3347315 | 0.7827088 |
| Procyanidin A1 | Zj.jz031417061 | ZjF3'5'H2 | -0.645417 | 0.5533669 |
| Procyanidin B3 | Zj.jz031417061 | ZjF3'5'H2 | -0.871628 | 0.3261288 |
| Procyanidin A3 | Zj.jz031417061 | ZjF3'5'H2 | -0.830789 | 0.3757789 |
| Procyanidin B2 | Zj.jz031417061 | ZjF3'5'H2 | -0.960635 | 0.1792203 |
| Procyanidin B1 | Zj.jz031417061 | ZjF3'5'H2 | -0.970088 | 0.1561024 |
| Procyanidin A2 | Zj.jz031417061 | ZjF3'5'H2 | -0.913775 | 0.2663077 |
| procyanidin A | Zj.jz031417061 | ZjF3'5'H2 | -0.931297 | 0.2373569 |
| Procyanidin B | Zj.jz031417061 | ZjF3'5'H2 | -0.93328 | 0.2338668 |
| Dihydroquercetin | Zj.jz006119149 | ZjDFR1 | 0.7369473 | 0.4725332 |
| Dihydrokaempferol | Zj.jz006119149 | ZjDFR1 | -0.916514 | 0.2619812 |
| Cyanidin 3-O-glucoside | Zj.jz006119149 | ZjDFR1 | -0.956376 | 0.1887338 |
| Delphinidin 3-O-glucoside | Zj.jz006119149 | ZjDFR1 | 0.7034728 | 0.5032634 |
| Cyanidin | Zj.jz006119149 | ZjDFR1 | 0.8717256 | 0.3260023 |
| Isorhamnetin O-hexoside | Zj.jz006119149 | ZjDFR1 | 0.7397831 | 0.4698562 |
| Isorhamnetin O-rutinoside | Zj.jz006119149 | ZjDFR1 | 0.9274351 | 0.2440171 |
| Myricitrin | Zj.jz006119149 | ZjDFR1 | -0.823537 | 0.3839951 |
| Narcissoside | Zj.jz006119149 | ZjDFR1 | 0.9855463 | 0.1083701 |
| Astragalin | Zj.jz006119149 | ZjDFR1 | 0.8682039 | 0.3305491 |
| Myricetin 3-O-galactoside | Zj.jz006119149 | ZjDFR1 | 0.9220664 | 0.2529993 |
| Kaempferol 3-O-galactoside | Zj.jz006119149 | ZjDFR1 | 0.8888843 | 0.3029624 |
| Kaempferitrin | Zj.jz006119149 | ZjDFR1 | 0.9491051 | 0.203982 |
| Quercetin | Zj.jz006119149 | ZjDFR1 | 0.7776112 | 0.4328625 |
| Kaempferol 3-O-β-rutinoside | Zj.jz006119149 | ZjDFR1 | 0.8782351 | 0.3174423 |
| Quercetin O-rutinoside | Zj.jz006119149 | ZjDFR1 | 0.9995317 | 0.019484 |
| Quercetin 3-O-glucoside | Zj.jz006119149 | ZjDFR1 | 0.901248 | 0.2853045 |
| Quercetin 3-O-rutinoside | Zj.jz006119149 | ZjDFR1 | 0.9990422 | 0.0278661 |
| Quercetin-3-β-O-galactoside | Zj.jz006119149 | ZjDFR1 | 0.506511 | 0.6618699 |
| Kaempferol 3-O-β-rutinoside | Zj.jz006119149 | ZjDFR1 | 0.9746244 | 0.1437228 |
| Catechin | Zj.jz006119149 | ZjDFR1 | 0.880894 | 0.3138845 |
| Quercetin O-hexosyl-O-malonylhexoside | Zj.jz006119149 | ZjDFR1 | 0.9367885 | 0.2275662 |
| Quercetin O-hexoside | Zj.jz006119149 | ZjDFR1 | 0.9320359 | 0.2360619 |
| Quercetin-3-O-arabinofuranoside | Zj.jz006119149 | ZjDFR1 | 0.9973776 | 0.0461146 |
| (+)-Gallocatechin (GC) | Zj.jz006119149 | ZjDFR1 | 0.9524012 | 0.1972109 |
| Epigallocatechin (EGC) | Zj.jz006119149 | ZjDFR1 | 0.7002127 | 0.506177 |
| L-Epicatechin | Zj.jz006119149 | ZjDFR1 | 0.3334698 | 0.783561 |
| Naringenin | Zj.jz006119149 | ZjDFR1 | -0.377521 | 0.7535533 |
| Caffeic acid | Zj.jz006119149 | ZjDFR1 | -0.615413 | 0.5779781 |
| Ferulic acid | Zj.jz006119149 | ZjDFR1 | -0.641669 | 0.5564848 |
| Procyanidin A1 | Zj.jz006119149 | ZjDFR1 | 0.3393426 | 0.7795908 |
| Procyanidin B3 | Zj.jz006119149 | ZjDFR1 | 0.9877117 | 0.0999049 |
| Procyanidin A3 | Zj.jz006119149 | ZjDFR1 | 0.9725329 | 0.149555 |
| Procyanidin B2 | Zj.jz006119149 | ZjDFR1 | 0.8039608 | 0.4054443 |
| Procyanidin B1 | Zj.jz006119149 | ZjDFR1 | 0.99394 | 0.0701215 |
| Procyanidin A2 | Zj.jz006119149 | ZjDFR1 | 0.9980185 | 0.0400838 |
| procyanidin A | Zj.jz006119149 | ZjDFR1 | 0.9998471 | 0.0111329 |
| Procyanidin B | Zj.jz006119149 | ZjDFR1 | 0.9999279 | 0.0076429 |
| Dihydroquercetin | Zj.jz025457044 | ZjDFR2 | -0.84761 | 0.3560818 |
| Dihydrokaempferol | Zj.jz025457044 | ZjDFR2 | -0.141828 | 0.9094039 |
| Cyanidin 3-O-glucoside | Zj.jz025457044 | ZjDFR2 | 0.5359844 | 0.6398811 |
| Delphinidin 3-O-glucoside | Zj.jz025457044 | ZjDFR2 | -0.872226 | 0.3253515 |
| Cyanidin | Zj.jz025457044 | ZjDFR2 | -0.704199 | 0.5026126 |
| Isorhamnetin O-hexoside | Zj.jz025457044 | ZjDFR2 | -0.845371 | 0.3587588 |
| Isorhamnetin O-rutinoside | Zj.jz025457044 | ZjDFR2 | -0.607184 | 0.5845979 |
| Myricitrin | Zj.jz025457044 | ZjDFR2 | -0.327794 | 0.7873899 |
| Narcissoside | Zj.jz025457044 | ZjDFR2 | -0.425431 | 0.7202449 |
| Astragalin | Zj.jz025457044 | ZjDFR2 | -0.709252 | 0.4980659 |
| Myricetin 3-O-galactoside | Zj.jz025457044 | ZjDFR2 | -0.618334 | 0.5756157 |
| Kaempferol 3-O-galactoside | Zj.jz025457044 | ZjDFR2 | -0.678048 | 0.5256526 |
| Kaempferitrin | Zj.jz025457044 | ZjDFR2 | 0.0511807 | 0.9674031 |
| Quercetin | Zj.jz025457044 | ZjDFR2 | -0.812921 | 0.3957524 |
| Kaempferol 3-O-β-rutinoside | Zj.jz025457044 | ZjDFR2 | -0.694589 | 0.5111726 |
| Quercetin O-rutinoside | Zj.jz025457044 | ZjDFR2 | -0.236348 | 0.848099 |
| Quercetin 3-O-glucoside | Zj.jz025457044 | ZjDFR2 | -0.657402 | 0.5433104 |
| Quercetin 3-O-rutinoside | Zj.jz025457044 | ZjDFR2 | -0.307898 | 0.8007488 |
| Quercetin-3-β-O-galactoside | Zj.jz025457044 | ZjDFR2 | 0.6964595 | 0.5095152 |
| Kaempferol 3-O-β-rutinoside | Zj.jz025457044 | ZjDFR2 | -0.475006 | 0.6848921 |
| Catechin | Zj.jz025457044 | ZjDFR2 | -0.690558 | 0.5147305 |
| Quercetin O-hexosyl-O-malonylhexoside | Zj.jz025457044 | ZjDFR2 | -0.586452 | 0.6010488 |
| Quercetin O-hexoside | Zj.jz025457044 | ZjDFR2 | -0.597208 | 0.5925531 |
| Quercetin-3-O-arabinofuranoside | Zj.jz025457044 | ZjDFR2 | -0.195507 | 0.8747296 |
| (+)-Gallocatechin (GC) | Zj.jz025457044 | ZjDFR2 | -0.547178 | 0.6314041 |
| Epigallocatechin (EGC) | Zj.jz025457044 | ZjDFR2 | 0.501983 | 0.665208 |
| L-Epicatechin | Zj.jz025457044 | ZjDFR2 | 0.8201103 | 0.3878241 |
| Naringenin | Zj.jz025457044 | ZjDFR2 | -0.792238 | 0.4178318 |
| Caffeic acid | Zj.jz025457044 | ZjDFR2 | 0.9234963 | 0.2506368 |
| Ferulic acid | Zj.jz025457044 | ZjDFR2 | 0.9100212 | 0.2721301 |
| Procyanidin A1 | Zj.jz025457044 | ZjDFR2 | 0.8165259 | 0.3917943 |
| Procyanidin B3 | Zj.jz025457044 | ZjDFR2 | -0.41336 | 0.7287101 |
| Procyanidin A3 | Zj.jz025457044 | ZjDFR2 | -0.483047 | 0.67906 |
| Procyanidin B2 | Zj.jz025457044 | ZjDFR2 | 0.3594322 | 0.7659408 |
| Procyanidin B1 | Zj.jz025457044 | ZjDFR2 | -0.158394 | 0.8987365 |
| Procyanidin A2 | Zj.jz025457044 | ZjDFR2 | -0.326099 | 0.7885312 |
| procyanidin A | Zj.jz025457044 | ZjDFR2 | -0.282787 | 0.817482 |
| Procyanidin B | Zj.jz025457044 | ZjDFR2 | -0.277525 | 0.8209721 |
| Dihydroquercetin | Zj.jz025457046 | ZjDFR3 | -0.976525 | 0.1382138 |
| Dihydrokaempferol | Zj.jz025457046 | ZjDFR3 | 0.5924766 | 0.5963005 |
| Cyanidin 3-O-glucoside | Zj.jz025457046 | ZjDFR3 | 0.9739653 | 0.1455855 |
| Delphinidin 3-O-glucoside | Zj.jz025457046 | ZjDFR3 | -0.964994 | 0.1689441 |
| Cyanidin | Zj.jz025457046 | ZjDFR3 | -0.999915 | 0.008317 |
| Isorhamnetin O-hexoside | Zj.jz025457046 | ZjDFR3 | -0.977422 | 0.1355368 |
| Isorhamnetin O-rutinoside | Zj.jz025457046 | ZjDFR3 | -0.989957 | 0.0903023 |
| Myricitrin | Zj.jz025457046 | ZjDFR3 | 0.4281735 | 0.7183145 |
| Narcissoside | Zj.jz025457046 | ZjDFR3 | -0.937674 | 0.2259493 |
| Astragalin | Zj.jz025457046 | ZjDFR3 | -0.999982 | 0.0037703 |
| Myricetin 3-O-galactoside | Zj.jz025457046 | ZjDFR3 | -0.991853 | 0.0813201 |
| Kaempferol 3-O-galactoside | Zj.jz025457046 | ZjDFR3 | -0.998787 | 0.031357 |
| Kaempferitrin | Zj.jz025457046 | ZjDFR3 | -0.663311 | 0.5383013 |
| Quercetin | Zj.jz025457046 | ZjDFR3 | -0.988044 | 0.0985432 |
| Kaempferol 3-O-β-rutinoside | Zj.jz025457046 | ZjDFR3 | -0.999649 | 0.016877 |
| Quercetin O-rutinoside | Zj.jz025457046 | ZjDFR3 | -0.849503 | 0.3538034 |
| Quercetin 3-O-glucoside | Zj.jz025457046 | ZjDFR3 | -0.997038 | 0.0490148 |
| Quercetin 3-O-rutinoside | Zj.jz025457046 | ZjDFR3 | -0.886359 | 0.3064532 |
| Quercetin-3-β-O-galactoside | Zj.jz025457046 | ZjDFR3 | -0.005986 | 0.9961892 |
| Kaempferol 3-O-β-rutinoside | Zj.jz025457046 | ZjDFR3 | -0.955517 | 0.1905965 |
| Catechin | Zj.jz025457046 | ZjDFR3 | -0.999485 | 0.0204349 |
| Quercetin O-hexosyl-O-malonylhexoside | Zj.jz025457046 | ZjDFR3 | -0.985973 | 0.1067532 |
| Quercetin O-hexoside | Zj.jz025457046 | ZjDFR3 | -0.988113 | 0.0982575 |
| Quercetin-3-O-arabinofuranoside | Zj.jz025457046 | ZjDFR3 | -0.826697 | 0.3804339 |
| (+)-Gallocatechin (GC) | Zj.jz025457046 | ZjDFR3 | -0.976898 | 0.1371085 |
| Epigallocatechin (EGC) | Zj.jz025457046 | ZjDFR3 | -0.247935 | 0.8404964 |
| L-Epicatechin | Zj.jz025457046 | ZjDFR3 | 0.1841097 | 0.8821197 |
| Naringenin | Zj.jz025457046 | ZjDFR3 | -0.137592 | 0.9121274 |
| Caffeic acid | Zj.jz025457046 | ZjDFR3 | 0.9276455 | 0.2436588 |
| Ferulic acid | Zj.jz025457046 | ZjDFR3 | 0.9397231 | 0.2221655 |
| Procyanidin A1 | Zj.jz025457046 | ZjDFR3 | 0.1779764 | 0.8860899 |
| Procyanidin B3 | Zj.jz025457046 | ZjDFR3 | -0.93297 | 0.2344144 |
| Procyanidin A3 | Zj.jz025457046 | ZjDFR3 | -0.958179 | 0.1847644 |
| Procyanidin B2 | Zj.jz025457046 | ZjDFR3 | -0.397489 | 0.7397636 |
| Procyanidin B1 | Zj.jz025457046 | ZjDFR3 | -0.804897 | 0.4044409 |
| Procyanidin A2 | Zj.jz025457046 | ZjDFR3 | -0.895081 | 0.2942356 |
| procyanidin A | Zj.jz025457046 | ZjDFR3 | -0.873884 | 0.3231864 |
| Procyanidin B | Zj.jz025457046 | ZjDFR3 | -0.871206 | 0.3266765 |
| Dihydroquercetin | Zj.jz019149004 | ZjDFR4 | -0.490793 | 0.6734145 |
| Dihydrokaempferol | Zj.jz019149004 | ZjDFR4 | -0.597815 | 0.5920711 |
| Cyanidin 3-O-glucoside | Zj.jz019149004 | ZjDFR4 | 0.0671577 | 0.9572139 |
| Delphinidin 3-O-glucoside | Zj.jz019149004 | ZjDFR4 | -0.532262 | 0.6426843 |
| Cyanidin | Zj.jz019149004 | ZjDFR4 | -0.279073 | 0.8199454 |
| Isorhamnetin O-hexoside | Zj.jz019149004 | ZjDFR4 | -0.487125 | 0.6760915 |
| Isorhamnetin O-rutinoside | Zj.jz019149004 | ZjDFR4 | -0.153438 | 0.9019306 |
| Myricitrin | Zj.jz019149004 | ZjDFR4 | -0.739571 | 0.4700572 |
| Narcissoside | Zj.jz019149004 | ZjDFR4 | 0.0589926 | 0.9624224 |
| Astragalin | Zj.jz019149004 | ZjDFR4 | -0.285925 | 0.8153986 |
| Myricetin 3-O-galactoside | Zj.jz019149004 | ZjDFR4 | -0.167365 | 0.8929484 |
| Kaempferol 3-O-galactoside | Zj.jz019149004 | ZjDFR4 | -0.244145 | 0.8429853 |
| Kaempferitrin | Zj.jz019149004 | ZjDFR4 | 0.5224044 | 0.6500703 |
| Quercetin | Zj.jz019149004 | ZjDFR4 | -0.435582 | 0.7130852 |
| Kaempferol 3-O-β-rutinoside | Zj.jz019149004 | ZjDFR4 | -0.266137 | 0.8285054 |
| Quercetin O-rutinoside | Zj.jz019149004 | ZjDFR4 | 0.2569448 | 0.8345683 |
| Quercetin 3-O-glucoside | Zj.jz019149004 | ZjDFR4 | -0.217157 | 0.8606432 |
| Quercetin 3-O-rutinoside | Zj.jz019149004 | ZjDFR4 | 0.1844204 | 0.8819184 |
| Quercetin-3-β-O-galactoside | Zj.jz019149004 | ZjDFR4 | 0.9547794 | 0.1921824 |
| Kaempferol 3-O-β-rutinoside | Zj.jz019149004 | ZjDFR4 | 0.0034948 | 0.9977751 |
| Catechin | Zj.jz019149004 | ZjDFR4 | -0.260746 | 0.8320632 |
| Quercetin O-hexosyl-O-malonylhexoside | Zj.jz019149004 | ZjDFR4 | -0.127855 | 0.9183815 |
| Quercetin O-hexoside | Zj.jz019149004 | ZjDFR4 | -0.141079 | 0.9098859 |
| Quercetin-3-O-arabinofuranoside | Zj.jz019149004 | ZjDFR4 | 0.297135 | 0.8079377 |
| (+)-Gallocatechin (GC) | Zj.jz019149004 | ZjDFR4 | -0.080437 | 0.9487368 |
| Epigallocatechin (EGC) | Zj.jz019149004 | ZjDFR4 | 0.8543793 | 0.3478753 |
| L-Epicatechin | Zj.jz019149004 | ZjDFR4 | 0.993876 | 0.0704913 |
| Naringenin | Zj.jz019149004 | ZjDFR4 | -0.987565 | 0.100499 |
| Caffeic acid | Zj.jz019149004 | ZjDFR4 | 0.6277285 | 0.5679696 |
| Ferulic acid | Zj.jz019149004 | ZjDFR4 | 0.6010947 | 0.5894629 |
| Procyanidin A1 | Zj.jz019149004 | ZjDFR4 | 0.9931675 | 0.0744615 |
| Procyanidin B3 | Zj.jz019149004 | ZjDFR4 | 0.0722608 | 0.9539572 |
| Procyanidin A3 | Zj.jz019149004 | ZjDFR4 | -0.005666 | 0.9963928 |
| Procyanidin B2 | Zj.jz019149004 | ZjDFR4 | 0.7618242 | 0.448608 |
| Procyanidin B1 | Zj.jz019149004 | ZjDFR4 | 0.3329221 | 0.7839307 |
| Procyanidin A2 | Zj.jz019149004 | ZjDFR4 | 0.1655254 | 0.8941361 |
| procyanidin A | Zj.jz019149004 | ZjDFR4 | 0.2101873 | 0.8651852 |
| Procyanidin B | Zj.jz019149004 | ZjDFR4 | 0.2155438 | 0.8616952 |
| Dihydroquercetin | Zj.jz041429070 | ZjDFR5 | 0.9997304 | 0.0147832 |
| Dihydrokaempferol | Zj.jz041429070 | ZjDFR5 | -0.383703 | 0.7492976 |
| Cyanidin 3-O-glucoside | Zj.jz041429070 | ZjDFR5 | -0.892015 | 0.2985826 |
| Delphinidin 3-O-glucoside | Zj.jz041429070 | ZjDFR5 | 0.9996863 | 0.015947 |
| Cyanidin | Zj.jz041429070 | ZjDFR5 | 0.9680678 | 0.1613141 |
| Isorhamnetin O-hexoside | Zj.jz041429070 | ZjDFR5 | 0.9996239 | 0.0174602 |
| Isorhamnetin O-rutinoside | Zj.jz041429070 | ZjDFR5 | 0.9278562 | 0.2432993 |
| Myricitrin | Zj.jz041429070 | ZjDFR5 | -0.20077 | 0.8713115 |
| Narcissoside | Zj.jz041429070 | ZjDFR5 | 0.8280097 | 0.3789463 |
| Astragalin | Zj.jz041429070 | ZjDFR5 | 0.9698335 | 0.1567673 |
| Myricetin 3-O-galactoside | Zj.jz041429070 | ZjDFR5 | 0.9330255 | 0.2343171 |
| Kaempferol 3-O-galactoside | Zj.jz041429070 | ZjDFR5 | 0.9583631 | 0.184354 |
| Kaempferitrin | Zj.jz041429070 | ZjDFR5 | 0.4661264 | 0.6912984 |
| Quercetin | Zj.jz041429070 | ZjDFR5 | 0.996344 | 0.0544539 |
| Kaempferol 3-O-β-rutinoside | Zj.jz041429070 | ZjDFR5 | 0.9646096 | 0.1698741 |
| Quercetin O-rutinoside | Zj.jz041429070 | ZjDFR5 | 0.6995132 | 0.5068004 |
| Quercetin 3-O-glucoside | Zj.jz041429070 | ZjDFR5 | 0.9500752 | 0.2020119 |
| Quercetin 3-O-rutinoside | Zj.jz041429070 | ZjDFR5 | 0.7506818 | 0.4594503 |
| Quercetin-3-β-O-galactoside | Zj.jz041429070 | ZjDFR5 | -0.232202 | 0.8508137 |
| Kaempferol 3-O-β-rutinoside | Zj.jz041429070 | ZjDFR5 | 0.8578549 | 0.3435936 |
| Catechin | Zj.jz041429070 | ZjDFR5 | 0.9631209 | 0.1734319 |
| Quercetin O-hexosyl-O-malonylhexoside | Zj.jz041429070 | ZjDFR5 | 0.9179104 | 0.2597502 |
| Quercetin O-hexoside | Zj.jz041429070 | ZjDFR5 | 0.9231236 | 0.2512546 |
| Quercetin-3-O-arabinofuranoside | Zj.jz041429070 | ZjDFR5 | 0.6690166 | 0.533431 |
| (+)-Gallocatechin (GC) | Zj.jz041429070 | ZjDFR5 | 0.8979546 | 0.2901055 |
| Epigallocatechin (EGC) | Zj.jz041429070 | ZjDFR5 | 0.0102203 | 0.9934934 |
| L-Epicatechin | Zj.jz041429070 | ZjDFR5 | -0.41277 | 0.7291226 |
| Naringenin | Zj.jz041429070 | ZjDFR5 | 0.3693943 | 0.7591303 |
| Caffeic acid | Zj.jz041429070 | ZjDFR5 | -0.989877 | 0.0906617 |
| Ferulic acid | Zj.jz041429070 | ZjDFR5 | -0.994103 | 0.0691684 |
| Procyanidin A1 | Zj.jz041429070 | ZjDFR5 | -0.407082 | 0.7330928 |
| Procyanidin B3 | Zj.jz041429070 | ZjDFR5 | 0.8204809 | 0.3874115 |
| Procyanidin A3 | Zj.jz041429070 | ZjDFR5 | 0.8625266 | 0.3377615 |
| Procyanidin B2 | Zj.jz041429070 | ZjDFR5 | 0.1676556 | 0.8927607 |
| Procyanidin B1 | Zj.jz041429070 | ZjDFR5 | 0.6405197 | 0.557438 |
| Procyanidin A2 | Zj.jz041429070 | ZjDFR5 | 0.7632219 | 0.4472326 |
| procyanidin A | Zj.jz041429070 | ZjDFR5 | 0.7330594 | 0.4761835 |
| Procyanidin B | Zj.jz041429070 | ZjDFR5 | 0.7293196 | 0.4796735 |
| Dihydroquercetin | Zj.jz028857067 | ZjFLS1 | 0.9980065 | 0.0402048 |
| Dihydrokaempferol | Zj.jz028857067 | ZjFLS1 | -0.461937 | 0.6943095 |
| Cyanidin 3-O-glucoside | Zj.jz028857067 | ZjFLS1 | -0.927683 | 0.2435946 |
| Delphinidin 3-O-glucoside | Zj.jz028857067 | ZjFLS1 | 0.9937987 | 0.070935 |
| Cyanidin | Zj.jz028857067 | ZjFLS1 | 0.9860851 | 0.106326 |
| Isorhamnetin O-hexoside | Zj.jz028857067 | ZjFLS1 | 0.998263 | 0.0375278 |
| Isorhamnetin O-rutinoside | Zj.jz028857067 | ZjFLS1 | 0.9565696 | 0.1883113 |
| Myricitrin | Zj.jz028857067 | ZjFLS1 | -0.284532 | 0.8163235 |
| Narcissoside | Zj.jz028857067 | ZjFLS1 | 0.8732944 | 0.3239583 |
| Astragalin | Zj.jz028857067 | ZjFLS1 | 0.9872473 | 0.1017793 |
| Myricetin 3-O-galactoside | Zj.jz028857067 | ZjFLS1 | 0.9605872 | 0.1793291 |
| Kaempferol 3-O-galactoside | Zj.jz028857067 | ZjFLS1 | 0.9794243 | 0.129366 |
| Kaempferitrin | Zj.jz028857067 | ZjFLS1 | 0.5407112 | 0.6363103 |
| Quercetin | Zj.jz028857067 | ZjFLS1 | 0.9999996 | 0.0005341 |
| Kaempferol 3-O-β-rutinoside | Zj.jz028857067 | ZjFLS1 | 0.9837608 | 0.114886 |
| Quercetin O-rutinoside | Zj.jz028857067 | ZjFLS1 | 0.758554 | 0.4518124 |
| Quercetin 3-O-glucoside | Zj.jz028857067 | ZjFLS1 | 0.9734506 | 0.1470239 |
| Quercetin 3-O-rutinoside | Zj.jz028857067 | ZjFLS1 | 0.8048772 | 0.4044623 |
| Quercetin-3-β-O-galactoside | Zj.jz028857067 | ZjFLS1 | -0.147427 | 0.9058017 |
| Kaempferol 3-O-β-rutinoside | Zj.jz028857067 | ZjFLS1 | 0.8989891 | 0.2886055 |
| Catechin | Zj.jz028857067 | ZjFLS1 | 0.9827423 | 0.1184439 |
| Quercetin O-hexosyl-O-malonylhexoside | Zj.jz028857067 | ZjFLS1 | 0.9487184 | 0.2047622 |
| Quercetin O-hexoside | Zj.jz028857067 | ZjFLS1 | 0.9528524 | 0.1962665 |
| Quercetin-3-O-arabinofuranoside | Zj.jz028857067 | ZjFLS1 | 0.7306407 | 0.478443 |
| (+)-Gallocatechin (GC) | Zj.jz028857067 | ZjFLS1 | 0.9325724 | 0.2351175 |
| Epigallocatechin (EGC) | Zj.jz028857067 | ZjFLS1 | 0.0964453 | 0.9385054 |
| L-Epicatechin | Zj.jz028857067 | ZjFLS1 | -0.332656 | 0.7841106 |
| Naringenin | Zj.jz028857067 | ZjFLS1 | 0.2878511 | 0.8141184 |
| Caffeic acid | Zj.jz028857067 | ZjFLS1 | -0.973942 | 0.1456497 |
| Ferulic acid | Zj.jz028857067 | ZjFLS1 | -0.981043 | 0.1241564 |
| Procyanidin A1 | Zj.jz028857067 | ZjFLS1 | -0.326768 | 0.7880808 |
| Procyanidin B3 | Zj.jz028857067 | ZjFLS1 | 0.8667391 | 0.3324235 |
| Procyanidin A3 | Zj.jz028857067 | ZjFLS1 | 0.9029636 | 0.2827734 |
| Procyanidin B2 | Zj.jz028857067 | ZjFLS1 | 0.2520772 | 0.8377726 |
| Procyanidin B1 | Zj.jz028857067 | ZjFLS1 | 0.7043804 | 0.5024499 |
| Procyanidin A2 | Zj.jz028857067 | ZjFLS1 | 0.8161173 | 0.3922446 |
| procyanidin A | Zj.jz028857067 | ZjFLS1 | 0.7890027 | 0.4211954 |
| Procyanidin B | Zj.jz028857067 | ZjFLS1 | 0.7856227 | 0.4246855 |
| Dihydroquercetin | Zj.jz044891001 | ZjFLS2 | 0.9998642 | 0.0104911 |
| Dihydrokaempferol | Zj.jz044891001 | ZjFLS2 | -0.420053 | 0.7240232 |
| Cyanidin 3-O-glucoside | Zj.jz044891001 | ZjFLS2 | -0.909253 | 0.2733082 |
| Delphinidin 3-O-glucoside | Zj.jz044891001 | ZjFLS2 | 0.9979044 | 0.0412214 |
| Cyanidin | Zj.jz044891001 | ZjFLS2 | 0.9772549 | 0.1360397 |
| Isorhamnetin O-hexoside | Zj.jz044891001 | ZjFLS2 | 0.9999247 | 0.0078142 |
| Isorhamnetin O-rutinoside | Zj.jz044891001 | ZjFLS2 | 0.9419271 | 0.2180249 |
| Myricitrin | Zj.jz044891001 | ZjFLS2 | -0.239494 | 0.8460371 |
| Narcissoside | Zj.jz044891001 | ZjFLS2 | 0.8496123 | 0.353672 |
| Astragalin | Zj.jz044891001 | ZjFLS2 | 0.9787446 | 0.1314929 |
| Myricetin 3-O-galactoside | Zj.jz044891001 | ZjFLS2 | 0.9465713 | 0.2090428 |
| Kaempferol 3-O-galactoside | Zj.jz044891001 | ZjFLS2 | 0.9689417 | 0.1590797 |
| Kaempferitrin | Zj.jz044891001 | ZjFLS2 | 0.500874 | 0.666024 |
| Quercetin | Zj.jz044891001 | ZjFLS2 | 0.9989498 | 0.0291795 |
| Kaempferol 3-O-β-rutinoside | Zj.jz044891001 | ZjFLS2 | 0.9743152 | 0.1445997 |
| Quercetin O-rutinoside | Zj.jz044891001 | ZjFLS2 | 0.7273256 | 0.4815261 |
| Quercetin 3-O-glucoside | Zj.jz044891001 | ZjFLS2 | 0.9617108 | 0.1767375 |
| Quercetin 3-O-rutinoside | Zj.jz044891001 | ZjFLS2 | 0.7763124 | 0.4341759 |
| Quercetin-3-β-O-galactoside | Zj.jz044891001 | ZjFLS2 | -0.193414 | 0.8760881 |
| Kaempferol 3-O-β-rutinoside | Zj.jz044891001 | ZjFLS2 | 0.8775756 | 0.3183192 |
| Catechin | Zj.jz044891001 | ZjFLS2 | 0.9730415 | 0.1481575 |
| Quercetin O-hexosyl-O-malonylhexoside | Zj.jz044891001 | ZjFLS2 | 0.9329358 | 0.2344758 |
| Quercetin O-hexoside | Zj.jz044891001 | ZjFLS2 | 0.9376573 | 0.2259802 |
| Quercetin-3-O-arabinofuranoside | Zj.jz044891001 | ZjFLS2 | 0.6979893 | 0.5081566 |
| (+)-Gallocatechin (GC) | Zj.jz044891001 | ZjFLS2 | 0.9147144 | 0.2648311 |
| Epigallocatechin (EGC) | Zj.jz044891001 | ZjFLS2 | 0.0499007 | 0.968219 |
| L-Epicatechin | Zj.jz044891001 | ZjFLS2 | -0.376293 | 0.754397 |
| Naringenin | Zj.jz044891001 | ZjFLS2 | 0.33222 | 0.7844047 |
| Caffeic acid | Zj.jz044891001 | ZjFLS2 | -0.983463 | 0.1159361 |
| Ferulic acid | Zj.jz044891001 | ZjFLS2 | -0.989016 | 0.0944428 |
| Procyanidin A1 | Zj.jz044891001 | ZjFLS2 | -0.370508 | 0.7583672 |
| Procyanidin B3 | Zj.jz044891001 | ZjFLS2 | 0.8425244 | 0.3621371 |
| Procyanidin A3 | Zj.jz044891001 | ZjFLS2 | 0.8819308 | 0.3124871 |
| Procyanidin B2 | Zj.jz044891001 | ZjFLS2 | 0.2066522 | 0.8674863 |
| Procyanidin B1 | Zj.jz044891001 | ZjFLS2 | 0.670495 | 0.5321636 |
| Procyanidin A2 | Zj.jz044891001 | ZjFLS2 | 0.788266 | 0.4219583 |
| procyanidin A | Zj.jz044891001 | ZjFLS2 | 0.7594778 | 0.4509091 |
| Procyanidin B | Zj.jz044891001 | ZjFLS2 | 0.7559001 | 0.4543991 |
| Dihydroquercetin | Zj.jz017257032 | ZjANS1 | 0.9905495 | 0.087592 |
| Dihydrokaempferol | Zj.jz017257032 | ZjANS1 | -0.526614 | 0.6469223 |
| Cyanidin 3-O-glucoside | Zj.jz017257032 | ZjANS1 | -0.952881 | 0.1962073 |
| Delphinidin 3-O-glucoside | Zj.jz017257032 | ZjANS1 | 0.9827777 | 0.1183222 |
| Cyanidin | Zj.jz017257032 | ZjANS1 | 0.9957174 | 0.0589388 |
| Isorhamnetin O-hexoside | Zj.jz017257032 | ZjANS1 | 0.9911175 | 0.084915 |
| Isorhamnetin O-rutinoside | Zj.jz017257032 | ZjANS1 | 0.9755991 | 0.1409241 |
| Myricitrin | Zj.jz017257032 | ZjANS1 | -0.355037 | 0.7689363 |
| Narcissoside | Zj.jz017257032 | ZjANS1 | 0.9071072 | 0.2765711 |
| Astragalin | Zj.jz017257032 | ZjANS1 | 0.9963523 | 0.0543921 |
| Myricetin 3-O-galactoside | Zj.jz017257032 | ZjANS1 | 0.9785997 | 0.1319419 |
| Kaempferol 3-O-galactoside | Zj.jz017257032 | ZjANS1 | 0.9917203 | 0.0819788 |
| Kaempferitrin | Zj.jz017257032 | ZjANS1 | 0.6017721 | 0.5889231 |
| Quercetin | Zj.jz017257032 | ZjANS1 | 0.9971682 | 0.0479213 |
| Kaempferol 3-O-β-rutinoside | Zj.jz017257032 | ZjANS1 | 0.9943844 | 0.0674988 |
| Quercetin O-rutinoside | Zj.jz017257032 | ZjANS1 | 0.8049117 | 0.4044252 |
| Quercetin 3-O-glucoside | Zj.jz017257032 | ZjANS1 | 0.9877775 | 0.0996366 |
| Quercetin 3-O-rutinoside | Zj.jz017257032 | ZjANS1 | 0.8467809 | 0.3570751 |
| Quercetin-3-β-O-galactoside | Zj.jz017257032 | ZjANS1 | -0.073464 | 0.953189 |
| Kaempferol 3-O-β-rutinoside | Zj.jz017257032 | ZjANS1 | 0.9290703 | 0.2412183 |
| Catechin | Zj.jz017257032 | ZjANS1 | 0.9937774 | 0.0710567 |
| Quercetin O-hexosyl-O-malonylhexoside | Zj.jz017257032 | ZjANS1 | 0.9696004 | 0.157375 |
| Quercetin O-hexoside | Zj.jz017257032 | ZjANS1 | 0.9727794 | 0.1488793 |
| Quercetin-3-O-arabinofuranoside | Zj.jz017257032 | ZjANS1 | 0.7793924 | 0.4310558 |
| (+)-Gallocatechin (GC) | Zj.jz017257032 | ZjANS1 | 0.9568353 | 0.1877303 |
| Epigallocatechin (EGC) | Zj.jz017257032 | ZjANS1 | 0.1701985 | 0.8911182 |
| L-Epicatechin | Zj.jz017257032 | ZjANS1 | -0.261603 | 0.8314978 |
| Naringenin | Zj.jz017257032 | ZjANS1 | 0.2158346 | 0.8615056 |
| Caffeic acid | Zj.jz017257032 | ZjANS1 | -0.954379 | 0.1930369 |
| Ferulic acid | Zj.jz017257032 | ZjANS1 | -0.963915 | 0.1715436 |
| Procyanidin A1 | Zj.jz017257032 | ZjANS1 | -0.255579 | 0.835468 |
| Procyanidin B3 | Zj.jz017257032 | ZjANS1 | 0.9014305 | 0.2850363 |
| Procyanidin A3 | Zj.jz017257032 | ZjANS1 | 0.93242 | 0.2353862 |
| Procyanidin B2 | Zj.jz017257032 | ZjANS1 | 0.3233446 | 0.7903854 |
| Procyanidin B1 | Zj.jz017257032 | ZjANS1 | 0.7552172 | 0.4550627 |
| Procyanidin A2 | Zj.jz017257032 | ZjANS1 | 0.856833 | 0.3448574 |
| procyanidin A | Zj.jz017257032 | ZjANS1 | 0.8325082 | 0.3738082 |
| Procyanidin B | Zj.jz017257032 | ZjANS1 | 0.8294585 | 0.3772983 |
| Dihydroquercetin | Zj.jz022481171 | ZjANS2 | 0.9951487 | 0.0627339 |
| Dihydrokaempferol | Zj.jz022481171 | ZjANS2 | -0.313125 | 0.7972482 |
| Cyanidin 3-O-glucoside | Zj.jz022481171 | ZjANS2 | -0.855473 | 0.3465332 |
| Delphinidin 3-O-glucoside | Zj.jz022481171 | ZjANS2 | 0.9987367 | 0.0320036 |
| Cyanidin | Zj.jz022481171 | ZjANS2 | 0.9464589 | 0.2092647 |
| Isorhamnetin O-hexoside | Zj.jz022481171 | ZjANS2 | 0.9947262 | 0.0654109 |
| Isorhamnetin O-rutinoside | Zj.jz022481171 | ZjANS2 | 0.8971621 | 0.29125 |
| Myricitrin | Zj.jz022481171 | ZjANS2 | -0.126483 | 0.9192621 |
| Narcissoside | Zj.jz022481171 | ZjANS2 | 0.7834687 | 0.426897 |
| Astragalin | Zj.jz022481171 | ZjANS2 | 0.9487403 | 0.2047179 |
| Myricetin 3-O-galactoside | Zj.jz022481171 | ZjANS2 | 0.9033046 | 0.2822678 |
| Kaempferol 3-O-galactoside | Zj.jz022481171 | ZjANS2 | 0.9341583 | 0.2323047 |
| Kaempferitrin | Zj.jz022481171 | ZjANS2 | 0.3982303 | 0.739249 |
| Quercetin | Zj.jz022481171 | ZjANS2 | 0.9870904 | 0.1024045 |
| Kaempferol 3-O-β-rutinoside | Zj.jz022481171 | ZjANS2 | 0.9420327 | 0.2178247 |
| Quercetin O-rutinoside | Zj.jz022481171 | ZjANS2 | 0.6437551 | 0.5547511 |
| Quercetin 3-O-glucoside | Zj.jz022481171 | ZjANS2 | 0.9239021 | 0.2499625 |
| Quercetin 3-O-rutinoside | Zj.jz022481171 | ZjANS2 | 0.6988388 | 0.5074009 |
| Quercetin-3-β-O-galactoside | Zj.jz022481171 | ZjANS2 | -0.304737 | 0.8028631 |
| Kaempferol 3-O-β-rutinoside | Zj.jz022481171 | ZjANS2 | 0.8167526 | 0.3915442 |
| Catechin | Zj.jz022481171 | ZjANS2 | 0.9401429 | 0.2213826 |
| Quercetin O-hexosyl-O-malonylhexoside | Zj.jz022481171 | ZjANS2 | 0.8854498 | 0.3077008 |
| Quercetin O-hexoside | Zj.jz022481171 | ZjANS2 | 0.8915726 | 0.2992052 |
| Quercetin-3-O-arabinofuranoside | Zj.jz022481171 | ZjANS2 | 0.6111908 | 0.5813816 |
| (+)-Gallocatechin (GC) | Zj.jz022481171 | ZjANS2 | 0.8622923 | 0.3380562 |
| Epigallocatechin (EGC) | Zj.jz022481171 | ZjANS2 | -0.065054 | 0.9585559 |
| L-Epicatechin | Zj.jz022481171 | ZjANS2 | -0.48014 | 0.681172 |
| Naringenin | Zj.jz022481171 | ZjANS2 | 0.4382743 | 0.7111797 |
| Caffeic acid | Zj.jz022481171 | ZjANS2 | -0.99775 | 0.0427111 |
| Ferulic acid | Zj.jz022481171 | ZjANS2 | -0.999445 | 0.0212178 |
| Procyanidin A1 | Zj.jz022481171 | ZjANS2 | -0.47466 | 0.6851422 |
| Procyanidin B3 | Zj.jz022481171 | ZjANS2 | 0.7751365 | 0.4353621 |
| Procyanidin A3 | Zj.jz022481171 | ZjANS2 | 0.822004 | 0.3857121 |
| Procyanidin B2 | Zj.jz022481171 | ZjANS2 | 0.0929959 | 0.9407113 |
| Procyanidin B1 | Zj.jz022481171 | ZjANS2 | 0.5809165 | 0.6053886 |
| Procyanidin A2 | Zj.jz022481171 | ZjANS2 | 0.7124365 | 0.4951833 |
| procyanidin A | Zj.jz022481171 | ZjANS2 | 0.6797989 | 0.5241341 |
| Procyanidin B | Zj.jz022481171 | ZjANS2 | 0.6757681 | 0.5276241 |
| Dihydroquercetin | Zj.jz017257031 | ZjANS3 | 0.8234593 | 0.3840823 |
| Dihydrokaempferol | Zj.jz017257031 | ZjANS3 | 0.1852154 | 0.8814034 |
| Cyanidin 3-O-glucoside | Zj.jz017257031 | ZjANS3 | -0.498346 | 0.6678816 |
| Delphinidin 3-O-glucoside | Zj.jz017257031 | ZjANS3 | 0.8498772 | 0.353352 |
| Cyanidin | Zj.jz017257031 | ZjANS3 | 0.6722999 | 0.5306131 |
| Isorhamnetin O-hexoside | Zj.jz017257031 | ZjANS3 | 0.8210662 | 0.3867593 |
| Isorhamnetin O-rutinoside | Zj.jz017257031 | ZjANS3 | 0.5716612 | 0.6125983 |
| Myricitrin | Zj.jz017257031 | ZjANS3 | 0.369016 | 0.7593895 |
| Narcissoside | Zj.jz017257031 | ZjANS3 | 0.3852284 | 0.7482454 |
| Astragalin | Zj.jz017257031 | ZjANS3 | 0.6775698 | 0.5260663 |
| Myricetin 3-O-galactoside | Zj.jz017257031 | ZjANS3 | 0.5831804 | 0.6036162 |
| Kaempferol 3-O-galactoside | Zj.jz017257031 | ZjANS3 | 0.645074 | 0.5536531 |
| Kaempferitrin | Zj.jz017257031 | ZjANS3 | -0.095042 | 0.9394026 |
| Quercetin | Zj.jz017257031 | ZjANS3 | 0.7865282 | 0.4237529 |
| Kaempferol 3-O-β-rutinoside | Zj.jz017257031 | ZjANS3 | 0.6622856 | 0.5391731 |
| Quercetin O-rutinoside | Zj.jz017257031 | ZjANS3 | 0.1933962 | 0.8760995 |
| Quercetin 3-O-glucoside | Zj.jz017257031 | ZjANS3 | 0.6236343 | 0.5713109 |
| Quercetin 3-O-rutinoside | Zj.jz017257031 | ZjANS3 | 0.2657675 | 0.8287493 |
| Quercetin-3-β-O-galactoside | Zj.jz017257031 | ZjANS3 | -0.727338 | 0.4815147 |
| Kaempferol 3-O-β-rutinoside | Zj.jz017257031 | ZjANS3 | 0.4358543 | 0.7128926 |
| Catechin | Zj.jz017257031 | ZjANS3 | 0.658088 | 0.5427309 |
| Quercetin O-hexosyl-O-malonylhexoside | Zj.jz017257031 | ZjANS3 | 0.5502705 | 0.6290492 |
| Quercetin O-hexoside | Zj.jz017257031 | ZjANS3 | 0.561364 | 0.6205536 |
| Quercetin-3-O-arabinofuranoside | Zj.jz017257031 | ZjANS3 | 0.1521975 | 0.90273 |
| (+)-Gallocatechin (GC) | Zj.jz017257031 | ZjANS3 | 0.5098463 | 0.6594045 |
| Epigallocatechin (EGC) | Zj.jz017257031 | ZjANS3 | -0.539525 | 0.6372076 |
| L-Epicatechin | Zj.jz017257031 | ZjANS3 | -0.844476 | 0.3598236 |
| Naringenin | Zj.jz017257031 | ZjANS3 | 0.818302 | 0.3898313 |
| Caffeic acid | Zj.jz017257031 | ZjANS3 | -0.905736 | 0.2786373 |
| Ferulic acid | Zj.jz017257031 | ZjANS3 | -0.890913 | 0.3001306 |
| Procyanidin A1 | Zj.jz017257031 | ZjANS3 | -0.84112 | 0.3637938 |
| Procyanidin B3 | Zj.jz017257031 | ZjANS3 | 0.3729239 | 0.7567105 |
| Procyanidin A3 | Zj.jz017257031 | ZjANS3 | 0.444081 | 0.7070605 |
| Procyanidin B2 | Zj.jz017257031 | ZjANS3 | -0.400115 | 0.7379403 |
| Procyanidin B1 | Zj.jz017257031 | ZjANS3 | 0.1148275 | 0.926737 |
| Procyanidin A2 | Zj.jz017257031 | ZjANS3 | 0.2842187 | 0.8165317 |
| procyanidin A | Zj.jz017257031 | ZjANS3 | 0.2403395 | 0.8454825 |
| Procyanidin B | Zj.jz017257031 | ZjANS3 | 0.2350144 | 0.8489725 |
| Dihydroquercetin | Zj.jz040731058 | ZjANS4 | -0.529998 | 0.6443856 |
| Dihydrokaempferol | Zj.jz040731058 | ZjANS4 | 0.9899951 | 0.0901288 |
| Cyanidin 3-O-glucoside | Zj.jz040731058 | ZjANS4 | 0.8438341 | 0.3605862 |
| Delphinidin 3-O-glucoside | Zj.jz040731058 | ZjANS4 | -0.488462 | 0.6751158 |
| Cyanidin | Zj.jz040731058 | ZjANS4 | -0.709486 | 0.4978547 |
| Isorhamnetin O-hexoside | Zj.jz040731058 | ZjANS4 | -0.533559 | 0.6417086 |
| Isorhamnetin O-rutinoside | Zj.jz040731058 | ZjANS4 | -0.794115 | 0.4158695 |
| Myricitrin | Zj.jz040731058 | ZjANS4 | 0.9449898 | 0.2121427 |
| Narcissoside | Zj.jz040731058 | ZjANS4 | -0.904678 | 0.2802225 |
| Astragalin | Zj.jz040731058 | ZjANS4 | -0.704434 | 0.5024015 |
| Myricetin 3-O-galactoside | Zj.jz040731058 | ZjANS4 | -0.785461 | 0.4248517 |
| Kaempferol 3-O-galactoside | Zj.jz040731058 | ZjANS4 | -0.73452 | 0.4748148 |
| Kaempferitrin | Zj.jz040731058 | ZjANS4 | -0.998727 | 0.0321296 |
| Quercetin | Zj.jz040731058 | ZjANS4 | -0.581777 | 0.6047149 |
| Kaempferol 3-O-β-rutinoside | Zj.jz040731058 | ZjANS4 | -0.718897 | 0.4892947 |
| Quercetin O-rutinoside | Zj.jz040731058 | ZjANS4 | -0.971495 | 0.1523684 |
| Quercetin 3-O-glucoside | Zj.jz040731058 | ZjANS4 | -0.753057 | 0.4571569 |
| Quercetin 3-O-rutinoside | Zj.jz040731058 | ZjANS4 | -0.951193 | 0.1997185 |
| Quercetin-3-β-O-galactoside | Zj.jz040731058 | ZjANS4 | -0.718107 | 0.4900175 |
| Kaempferol 3-O-β-rutinoside | Zj.jz040731058 | ZjANS4 | -0.879634 | 0.3155752 |
| Catechin | Zj.jz040731058 | ZjANS4 | -0.72277 | 0.4857369 |
| Quercetin O-hexosyl-O-malonylhexoside | Zj.jz040731058 | ZjANS4 | -0.809553 | 0.3994186 |
| Quercetin O-hexoside | Zj.jz040731058 | ZjANS4 | -0.801647 | 0.4079143 |
| Quercetin-3-O-arabinofuranoside | Zj.jz040731058 | ZjANS4 | -0.980559 | 0.1257378 |
| (+)-Gallocatechin (GC) | Zj.jz040731058 | ZjANS4 | -0.836614 | 0.3690633 |
| Epigallocatechin (EGC) | Zj.jz040731058 | ZjANS4 | -0.865246 | 0.3343246 |
| L-Epicatechin | Zj.jz040731058 | ZjANS4 | -0.572807 | 0.6117086 |
| Naringenin | Zj.jz040731058 | ZjANS4 | 0.6107938 | 0.5817008 |
| Caffeic acid | Zj.jz040731058 | ZjANS4 | 0.3829294 | 0.7498305 |
| Ferulic acid | Zj.jz040731058 | ZjANS4 | 0.4138934 | 0.7283372 |
| Procyanidin A1 | Zj.jz040731058 | ZjANS4 | -0.577908 | 0.6077384 |
| Procyanidin B3 | Zj.jz040731058 | ZjANS4 | -0.910264 | 0.2717573 |
| Procyanidin A3 | Zj.jz040731058 | ZjANS4 | -0.87524 | 0.3214074 |
| Procyanidin B2 | Zj.jz040731058 | ZjANS4 | -0.933435 | 0.2335919 |
| Procyanidin B1 | Zj.jz040731058 | ZjANS4 | -0.987259 | 0.1017309 |
| Procyanidin A2 | Zj.jz040731058 | ZjANS4 | -0.945096 | 0.2119362 |
| procyanidin A | Zj.jz040731058 | ZjANS4 | -0.958975 | 0.1829853 |
| Procyanidin B | Zj.jz040731058 | ZjANS4 | -0.960515 | 0.1794953 |
| Dihydroquercetin | Zj.jz012195006 | ZjLAR1 | 0.9958799 | 0.0578092 |
| Dihydrokaempferol | Zj.jz012195006 | ZjLAR1 | -0.320462 | 0.7923236 |
| Cyanidin 3-O-glucoside | Zj.jz012195006 | ZjLAR1 | -0.859453 | 0.3416086 |
| Delphinidin 3-O-glucoside | Zj.jz012195006 | ZjLAR1 | 0.9990955 | 0.027079 |
| Cyanidin | Zj.jz012195006 | ZjLAR1 | 0.9489277 | 0.2043401 |
| Isorhamnetin O-hexoside | Zj.jz012195006 | ZjLAR1 | 0.9954898 | 0.0604862 |
| Isorhamnetin O-rutinoside | Zj.jz012195006 | ZjLAR1 | 0.900552 | 0.2863253 |
| Myricitrin | Zj.jz012195006 | ZjLAR1 | -0.134153 | 0.9143375 |
| Narcissoside | Zj.jz012195006 | ZjLAR1 | 0.7882524 | 0.4219723 |
| Astragalin | Zj.jz012195006 | ZjLAR1 | 0.9511568 | 0.1997933 |
| Myricetin 3-O-galactoside | Zj.jz012195006 | ZjLAR1 | 0.9065961 | 0.2773431 |
| Kaempferol 3-O-galactoside | Zj.jz012195006 | ZjLAR1 | 0.9368908 | 0.22738 |
| Kaempferitrin | Zj.jz012195006 | ZjLAR1 | 0.405314 | 0.7343244 |
| Quercetin | Zj.jz012195006 | ZjLAR1 | 0.9882999 | 0.0974799 |
| Kaempferol 3-O-β-rutinoside | Zj.jz012195006 | ZjLAR1 | 0.9446 | 0.2129001 |
| Quercetin O-rutinoside | Zj.jz012195006 | ZjLAR1 | 0.6496553 | 0.5498264 |
| Quercetin 3-O-glucoside | Zj.jz012195006 | ZjLAR1 | 0.9268343 | 0.2450379 |
| Quercetin 3-O-rutinoside | Zj.jz012195006 | ZjLAR1 | 0.704351 | 0.5024763 |
| Quercetin-3-β-O-galactoside | Zj.jz012195006 | ZjLAR1 | -0.29736 | 0.8077877 |
| Kaempferol 3-O-β-rutinoside | Zj.jz012195006 | ZjLAR1 | 0.8211914 | 0.3866196 |
| Catechin | Zj.jz012195006 | ZjLAR1 | 0.9427509 | 0.2164579 |
| Quercetin O-hexosyl-O-malonylhexoside | Zj.jz012195006 | ZjLAR1 | 0.8890183 | 0.3027762 |
| Quercetin O-hexoside | Zj.jz012195006 | ZjLAR1 | 0.8950492 | 0.2942805 |
| Quercetin-3-O-arabinofuranoside | Zj.jz012195006 | ZjLAR1 | 0.617295 | 0.576457 |
| (+)-Gallocatechin (GC) | Zj.jz012195006 | ZjLAR1 | 0.8661839 | 0.3331315 |
| Epigallocatechin (EGC) | Zj.jz012195006 | ZjLAR1 | -0.057333 | 0.9634806 |
| L-Epicatechin | Zj.jz012195006 | ZjLAR1 | -0.47334 | 0.6860966 |
| Naringenin | Zj.jz012195006 | ZjLAR1 | 0.4313082 | 0.7161043 |
| Caffeic acid | Zj.jz012195006 | ZjLAR1 | -0.997202 | 0.0476357 |
| Ferulic acid | Zj.jz012195006 | ZjLAR1 | -0.999157 | 0.0261424 |
| Procyanidin A1 | Zj.jz012195006 | ZjLAR1 | -0.467837 | 0.6900668 |
| Procyanidin B3 | Zj.jz012195006 | ZjLAR1 | 0.7800005 | 0.4304375 |
| Procyanidin A3 | Zj.jz012195006 | ZjLAR1 | 0.8263847 | 0.3807875 |
| Procyanidin B2 | Zj.jz012195006 | ZjLAR1 | 0.1006951 | 0.9357867 |
| Procyanidin B1 | Zj.jz012195006 | ZjLAR1 | 0.5871955 | 0.6004639 |
| Procyanidin A2 | Zj.jz012195006 | ZjLAR1 | 0.7178435 | 0.4902586 |
| procyanidin A | Zj.jz012195006 | ZjLAR1 | 0.6854518 | 0.5192095 |
| Procyanidin B | Zj.jz012195006 | ZjLAR1 | 0.6814499 | 0.5226995 |
| Dihydroquercetin | Zj.jz000565035 | ZjLAR2 | -0.619923 | 0.5743274 |
| Dihydrokaempferol | Zj.jz000565035 | ZjLAR2 | -0.466321 | 0.6911583 |
| Cyanidin 3-O-glucoside | Zj.jz000565035 | ZjLAR2 | 0.221014 | 0.8581267 |
| Delphinidin 3-O-glucoside | Zj.jz000565035 | ZjLAR2 | -0.657063 | 0.5435971 |
| Cyanidin | Zj.jz000565035 | ZjLAR2 | -0.424559 | 0.7208582 |
| Isorhamnetin O-hexoside | Zj.jz000565035 | ZjLAR2 | -0.616618 | 0.5770044 |
| Isorhamnetin O-rutinoside | Zj.jz000565035 | ZjLAR2 | -0.304766 | 0.8028435 |
| Myricitrin | Zj.jz000565035 | ZjLAR2 | -0.626291 | 0.5691444 |
| Narcissoside | Zj.jz000565035 | ZjLAR2 | -0.096469 | 0.9384905 |
| Astragalin | Zj.jz000565035 | ZjLAR2 | -0.431015 | 0.7163114 |
| Myricetin 3-O-galactoside | Zj.jz000565035 | ZjLAR2 | -0.318173 | 0.7938613 |
| Kaempferol 3-O-galactoside | Zj.jz000565035 | ZjLAR2 | -0.391521 | 0.7438982 |
| Kaempferitrin | Zj.jz000565035 | ZjLAR2 | 0.3839058 | 0.7491575 |
| Quercetin | Zj.jz000565035 | ZjLAR2 | -0.569856 | 0.613998 |
| Kaempferol 3-O-β-rutinoside | Zj.jz000565035 | ZjLAR2 | -0.412347 | 0.7294182 |
| Quercetin O-rutinoside | Zj.jz000565035 | ZjLAR2 | 0.1040253 | 0.9336554 |
| Quercetin 3-O-glucoside | Zj.jz000565035 | ZjLAR2 | -0.365851 | 0.761556 |
| Quercetin 3-O-rutinoside | Zj.jz000565035 | ZjLAR2 | 0.0298319 | 0.9810056 |
| Quercetin-3-β-O-galactoside | Zj.jz000565035 | ZjLAR2 | 0.8971484 | 0.2912696 |
| Kaempferol 3-O-β-rutinoside | Zj.jz000565035 | ZjLAR2 | -0.151565 | 0.9031377 |
| Catechin | Zj.jz000565035 | ZjLAR2 | -0.407249 | 0.7329761 |
| Quercetin O-hexosyl-O-malonylhexoside | Zj.jz000565035 | ZjLAR2 | -0.280055 | 0.8192943 |
| Quercetin O-hexoside | Zj.jz000565035 | ZjLAR2 | -0.292841 | 0.8107987 |
| Quercetin-3-O-arabinofuranoside | Zj.jz000565035 | ZjLAR2 | 0.1455264 | 0.9070249 |
| (+)-Gallocatechin (GC) | Zj.jz000565035 | ZjLAR2 | -0.23398 | 0.8496496 |
| Epigallocatechin (EGC) | Zj.jz000565035 | ZjLAR2 | 0.7634961 | 0.4469624 |
| L-Epicatechin | Zj.jz000565035 | ZjLAR2 | 0.9647319 | 0.1695785 |
| Naringenin | Zj.jz000565035 | ZjLAR2 | -0.951257 | 0.1995862 |
| Caffeic acid | Zj.jz000565035 | ZjLAR2 | 0.7408114 | 0.4688824 |
| Ferulic acid | Zj.jz000565035 | ZjLAR2 | 0.7177155 | 0.4903757 |
| Procyanidin A1 | Zj.jz000565035 | ZjLAR2 | 0.9630715 | 0.1735487 |
| Procyanidin B3 | Zj.jz000565035 | ZjLAR2 | -0.083226 | 0.9469556 |
| Procyanidin A3 | Zj.jz000565035 | ZjLAR2 | -0.160613 | 0.8973056 |
| Procyanidin B2 | Zj.jz000565035 | ZjLAR2 | 0.6521967 | 0.5476952 |
| Procyanidin B1 | Zj.jz000565035 | ZjLAR2 | 0.1827227 | 0.8830179 |
| Procyanidin A2 | Zj.jz000565035 | ZjLAR2 | 0.0106447 | 0.9932232 |
| procyanidin A | Zj.jz000565035 | ZjLAR2 | 0.0560913 | 0.9642724 |
| Procyanidin B | Zj.jz000565035 | ZjLAR2 | 0.061564 | 0.9607824 |
| Dihydroquercetin | Zj.jz008511027 | ZjANR1 | 0.993465 | 0.0728208 |
| Dihydrokaempferol | Zj.jz008511027 | ZjANR1 | -0.50675 | 0.6616936 |
| Cyanidin 3-O-glucoside | Zj.jz008511027 | ZjANR1 | -0.945586 | 0.2109786 |
| Delphinidin 3-O-glucoside | Zj.jz008511027 | ZjANR1 | 0.9868004 | 0.103551 |
| Cyanidin | Zj.jz008511027 | ZjANR1 | 0.9933046 | 0.0737101 |
| Isorhamnetin O-hexoside | Zj.jz008511027 | ZjANR1 | 0.9939362 | 0.0701438 |
| Isorhamnetin O-rutinoside | Zj.jz008511027 | ZjANR1 | 0.9702426 | 0.1556953 |
| Myricitrin | Zj.jz008511027 | ZjANR1 | -0.333253 | 0.7837075 |
| Narcissoside | Zj.jz008511027 | ZjANR1 | 0.8970979 | 0.2913423 |
| Astragalin | Zj.jz008511027 | ZjANR1 | 0.9941043 | 0.0691633 |
| Myricetin 3-O-galactoside | Zj.jz008511027 | ZjANR1 | 0.9735622 | 0.1467131 |
| Kaempferol 3-O-galactoside | Zj.jz008511027 | ZjANR1 | 0.9884741 | 0.09675 |
| Kaempferitrin | Zj.jz008511027 | ZjANR1 | 0.5830806 | 0.6036944 |
| Quercetin | Zj.jz008511027 | ZjANR1 | 0.9986446 | 0.0331501 |
| Kaempferol 3-O-β-rutinoside | Zj.jz008511027 | ZjANR1 | 0.9916615 | 0.0822701 |
| Quercetin O-rutinoside | Zj.jz008511027 | ZjANR1 | 0.790928 | 0.4191964 |
| Quercetin 3-O-glucoside | Zj.jz008511027 | ZjANR1 | 0.9838953 | 0.1144079 |
| Quercetin 3-O-rutinoside | Zj.jz008511027 | ZjANR1 | 0.8342116 | 0.3718463 |
| Quercetin-3-β-O-galactoside | Zj.jz008511027 | ZjANR1 | -0.096582 | 0.9384177 |
| Kaempferol 3-O-β-rutinoside | Zj.jz008511027 | ZjANR1 | 0.9202383 | 0.2559896 |
| Catechin | Zj.jz008511027 | ZjANR1 | 0.9909258 | 0.0858279 |
| Quercetin O-hexosyl-O-malonylhexoside | Zj.jz008511027 | ZjANR1 | 0.9636624 | 0.1721462 |
| Quercetin O-hexoside | Zj.jz008511027 | ZjANR1 | 0.9671412 | 0.1636506 |
| Quercetin-3-O-arabinofuranoside | Zj.jz008511027 | ZjANR1 | 0.7646467 | 0.445827 |
| (+)-Gallocatechin (GC) | Zj.jz008511027 | ZjANR1 | 0.9498349 | 0.2025015 |
| Epigallocatechin (EGC) | Zj.jz008511027 | ZjANR1 | 0.1472907 | 0.9058894 |
| L-Epicatechin | Zj.jz008511027 | ZjANR1 | -0.283925 | 0.8167266 |
| Naringenin | Zj.jz008511027 | ZjANR1 | 0.2384302 | 0.8467343 |
| Caffeic acid | Zj.jz008511027 | ZjANR1 | -0.96105 | 0.1782657 |
| Ferulic acid | Zj.jz008511027 | ZjANR1 | -0.969832 | 0.1567724 |
| Procyanidin A1 | Zj.jz008511027 | ZjANR1 | -0.27794 | 0.8206968 |
| Procyanidin B3 | Zj.jz008511027 | ZjANR1 | 0.8911438 | 0.2998075 |
| Procyanidin A3 | Zj.jz008511027 | ZjANR1 | 0.9237849 | 0.2501575 |
| Procyanidin B2 | Zj.jz008511027 | ZjANR1 | 0.3013033 | 0.8051567 |
| Procyanidin B1 | Zj.jz008511027 | ZjANR1 | 0.7398066 | 0.469834 |
| Procyanidin A2 | Zj.jz008511027 | ZjANR1 | 0.8446403 | 0.3596286 |
| procyanidin A | Zj.jz008511027 | ZjANR1 | 0.8194307 | 0.3885795 |
| Procyanidin B | Zj.jz008511027 | ZjANR1 | 0.8162762 | 0.3920695 |
| Dihydroquercetin | Zj.jz005267025 | ZjUFGT1 | 0.2449725 | 0.8424421 |
| Dihydrokaempferol | Zj.jz005267025 | ZjUFGT1 | -0.985664 | 0.1079278 |
| Cyanidin 3-O-glucoside | Zj.jz005267025 | ZjUFGT1 | -0.639065 | 0.5586428 |
| Delphinidin 3-O-glucoside | Zj.jz005267025 | ZjUFGT1 | 0.1979052 | 0.8731724 |
| Cyanidin | Zj.jz005267025 | ZjUFGT1 | 0.4597036 | 0.6959113 |
| Isorhamnetin O-hexoside | Zj.jz005267025 | ZjUFGT1 | 0.2490472 | 0.8397651 |
| Isorhamnetin O-rutinoside | Zj.jz005267025 | ZjUFGT1 | 0.5699488 | 0.613926 |
| Myricitrin | Zj.jz005267025 | ZjUFGT1 | -0.999755 | 0.0140862 |
| Narcissoside | Zj.jz005267025 | ZjUFGT1 | 0.7308165 | 0.478279 |
| Astragalin | Zj.jz005267025 | ZjUFGT1 | 0.4533493 | 0.700458 |
| Myricetin 3-O-galactoside | Zj.jz005267025 | ZjUFGT1 | 0.5582993 | 0.6229082 |
| Kaempferol 3-O-galactoside | Zj.jz005267025 | ZjUFGT1 | 0.4915359 | 0.6728713 |
| Kaempferitrin | Zj.jz005267025 | ZjUFGT1 | 0.9662259 | 0.165927 |
| Quercetin | Zj.jz005267025 | ZjUFGT1 | 0.3048737 | 0.8027715 |
| Kaempferol 3-O-β-rutinoside | Zj.jz005267025 | ZjUFGT1 | 0.4716028 | 0.6873513 |
| Quercetin O-rutinoside | Zj.jz005267025 | ZjUFGT1 | 0.8522912 | 0.3504249 |
| Quercetin 3-O-glucoside | Zj.jz005267025 | ZjUFGT1 | 0.5154986 | 0.6552135 |
| Quercetin 3-O-rutinoside | Zj.jz005267025 | ZjUFGT1 | 0.8110663 | 0.3977751 |
| Quercetin-3-β-O-galactoside | Zj.jz005267025 | ZjUFGT1 | 0.8966682 | 0.2919609 |
| Kaempferol 3-O-β-rutinoside | Zj.jz005267025 | ZjUFGT1 | 0.6918047 | 0.5136318 |
| Catechin | Zj.jz005267025 | ZjUFGT1 | 0.4765235 | 0.6837934 |
| Quercetin O-hexosyl-O-malonylhexoside | Zj.jz005267025 | ZjUFGT1 | 0.5909892 | 0.5974751 |
| Quercetin O-hexoside | Zj.jz005267025 | ZjUFGT1 | 0.5801718 | 0.6059708 |
| Quercetin-3-O-arabinofuranoside | Zj.jz005267025 | ZjUFGT1 | 0.8734198 | 0.3237943 |
| (+)-Gallocatechin (GC) | Zj.jz005267025 | ZjUFGT1 | 0.628767 | 0.5671198 |
| Epigallocatechin (EGC) | Zj.jz005267025 | ZjUFGT1 | 0.9771788 | 0.1362681 |
| L-Epicatechin | Zj.jz005267025 | ZjUFGT1 | 0.7962271 | 0.413652 |
| Naringenin | Zj.jz005267025 | ZjUFGT1 | -0.823849 | 0.3836443 |
| Caffeic acid | Zj.jz005267025 | ZjUFGT1 | -0.081767 | 0.9478871 |
| Ferulic acid | Zj.jz005267025 | ZjUFGT1 | -0.115363 | 0.9263938 |
| Procyanidin A1 | Zj.jz005267025 | ZjUFGT1 | 0.7999846 | 0.4096818 |
| Procyanidin B3 | Zj.jz005267025 | ZjUFGT1 | 0.7398279 | 0.4698138 |
| Procyanidin A3 | Zj.jz005267025 | ZjUFGT1 | 0.6851607 | 0.5194639 |
| Procyanidin B2 | Zj.jz005267025 | ZjUFGT1 | 0.9984425 | 0.0355353 |
| Procyanidin B1 | Zj.jz005267025 | ZjUFGT1 | 0.8911581 | 0.2997874 |
| Procyanidin A2 | Zj.jz005267025 | ZjUFGT1 | 0.7996915 | 0.4099927 |
| procyanidin A | Zj.jz005267025 | ZjUFGT1 | 0.8261596 | 0.3810419 |
| Procyanidin B | Zj.jz005267025 | ZjUFGT1 | 0.829236 | 0.3775518 |
| Dihydroquercetin | Zj.jz036025065 | ZjUFGT2 | 0.8456437 | 0.3584337 |
| Dihydrokaempferol | Zj.jz036025065 | ZjUFGT2 | -0.830525 | 0.3760806 |
| Cyanidin 3-O-glucoside | Zj.jz036025065 | ZjUFGT2 | -0.993136 | 0.0746344 |
| Delphinidin 3-O-glucoside | Zj.jz036025065 | ZjUFGT2 | 0.8189041 | 0.389164 |
| Cyanidin | Zj.jz036025065 | ZjUFGT2 | 0.9451129 | 0.2119029 |
| Isorhamnetin O-hexoside | Zj.jz036025065 | ZjUFGT2 | 0.8478806 | 0.3557567 |
| Isorhamnetin O-rutinoside | Zj.jz036025065 | ZjUFGT2 | 0.979249 | 0.1299176 |
| Myricitrin | Zj.jz036025065 | ZjUFGT2 | -0.70922 | 0.4980945 |
| Narcissoside | Zj.jz036025065 | ZjUFGT2 | 0.9999595 | 0.0057294 |
| Astragalin | Zj.jz036025065 | ZjUFGT2 | 0.9427552 | 0.2164497 |
| Myricetin 3-O-galactoside | Zj.jz036025065 | ZjUFGT2 | 0.9762923 | 0.1388998 |
| Kaempferol 3-O-galactoside | Zj.jz036025065 | ZjUFGT2 | 0.9563167 | 0.1888629 |
| Kaempferitrin | Zj.jz036025065 | ZjUFGT2 | 0.8777546 | 0.3180814 |
| Quercetin | Zj.jz036025065 | ZjUFGT2 | 0.877241 | 0.3187631 |
| Kaempferol 3-O-β-rutinoside | Zj.jz036025065 | ZjUFGT2 | 0.9494208 | 0.2033429 |
| Quercetin O-rutinoside | Zj.jz036025065 | ZjUFGT2 | 0.9780658 | 0.1335835 |
| Quercetin 3-O-glucoside | Zj.jz036025065 | ZjUFGT2 | 0.9640562 | 0.1712051 |
| Quercetin 3-O-rutinoside | Zj.jz036025065 | ZjUFGT2 | 0.99084 | 0.0862333 |
| Quercetin-3-β-O-galactoside | Zj.jz036025065 | ZjUFGT2 | 0.3446882 | 0.7759693 |
| Kaempferol 3-O-β-rutinoside | Zj.jz036025065 | ZjUFGT2 | 0.9989176 | 0.0296234 |
| Catechin | Zj.jz036025065 | ZjUFGT2 | 0.9511608 | 0.199785 |
| Quercetin O-hexosyl-O-malonylhexoside | Zj.jz036025065 | ZjUFGT2 | 0.9841585 | 0.1134668 |
| Quercetin O-hexoside | Zj.jz036025065 | ZjUFGT2 | 0.981705 | 0.1219624 |
| Quercetin-3-O-arabinofuranoside | Zj.jz036025065 | ZjUFGT2 | 0.9684995 | 0.160214 |
| (+)-Gallocatechin (GC) | Zj.jz036025065 | ZjUFGT2 | 0.9914903 | 0.0831115 |
| Epigallocatechin (EGC) | Zj.jz036025065 | ZjUFGT2 | 0.5617242 | 0.6202765 |
| L-Epicatechin | Zj.jz036025065 | ZjUFGT2 | 0.1600632 | 0.8976604 |
| Naringenin | Zj.jz036025065 | ZjUFGT2 | -0.206396 | 0.8676527 |
| Caffeic acid | Zj.jz036025065 | ZjUFGT2 | -0.746068 | 0.4638787 |
| Ferulic acid | Zj.jz036025065 | ZjUFGT2 | -0.768119 | 0.4423854 |
| Procyanidin A1 | Zj.jz036025065 | ZjUFGT2 | 0.166216 | 0.8936902 |
| Procyanidin B3 | Zj.jz036025065 | ZjUFGT2 | 0.9997514 | 0.0141945 |
| Procyanidin A3 | Zj.jz036025065 | ZjUFGT2 | 0.9984495 | 0.0354555 |
| Procyanidin B2 | Zj.jz036025065 | ZjUFGT2 | 0.6850694 | 0.5195437 |
| Procyanidin B1 | Zj.jz036025065 | ZjUFGT2 | 0.9584228 | 0.184221 |
| Procyanidin A2 | Zj.jz036025065 | ZjUFGT2 | 0.993249 | 0.0740157 |
| procyanidin A | Zj.jz036025065 | ZjUFGT2 | 0.9869487 | 0.1029665 |
| Procyanidin B | Zj.jz036025065 | ZjUFGT2 | 0.986051 | 0.1064565 |
| Dihydroquercetin | Zj.jz041429001 | ZjUFGT3 | -0.579608 | 0.6064115 |
| Dihydrokaempferol | Zj.jz041429001 | ZjUFGT3 | 0.9798228 | 0.1281028 |
| Cyanidin 3-O-glucoside | Zj.jz041429001 | ZjUFGT3 | 0.8743226 | 0.3226122 |
| Delphinidin 3-O-glucoside | Zj.jz041429001 | ZjUFGT3 | -0.539612 | 0.6371418 |
| Cyanidin | Zj.jz041429001 | ZjUFGT3 | -0.750235 | 0.4598807 |
| Isorhamnetin O-hexoside | Zj.jz041429001 | ZjUFGT3 | -0.583029 | 0.6037345 |
| Isorhamnetin O-rutinoside | Zj.jz041429001 | ZjUFGT3 | -0.828934 | 0.3778955 |
| Myricitrin | Zj.jz041429001 | ZjUFGT3 | 0.9238094 | 0.2501167 |
| Narcissoside | Zj.jz041429001 | ZjUFGT3 | -0.928471 | 0.2422484 |
| Astragalin | Zj.jz041429001 | ZjUFGT3 | -0.745494 | 0.4644275 |
| Myricetin 3-O-galactoside | Zj.jz041429001 | ZjUFGT3 | -0.82096 | 0.3868776 |
| Kaempferol 3-O-galactoside | Zj.jz041429001 | ZjUFGT3 | -0.773667 | 0.4368407 |
| Kaempferitrin | Zj.jz041429001 | ZjUFGT3 | -0.993943 | 0.0701036 |
| Quercetin | Zj.jz041429001 | ZjUFGT3 | -0.62923 | 0.5667409 |
| Kaempferol 3-O-β-rutinoside | Zj.jz041429001 | ZjUFGT3 | -0.759057 | 0.4513207 |
| Quercetin O-rutinoside | Zj.jz041429001 | ZjUFGT3 | -0.983899 | 0.1143943 |
| Quercetin 3-O-glucoside | Zj.jz041429001 | ZjUFGT3 | -0.790941 | 0.4191829 |
| Quercetin 3-O-rutinoside | Zj.jz041429001 | ZjUFGT3 | -0.967898 | 0.1617445 |
| Quercetin-3-β-O-galactoside | Zj.jz041429001 | ZjUFGT3 | -0.675343 | 0.5279915 |
| Kaempferol 3-O-β-rutinoside | Zj.jz041429001 | ZjUFGT3 | -0.906425 | 0.2776012 |
| Catechin | Zj.jz041429001 | ZjUFGT3 | -0.762683 | 0.4477629 |
| Quercetin O-hexosyl-O-malonylhexoside | Zj.jz041429001 | ZjUFGT3 | -0.84311 | 0.3614446 |
| Quercetin O-hexoside | Zj.jz041429001 | ZjUFGT3 | -0.835859 | 0.3699402 |
| Quercetin-3-O-arabinofuranoside | Zj.jz041429001 | ZjUFGT3 | -0.990512 | 0.0877638 |
| (+)-Gallocatechin (GC) | Zj.jz041429001 | ZjUFGT3 | -0.867783 | 0.3310893 |
| Epigallocatechin (EGC) | Zj.jz041429001 | ZjUFGT3 | -0.83382 | 0.3722987 |
| L-Epicatechin | Zj.jz041429001 | ZjUFGT3 | -0.522924 | 0.6496826 |
| Naringenin | Zj.jz041429001 | ZjUFGT3 | 0.5625057 | 0.6196749 |
| Caffeic acid | Zj.jz041429001 | ZjUFGT3 | 0.4373185 | 0.7118565 |
| Ferulic acid | Zj.jz041429001 | ZjUFGT3 | 0.4674256 | 0.6903632 |
| Procyanidin A1 | Zj.jz041429001 | ZjUFGT3 | -0.528229 | 0.6457124 |
| Procyanidin B3 | Zj.jz041429001 | ZjUFGT3 | -0.933327 | 0.2337833 |
| Procyanidin A3 | Zj.jz041429001 | ZjUFGT3 | -0.902518 | 0.2834333 |
| Procyanidin B2 | Zj.jz041429001 | ZjUFGT3 | -0.910388 | 0.2715659 |
| Procyanidin B1 | Zj.jz041429001 | ZjUFGT3 | -0.994989 | 0.0637568 |
| Procyanidin A2 | Zj.jz041429001 | ZjUFGT3 | -0.962896 | 0.1739621 |
| procyanidin A | Zj.jz041429001 | ZjUFGT3 | -0.974169 | 0.1450113 |
| Procyanidin B | Zj.jz041429001 | ZjUFGT3 | -0.975393 | 0.1415213 |
| Dihydroquercetin | Zj.jz041429001 | ZjUFGT4 | -0.613878 | 0.5792177 |
| Dihydrokaempferol | Zj.jz041429001 | ZjUFGT4 | -0.473103 | 0.6862679 |
| Cyanidin 3-O-glucoside | Zj.jz041429001 | ZjUFGT4 | 0.2135157 | 0.8630171 |
| Delphinidin 3-O-glucoside | Zj.jz041429001 | ZjUFGT4 | -0.651253 | 0.5484875 |
| Cyanidin | Zj.jz041429001 | ZjUFGT4 | -0.417592 | 0.7257486 |
| Isorhamnetin O-hexoside | Zj.jz041429001 | ZjUFGT4 | -0.610553 | 0.5818947 |
| Isorhamnetin O-rutinoside | Zj.jz041429001 | ZjUFGT4 | -0.297441 | 0.8077338 |
| Myricitrin | Zj.jz041429001 | ZjUFGT4 | -0.632261 | 0.564254 |
| Narcissoside | Zj.jz041429001 | ZjUFGT4 | -0.08882 | 0.9433808 |
| Astragalin | Zj.jz041429001 | ZjUFGT4 | -0.42407 | 0.7212018 |
| Myricetin 3-O-galactoside | Zj.jz041429001 | ZjUFGT4 | -0.310881 | 0.7987516 |
| Kaempferol 3-O-galactoside | Zj.jz041429001 | ZjUFGT4 | -0.384441 | 0.7487885 |
| Kaempferitrin | Zj.jz041429001 | ZjUFGT4 | 0.3909875 | 0.7442671 |
| Quercetin | Zj.jz041429001 | ZjUFGT4 | -0.563527 | 0.6188884 |
| Kaempferol 3-O-β-rutinoside | Zj.jz041429001 | ZjUFGT4 | -0.405337 | 0.7343086 |
| Quercetin O-rutinoside | Zj.jz041429001 | ZjUFGT4 | 0.1116622 | 0.9287651 |
| Quercetin 3-O-glucoside | Zj.jz041429001 | ZjUFGT4 | -0.358691 | 0.7664464 |
| Quercetin 3-O-rutinoside | Zj.jz041429001 | ZjUFGT4 | 0.0375093 | 0.9761152 |
| Quercetin-3-β-O-galactoside | Zj.jz041429001 | ZjUFGT4 | 0.9005152 | 0.2863792 |
| Kaempferol 3-O-β-rutinoside | Zj.jz041429001 | ZjUFGT4 | -0.143967 | 0.9080281 |
| Catechin | Zj.jz041429001 | ZjUFGT4 | -0.400221 | 0.7378664 |
| Quercetin O-hexosyl-O-malonylhexoside | Zj.jz041429001 | ZjUFGT4 | -0.272673 | 0.8241847 |
| Quercetin O-hexoside | Zj.jz041429001 | ZjUFGT4 | -0.285487 | 0.815689 |
| Quercetin-3-O-arabinofuranoside | Zj.jz041429001 | ZjUFGT4 | 0.153122 | 0.9021345 |
| (+)-Gallocatechin (GC) | Zj.jz041429001 | ZjUFGT4 | -0.226505 | 0.85454 |
| Epigallocatechin (EGC) | Zj.jz041429001 | ZjUFGT4 | 0.7684345 | 0.4420721 |
| L-Epicatechin | Zj.jz041429001 | ZjUFGT4 | 0.9667255 | 0.1646881 |
| Naringenin | Zj.jz041429001 | ZjUFGT4 | -0.953598 | 0.1946959 |
| Caffeic acid | Zj.jz041429001 | ZjUFGT4 | 0.7356297 | 0.4737728 |
| Ferulic acid | Zj.jz041429001 | ZjUFGT4 | 0.7123452 | 0.4952661 |
| Procyanidin A1 | Zj.jz041429001 | ZjUFGT4 | 0.9651114 | 0.1686583 |
| Procyanidin B3 | Zj.jz041429001 | ZjUFGT4 | -0.075568 | 0.951846 |
| Procyanidin A3 | Zj.jz041429001 | ZjUFGT4 | -0.153027 | 0.9021959 |
| Procyanidin B2 | Zj.jz041429001 | ZjUFGT4 | 0.6580006 | 0.5428048 |
| Procyanidin B1 | Zj.jz041429001 | ZjUFGT4 | 0.1902696 | 0.8781276 |
| Procyanidin A2 | Zj.jz041429001 | ZjUFGT4 | 0.0183257 | 0.9883329 |
| procyanidin A | Zj.jz041429001 | ZjUFGT4 | 0.0637593 | 0.959382 |
| Procyanidin B | Zj.jz041429001 | ZjUFGT4 | 0.0692293 | 0.955892 |
| Dihydroquercetin | Zj.jz018223048 | ZjUFGT5 | 0.9993582 | 0.0228101 |
| Dihydrokaempferol | Zj.jz018223048 | ZjUFGT5 | -0.372029 | 0.7573244 |
| Cyanidin 3-O-glucoside | Zj.jz018223048 | ZjUFGT5 | -0.886245 | 0.3066094 |
| Delphinidin 3-O-glucoside | Zj.jz018223048 | ZjUFGT5 | 0.9999226 | 0.0079202 |
| Cyanidin | Zj.jz018223048 | ZjUFGT5 | 0.9648301 | 0.1693409 |
| Isorhamnetin O-hexoside | Zj.jz018223048 | ZjUFGT5 | 0.9991987 | 0.0254871 |
| Isorhamnetin O-rutinoside | Zj.jz018223048 | ZjUFGT5 | 0.9230803 | 0.2513262 |
| Myricitrin | Zj.jz018223048 | ZjUFGT5 | -0.188402 | 0.8793384 |
| Narcissoside | Zj.jz018223048 | ZjUFGT5 | 0.8208743 | 0.3869732 |
| Astragalin | Zj.jz018223048 | ZjUFGT5 | 0.9666829 | 0.1647942 |
| Myricetin 3-O-galactoside | Zj.jz018223048 | ZjUFGT5 | 0.9284148 | 0.242344 |
| Kaempferol 3-O-galactoside | Zj.jz018223048 | ZjUFGT5 | 0.9546866 | 0.1923809 |
| Kaempferitrin | Zj.jz018223048 | ZjUFGT5 | 0.4549346 | 0.6993252 |
| Quercetin | Zj.jz018223048 | ZjUFGT5 | 0.9951877 | 0.0624808 |
| Kaempferol 3-O-β-rutinoside | Zj.jz018223048 | ZjUFGT5 | 0.9612084 | 0.1779009 |
| Quercetin O-rutinoside | Zj.jz018223048 | ZjUFGT5 | 0.6904475 | 0.5148273 |
| Quercetin 3-O-glucoside | Zj.jz018223048 | ZjUFGT5 | 0.9460656 | 0.2100387 |
| Quercetin 3-O-rutinoside | Zj.jz018223048 | ZjUFGT5 | 0.7422923 | 0.4674772 |
| Quercetin-3-β-O-galactoside | Zj.jz018223048 | ZjUFGT5 | -0.244447 | 0.8427869 |
| Kaempferol 3-O-β-rutinoside | Zj.jz018223048 | ZjUFGT5 | 0.8513075 | 0.3516204 |
| Catechin | Zj.jz018223048 | ZjUFGT5 | 0.9596519 | 0.1814588 |
| Quercetin O-hexosyl-O-malonylhexoside | Zj.jz018223048 | ZjUFGT5 | 0.9128346 | 0.2677771 |
| Quercetin O-hexoside | Zj.jz018223048 | ZjUFGT5 | 0.9182023 | 0.2592814 |
| Quercetin-3-O-arabinofuranoside | Zj.jz018223048 | ZjUFGT5 | 0.6595924 | 0.5414579 |
| (+)-Gallocatechin (GC) | Zj.jz018223048 | ZjUFGT5 | 0.8923345 | 0.2981324 |
| Epigallocatechin (EGC) | Zj.jz018223048 | ZjUFGT5 | -0.002388 | 0.9984797 |
| L-Epicatechin | Zj.jz018223048 | ZjUFGT5 | -0.424221 | 0.7210957 |
| Naringenin | Zj.jz018223048 | ZjUFGT5 | 0.3810815 | 0.7511035 |
| Caffeic acid | Zj.jz018223048 | ZjUFGT5 | -0.991587 | 0.0826348 |
| Ferulic acid | Zj.jz018223048 | ZjUFGT5 | -0.995392 | 0.0611415 |
| Procyanidin A1 | Zj.jz018223048 | ZjUFGT5 | -0.418566 | 0.7250659 |
| Procyanidin B3 | Zj.jz018223048 | ZjUFGT5 | 0.8132079 | 0.3954384 |
| Procyanidin A3 | Zj.jz018223048 | ZjUFGT5 | 0.8560782 | 0.3457883 |
| Procyanidin B2 | Zj.jz018223048 | ZjUFGT5 | 0.1552125 | 0.9007875 |
| Procyanidin B1 | Zj.jz018223048 | ZjUFGT5 | 0.6307864 | 0.5654648 |
| Procyanidin A2 | Zj.jz018223048 | ZjUFGT5 | 0.7550146 | 0.4552595 |
| procyanidin A | Zj.jz018223048 | ZjUFGT5 | 0.7244254 | 0.4842103 |
| Procyanidin B | Zj.jz018223048 | ZjUFGT5 | 0.7206354 | 0.4877004 |
| Dihydroquercetin | Zj.jz044759018 | ZjUFGT6 | 0.3536318 | 0.7698932 |
| Dihydrokaempferol | Zj.jz044759018 | ZjUFGT6 | -0.998456 | 0.0353788 |
| Cyanidin 3-O-glucoside | Zj.jz044759018 | ZjUFGT6 | -0.722383 | 0.4860938 |
| Delphinidin 3-O-glucoside | Zj.jz044759018 | ZjUFGT6 | 0.3080855 | 0.8006234 |
| Cyanidin | Zj.jz044759018 | ZjUFGT6 | 0.5577073 | 0.6233623 |
| Isorhamnetin O-hexoside | Zj.jz044759018 | ZjUFGT6 | 0.357562 | 0.7672162 |
| Isorhamnetin O-rutinoside | Zj.jz044759018 | ZjUFGT6 | 0.6596878 | 0.5413771 |
| Myricitrin | Zj.jz044759018 | ZjUFGT6 | -0.990755 | 0.0866351 |
| Narcissoside | Zj.jz044759018 | ZjUFGT6 | 0.8036938 | 0.4057301 |
| Astragalin | Zj.jz044759018 | ZjUFGT6 | 0.551765 | 0.6279091 |
| Myricetin 3-O-galactoside | Zj.jz044759018 | ZjUFGT6 | 0.6490188 | 0.5503592 |
| Kaempferol 3-O-galactoside | Zj.jz044759018 | ZjUFGT6 | 0.5873755 | 0.6003224 |
| Kaempferitrin | Zj.jz044759018 | ZjUFGT6 | 0.9892621 | 0.093378 |
| Quercetin | Zj.jz044759018 | ZjUFGT6 | 0.4111958 | 0.7302225 |
| Kaempferol 3-O-β-rutinoside | Zj.jz044759018 | ZjUFGT6 | 0.5688173 | 0.6148023 |
| Quercetin O-rutinoside | Zj.jz044759018 | ZjUFGT6 | 0.9062426 | 0.277876 |
| Quercetin 3-O-glucoside | Zj.jz044759018 | ZjUFGT6 | 0.6095946 | 0.5826645 |
| Quercetin 3-O-rutinoside | Zj.jz044759018 | ZjUFGT6 | 0.8723224 | 0.3252261 |
| Quercetin-3-β-O-galactoside | Zj.jz044759018 | ZjUFGT6 | 0.8405109 | 0.3645099 |
| Kaempferol 3-O-β-rutinoside | Zj.jz044759018 | ZjUFGT6 | 0.7694279 | 0.4410828 |
| Catechin | Zj.jz044759018 | ZjUFGT6 | 0.5734048 | 0.6112445 |
| Quercetin O-hexosyl-O-malonylhexoside | Zj.jz044759018 | ZjUFGT6 | 0.6788859 | 0.5249262 |
| Quercetin O-hexoside | Zj.jz044759018 | ZjUFGT6 | 0.6690273 | 0.5334218 |
| Quercetin-3-O-arabinofuranoside | Zj.jz044759018 | ZjUFGT6 | 0.9231291 | 0.2512454 |
| (+)-Gallocatechin (GC) | Zj.jz044759018 | ZjUFGT6 | 0.7131112 | 0.4945709 |
| Epigallocatechin (EGC) | Zj.jz044759018 | ZjUFGT6 | 0.9466856 | 0.208817 |
| L-Epicatechin | Zj.jz044759018 | ZjUFGT6 | 0.7222663 | 0.486201 |
| Naringenin | Zj.jz044759018 | ZjUFGT6 | -0.754052 | 0.4561933 |
| Caffeic acid | Zj.jz044759018 | ZjUFGT6 | -0.194569 | 0.8753381 |
| Ferulic acid | Zj.jz044759018 | ZjUFGT6 | -0.227569 | 0.8538448 |
| Procyanidin A1 | Zj.jz044759018 | ZjUFGT6 | 0.7265654 | 0.4822308 |
| Procyanidin B3 | Zj.jz044759018 | ZjUFGT6 | 0.8115348 | 0.3972649 |
| Procyanidin A3 | Zj.jz044759018 | ZjUFGT6 | 0.7635442 | 0.4469149 |
| Procyanidin B2 | Zj.jz044759018 | ZjUFGT6 | 0.9856222 | 0.1080843 |
| Procyanidin B1 | Zj.jz044759018 | ZjUFGT6 | 0.9369685 | 0.2272384 |
| Procyanidin A2 | Zj.jz044759018 | ZjUFGT6 | 0.8627791 | 0.3374438 |
| procyanidin A | Zj.jz044759018 | ZjUFGT6 | 0.8848709 | 0.3084929 |
| Procyanidin B | Zj.jz044759018 | ZjUFGT6 | 0.8874114 | 0.3050029 |
| Dihydroquercetin | Zj.jz044759027 | ZjUFGT7 | 0.911559 | 0.269759 |
| Dihydrokaempferol | Zj.jz044759027 | ZjUFGT7 | -0.74515 | 0.4647554 |
| Cyanidin 3-O-glucoside | Zj.jz044759027 | ZjUFGT7 | -0.999757 | 0.0140404 |
| Delphinidin 3-O-glucoside | Zj.jz044759027 | ZjUFGT7 | 0.8906574 | 0.3004892 |
| Cyanidin | Zj.jz044759027 | ZjUFGT7 | 0.9813245 | 0.1232281 |
| Isorhamnetin O-hexoside | Zj.jz044759027 | ZjUFGT7 | 0.9132799 | 0.267082 |
| Isorhamnetin O-rutinoside | Zj.jz044759027 | ZjUFGT7 | 0.9979022 | 0.0412429 |
| Myricitrin | Zj.jz044759027 | ZjUFGT7 | -0.604471 | 0.5867693 |
| Narcissoside | Zj.jz044759027 | ZjUFGT7 | 0.9890252 | 0.0944041 |
| Astragalin | Zj.jz044759027 | ZjUFGT7 | 0.9799256 | 0.1277749 |
| Myricetin 3-O-galactoside | Zj.jz044759027 | ZjUFGT7 | 0.9968895 | 0.0502251 |
| Kaempferol 3-O-galactoside | Zj.jz044759027 | ZjUFGT7 | 0.9876421 | 0.1001882 |
| Kaempferitrin | Zj.jz044759027 | ZjUFGT7 | 0.8027336 | 0.4067562 |
| Quercetin | Zj.jz044759027 | ZjUFGT7 | 0.935395 | 0.2300883 |
| Kaempferol 3-O-β-rutinoside | Zj.jz044759027 | ZjUFGT7 | 0.9838222 | 0.1146681 |
| Quercetin O-rutinoside | Zj.jz044759027 | ZjUFGT7 | 0.9396733 | 0.2222582 |
| Quercetin 3-O-glucoside | Zj.jz044759027 | ZjUFGT7 | 0.9916087 | 0.0825303 |
| Quercetin 3-O-rutinoside | Zj.jz044759027 | ZjUFGT7 | 0.9624944 | 0.1749081 |
| Quercetin-3-β-O-galactoside | Zj.jz044759027 | ZjUFGT7 | 0.2110183 | 0.8646441 |
| Kaempferol 3-O-β-rutinoside | Zj.jz044759027 | ZjUFGT7 | 0.9957011 | 0.0590514 |
| Catechin | Zj.jz044759027 | ZjUFGT7 | 0.984808 | 0.1111103 |
| Quercetin O-hexosyl-O-malonylhexoside | Zj.jz044759027 | ZjUFGT7 | 0.9992418 | 0.024792 |
| Quercetin O-hexoside | Zj.jz044759027 | ZjUFGT7 | 0.9986333 | 0.0332877 |
| Quercetin-3-O-arabinofuranoside | Zj.jz044759027 | ZjUFGT7 | 0.9245461 | 0.2488888 |
| (+)-Gallocatechin (GC) | Zj.jz044759027 | ZjUFGT7 | 0.9999618 | 0.0055633 |
| Epigallocatechin (EGC) | Zj.jz044759027 | ZjUFGT7 | 0.441418 | 0.7089512 |
| L-Epicatechin | Zj.jz044759027 | ZjUFGT7 | 0.021463 | 0.9863352 |
| Naringenin | Zj.jz044759027 | ZjUFGT7 | -0.068547 | 0.9563275 |
| Caffeic acid | Zj.jz044759027 | ZjUFGT7 | -0.831292 | 0.3752039 |
| Ferulic acid | Zj.jz044759027 | ZjUFGT7 | -0.84958 | 0.3537106 |
| Procyanidin A1 | Zj.jz044759027 | ZjUFGT7 | 0.0276975 | 0.982365 |
| Procyanidin B3 | Zj.jz044759027 | ZjUFGT7 | 0.9869732 | 0.1028693 |
| Procyanidin A3 | Zj.jz044759027 | ZjUFGT7 | 0.9965078 | 0.0532192 |
| Procyanidin B2 | Zj.jz044759027 | ZjUFGT7 | 0.5772925 | 0.6082185 |
| Procyanidin B1 | Zj.jz044759027 | ZjUFGT7 | 0.909522 | 0.2728957 |
| Procyanidin A2 | Zj.jz044759027 | ZjUFGT7 | 0.9675235 | 0.1626904 |
| procyanidin A | Zj.jz044759027 | ZjUFGT7 | 0.9550318 | 0.1916413 |
| Procyanidin B | Zj.jz044759027 | ZjUFGT7 | 0.953392 | 0.1951313 |
