# Supplementary material for: Association of Bitter Metabolites and Flavonoid Synthesis Pathway in Jujube Fruit
Source: Front Nutr. 2022 May 31;9:901756. doi: 10.3389/fnut.2022.901756 (PMC9194943; doi:10.3389/fnut.2022.901756)
Supplement: Supplementary file 6 [file Table_6.docx]

**Table S6**

Differential flavonoid metabolites (DFMs) during ‘Junzao’ jujube fruit skins development.

| **DFMs in DAP90 vs.DAP30** |  |  |  |  |  |  |  |  |  |  |  |
| --- | --- | --- | --- | --- | --- | --- | --- | --- | --- | --- | --- |
| Compounds | Class | DAP90-1 | DAP90-2 | DAP90-3 | DAP30-1 | DAP30-2 | DAP30-3 | VIP | Fold_Change | LogFC | type |
| Caffeoyl shikimic acid | Phenylpropanoids | 4.72E+03 | 7.06E+03 | 5.23E+03 | 4.06E+02 | 3.94E+02 | 8.95E+02 | 1.46E+00 | 9.96E-02 | -3.33E+00 | up |
| Cinnamic acid | Phenylpropanoids | 3.01E+04 | 3.07E+04 | 3.35E+04 | 4.26E+02 | 1.00E+03 | 2.02E+03 | 1.78E+00 | 3.65E-02 | -4.77E+00 | up |
| p-Coumaric acid | Phenylpropanoids | 1.05E+04 | 7.50E+03 | 9.22E+03 | 2.89E+04 | 3.81E+04 | 2.88E+04 | 1.07E+00 | 3.52E+00 | 1.82E+00 | down |
| Ferulic acid O-hexoside | Phenylpropanoids | 2.26E+04 | 3.80E+04 | 2.25E+04 | 3.30E+03 | 3.95E+03 | 3.20E+03 | 1.37E+00 | 1.26E-01 | -2.99E+00 | up |
| Dihydroquercetin (Taxifolin) | Dihydroflavonol | 9.00E+00 | 9.00E+00 | 9.00E+00 | 2.18E+05 | 1.81E+05 | 1.55E+05 | 3.04E+00 | 2.05E+04 | 1.43E+01 | down |
| Cyanidin 3-O-malonylhexoside | Anthocyanins | 9.00E+00 | 5.19E+04 | 1.84E+04 | 9.00E+00 | 9.00E+00 | 9.00E+00 | 1.90E+00 | 3.84E-04 | -1.13E+01 | up |
| Petunidin 3-O-glucoside | Anthocyanins | 2.95E+06 | 4.07E+06 | 6.29E+06 | 9.00E+00 | 9.00E+00 | 3.37E+04 | 2.91E+00 | 2.53E-03 | -8.62E+00 | up |
| Delphinidin 3-Glu | Anthocyanins | 1.02E+06 | 4.83E+05 | 5.61E+05 | 3.36E+06 | 2.53E+06 | 2.76E+06 | 1.15E+00 | 4.19E+00 | 2.07E+00 | down |
| Chrysoeriol 7-O-hexoside | Flavone | 9.45E+04 | 6.74E+04 | 6.03E+04 | 2.35E+05 | 2.42E+05 | 2.02E+05 | 1.01E+00 | 3.06E+00 | 1.61E+00 | down |
| Tricetin | Flavone | 7.30E+03 | 5.12E+03 | 5.58E+03 | 2.68E+04 | 3.75E+04 | 2.62E+04 | 1.21E+00 | 5.03E+00 | 2.33E+00 | down |
| 5,3'-Dihydroxyflavone | Flavone | 5.73E+02 | 7.56E+02 | 1.49E+03 | 1.58E+04 | 1.78E+04 | 1.74E+04 | 1.65E+00 | 1.81E+01 | 4.18E+00 | down |
| 5-Methoxyflavanone | Flavone | 1.47E+04 | 1.41E+04 | 1.55E+04 | 2.75E+03 | 2.97E+03 | 2.34E+03 | 1.26E+00 | 1.82E-01 | -2.46E+00 | up |
| Isorhamnetin O-hexoside | Flavonol | 6.03E+04 | 6.12E+04 | 7.70E+04 | 2.26E+05 | 1.98E+05 | 2.58E+05 | 1.06E+00 | 3.44E+00 | 1.78E+00 | down |
| Astragalin | Flavonol | 1.66E+06 | 1.35E+06 | 1.39E+06 | 7.13E+06 | 5.53E+06 | 3.42E+06 | 1.05E+00 | 3.65E+00 | 1.87E+00 | down |
| Quercetin-3-O-arabinofuranoside (Avicularin) | Flavonol | 5.00E+06 | 2.33E+06 | 3.11E+06 | 1.53E+07 | 1.18E+07 | 9.29E+06 | 1.05E+00 | 3.49E+00 | 1.80E+00 | down |
| Quercetin O-hexosyl-O-malonylhexoside | Flavonol | 3.62E+03 | 3.77E+03 | 3.73E+03 | 1.10E+04 | 1.17E+04 | 1.36E+04 | 1.04E+00 | 3.26E+00 | 1.71E+00 | down |
| Quercetin | Flavonol | 1.37E+06 | 8.41E+05 | 9.67E+05 | 2.21E+07 | 2.21E+07 | 1.71E+07 | 1.15E+00 | 2.23E+00 | 1.95E+00 | down |
| Protocatechuic acid | Flavanol | 6.17E+04 | 9.00E+00 | 4.92E+04 | 9.00E+00 | 9.00E+00 | 9.00E+00 | 1.95E+00 | 2.43E-04 | -1.20E+01 | up |
| Protocatechuic aldehyde | Flavanol | 9.00E+00 | 3.87E+06 | 2.95E+06 | 7.11E+06 | 7.49E+06 | 8.40E+06 | 1.55E+00 | 3.37E+00 | 1.75E+00 | down |
| Catechin | Flavanol | 1.22E+06 | 1.20E+06 | 9.62E+05 | 3.52E+06 | 3.49E+06 | 3.52E+06 | 1.03E+00 | 3.11E+00 | 1.64E+00 | down |
| Pinobanksin | Flavanone | 4.06E+05 | 1.85E+05 | 2.61E+05 | 1.55E+06 | 1.54E+06 | 1.50E+06 | 1.25E+00 | 5.39E+00 | 2.43E+00 | down |
| Butin | Flavanone | 3.65E+05 | 3.11E+05 | 3.28E+05 | 1.70E+06 | 1.92E+06 | 2.30E+06 | 1.28E+00 | 5.90E+00 | 2.56E+00 | down |
| Neohesperidin | Flavanone | 8.91E+04 | 8.44E+04 | 5.52E+04 | 3.18E+05 | 3.55E+05 | 3.30E+05 | 1.17E+00 | 4.39E+00 | 2.13E+00 | down |
| Hesperidin | Flavanone | 2.64E+04 | 4.69E+04 | 1.66E+04 | 1.83E+05 | 1.33E+05 | 1.33E+05 | 1.21E+00 | 4.99E+00 | 2.32E+00 | down |
| Naringenin | Flavanone | 4.43E+03 | 6.76E+03 | 4.96E+03 | 2.49E+04 | 2.49E+04 | 2.87E+04 | 1.21E+00 | 4.86E+00 | 2.28E+00 | down |
| 2-Methoxybenzoic acid | Hydroxycinnamoyl derivatives | 2.20E+05 | 1.33E+05 | 9.00E+00 | 7.88E+05 | 5.97E+05 | 9.06E+05 | 1.62E+00 | 6.49E+00 | 2.70E+00 | down |
| Hydrocinnamic acid | Hydroxycinnamoyl derivatives | 9.35E+04 | 1.70E+05 | 2.25E+05 | 9.00E+00 | 9.00E+00 | 9.00E+00 | 3.01E+00 | 5.53E-05 | -1.41E+01 | up |
| Syringin | Hydroxycinnamoyl derivatives | 9.00E+00 | 7.79E+04 | 9.00E+00 | 8.97E+04 | 9.27E+04 | 5.18E+04 | 1.98E+00 | 3.01E+00 | 1.59E+00 | down |
| p-Coumaraldehyde | Hydroxycinnamoyl derivatives | 2.40E+06 | 2.45E+06 | 4.93E+06 | 9.00E+00 | 4.82E+04 | 9.00E+00 | 2.83E+00 | 4.93E-03 | -7.66E+00 | up |
| 1-O-beta-D-Glucopyranosyl sinapate | Hydroxycinnamoyl derivatives | 3.37E+05 | 6.78E+05 | 5.60E+05 | 1.10E+05 | 1.08E+05 | 8.69E+04 | 1.20E+00 | 1.94E-01 | -2.37E+00 | up |
| Caffeic acid | Hydroxycinnamoyl derivatives | 2.35E+05 | 2.79E+05 | 3.96E+05 | 3.27E+04 | 4.51E+04 | 2.45E+04 | 1.41E+00 | 1.12E-01 | -3.15E+00 | up |
| Ferulic acid | Hydroxycinnamoyl derivatives | 2.89E+05 | 2.70E+05 | 3.04E+05 | 5.92E+04 | 3.43E+04 | 6.23E+04 | 1.26E+00 | 1.81E-01 | -2.47E+00 | up |
| trans-4-Hydroxycinnamic Acid Methyl Ester | Hydroxycinnamoyl derivatives | 6.33E+05 | 4.36E+05 | 8.24E+05 | 3.10E+06 | 2.24E+06 | 2.75E+06 | 1.15E+00 | 4.27E+00 | 2.10E+00 | down |
| Methyl p-coumarate | Hydroxycinnamoyl derivatives | 1.51E+06 | 9.39E+05 | 1.77E+06 | 7.47E+06 | 5.70E+06 | 6.34E+06 | 1.19E+00 | 4.62E+00 | 2.21E+00 | down |
| O-Feruloyl hydroxylcoumarin | Coumarins | 9.61E+05 | 4.12E+05 | 8.48E+05 | 9.00E+00 | 9.00E+00 | 9.00E+00 | 3.24E+00 | 1.22E-05 | -1.63E+01 | up |
| Scopoletin (7-Hydroxy-5-methoxycoumarin) | Coumarins | 1.21E+06 | 5.16E+05 | 2.70E+06 | 9.00E+00 | 9.00E+00 | 9.00E+00 | 3.31E+00 | 6.10E-06 | -1.73E+01 | up |
| Esculin (6,7-Dihydroxycoumarin-6-glucoside) | Coumarins | 1.12E+07 | 3.99E+06 | 7.39E+06 | 1.32E+06 | 8.51E+05 | 1.48E+06 | 1.23E+00 | 1.62E-01 | -2.63E+00 | up |
| Procyanidin A1 | Proanthocyanidins | 2.32E+04 | 2.67E+04 | 2.14E+04 | 9.00E+00 | 9.00E+00 | 1.20E+04 | 1.96E+00 | 1.69E-01 | -2.57E+00 | up |
| **DFMs in DAP110 vs.DAP30** |  |  |  |  |  |  |  |  |  |  |  |
| Compounds | Class | DAP110-1 | DAP110-2 | DAP110-3 | DAP30-1 | DAP30-2 | DAP30-3 | VIP | Fold_Change | LogFC | type |
| Caffeoyl shikimic acid | Phenylpropanoids | 5.86E+04 | 7.71E+04 | 5.22E+04 | 4.06E+02 | 3.94E+02 | 8.95E+02 | 1.62E+00 | 9.02E-03 | -6.79E+00 | up |
| Cinnamic acid | Phenylpropanoids | 1.90E+04 | 1.95E+04 | 1.61E+04 | 4.26E+02 | 1.00E+03 | 2.02E+03 | 1.25E+00 | 6.31E-02 | -3.99E+00 | up |
| Ferulic acid O-hexoside | Phenylpropanoids | 2.18E+04 | 3.44E+04 | 2.20E+04 | 3.30E+03 | 3.95E+03 | 3.20E+03 | 1.05E+00 | 1.34E-01 | -2.90E+00 | up |
| Benzoic acid | Benzoic acid derivatives | 1.45E+07 | 1.61E+07 | 1.37E+07 | 5.73E+05 | 1.28E+06 | 3.80E+05 | 1.30E+00 | 5.04E-02 | -4.31E+00 | up |
| Dihydroquercetin (Taxifolin) | Dihydroflavonol | 9.00E+00 | 1.90E+04 | 9.00E+00 | 2.18E+05 | 1.81E+05 | 1.55E+05 | 1.83E+00 | 2.91E+01 | 4.86E+00 | down |
| Cyanidin 3-O-malonylhexoside | Anthocyanins | 5.27E+04 | 2.03E+04 | 9.00E+00 | 9.00E+00 | 9.00E+00 | 9.00E+00 | 1.46E+00 | 3.70E-04 | -1.14E+01 | up |
| Petunidin 3-O-glucoside | Anthocyanins | 4.10E+06 | 4.85E+06 | 4.49E+06 | 9.00E+00 | 9.00E+00 | 3.37E+04 | 2.26E+00 | 2.51E-03 | -8.64E+00 | up |
| Cyanidin-3,5-O-diglucoside (Cyanin) | Anthocyanin | 1.39E+03 | 1.58E+03 | 9.53E+02 | 1.33E+04 | 1.27E+04 | 1.22E+04 | 1.12E+00 | 9.74E+00 | 3.28E+00 | down |
| Luteolin 7-O-glucoside (Cynaroside) | Flavone | 6.51E+05 | 3.70E+05 | 3.43E+05 | 2.82E+06 | 3.41E+06 | 4.10E+06 | 1.06E+00 | 7.57E+00 | 2.92E+00 | down |
| Chrysoeriol 7-O-hexoside | Flavone | 3.84E+04 | 1.86E+04 | 1.82E+04 | 2.35E+05 | 2.42E+05 | 2.02E+05 | 1.11E+00 | 9.03E+00 | 3.17E+00 | down |
| Luteolin-3',7-di-O-glucoside | Flavone | 4.59E+03 | 2.93E+03 | 3.89E+03 | 2.38E+04 | 2.51E+04 | 3.12E+04 | 1.04E+00 | 7.02E+00 | 2.81E+00 | down |
| Tricetin | Flavone | 1.07E+03 | 3.42E+03 | 2.77E+03 | 2.68E+04 | 3.75E+04 | 2.62E+04 | 1.18E+00 | 1.25E+01 | 3.64E+00 | down |
| Tricetin O-malonylhexoside | Flavone | 1.10E+03 | 1.42E+03 | 1.55E+03 | 1.52E+04 | 1.71E+04 | 1.81E+04 | 1.18E+00 | 1.24E+01 | 3.63E+00 | down |
| Tricetin O-hexoside | Flavone | 5.04E+05 | 1.01E+06 | 4.94E+05 | 8.33E+06 | 8.83E+06 | 8.09E+06 | 1.19E+00 | 1.26E+01 | 3.65E+00 | down |
| Apigenin 7-O-rutinoside | Flavone | 9.05E+03 | 9.91E+03 | 2.23E+03 | 1.62E+05 | 1.55E+05 | 1.78E+05 | 1.33E+00 | 2.34E+01 | 4.55E+00 | down |
| Apigenin 7-O-neohesperidoside (Rhoifolin) | Flavone | 3.00E+03 | 9.06E+02 | 1.41E+03 | 3.69E+04 | 4.34E+04 | 3.39E+04 | 1.31E+00 | 2.15E+01 | 4.43E+00 | down |
| Luteolin O-sinapoylhexoside | Flavone | 1.56E+03 | 2.90E+03 | 1.50E+03 | 1.13E+04 | 1.40E+04 | 1.89E+04 | 1.04E+00 | 7.42E+00 | 2.89E+00 | down |
| Acacetin O-Glucuronic acid | Flavone | 2.32E+03 | 1.44E+03 | 1.55E+03 | 1.01E+04 | 1.09E+04 | 1.43E+04 | 1.02E+00 | 6.65E+00 | 2.73E+00 | down |
| 5,3'-Dihydroxyflavone | Flavone | 5.40E+02 | 1.06E+03 | 1.70E+03 | 1.58E+04 | 1.78E+04 | 1.74E+04 | 1.24E+00 | 1.55E+01 | 3.95E+00 | down |
| 3,4',5,7-tetrahydroxy-3'-methoxyflavone | Flavone | 5.61E+03 | 4.79E+03 | 8.27E+03 | 4.78E+04 | 4.19E+04 | 4.58E+04 | 1.05E+00 | 7.26E+00 | 2.86E+00 | down |
| Astragalin | Flavonol | 4.54E+05 | 5.43E+05 | 4.35E+05 | 7.13E+06 | 5.53E+06 | 3.42E+06 | 1.14E+00 | 1.12E+01 | 3.49E+00 | down |
| Myricetin 3-O-galactoside | Flavonol | 1.37E+05 | 8.09E+04 | 1.23E+05 | 2.53E+06 | 1.81E+06 | 1.83E+06 | 1.27E+00 | 1.81E+01 | 4.18E+00 | down |
| Quercetin-3-O-arabinofuranoside (Avicularin) | Flavonol | 1.11E+06 | 9.04E+05 | 1.73E+06 | 1.53E+07 | 1.18E+07 | 9.29E+06 | 1.12E+00 | 9.72E+00 | 3.28E+00 | down |
| Quercetin | Flavonol | 3.09E+05 | 2.28E+05 | 4.44E+05 | 2.21E+07 | 2.21E+07 | 1.71E+07 | 1.00E+00 | 6.25E+00 | 2.64E+00 | down |
| Kaempferol 3-O-glucoside-2'-O-rhamnoside | Flavonol | 4.33E+04 | 4.96E+04 | 4.40E+04 | 2.82E+05 | 2.81E+05 | 3.02E+05 | 1.01E+00 | 6.32E+00 | 2.66E+00 | down |
| Quercetin 3-O-a-L-rhamnoside (Quercitrin) | Flavonol | 4.62E+04 | 5.80E+04 | 9.51E+04 | 4.59E+05 | 3.99E+05 | 4.28E+05 | 1.02E+00 | 6.45E+00 | 2.69E+00 | down |
| Kaempferitrin | Flavonol | 3.54E+04 | 3.09E+04 | 3.05E+04 | 5.62E+05 | 4.89E+05 | 4.69E+05 | 1.24E+00 | 1.57E+01 | 3.97E+00 | down |
| Quercetin O-hexoside | Flavonol | 3.71E+06 | 7.53E+06 | 4.32E+06 | 3.29E+07 | 3.69E+07 | 3.42E+07 | 1.03E+00 | 6.68E+00 | 2.74E+00 | down |
| Protocatechuic acid | Flavanol | 1.44E+05 | 2.33E+05 | 2.34E+05 | 9.00E+00 | 9.00E+00 | 9.00E+00 | 2.36E+00 | 4.42E-05 | -1.45E+01 | up |
| Protocatechuic aldehyde | Flavanol | 1.55E+06 | 9.00E+00 | 9.00E+00 | 7.11E+06 | 7.49E+06 | 8.40E+06 | 2.03E+00 | 1.48E+01 | 3.89E+00 | down |
| Catechin | Flavanol | 4.39E+05 | 3.27E+05 | 5.08E+05 | 3.52E+06 | 3.49E+06 | 3.52E+06 | 1.08E+00 | 8.27E+00 | 3.05E+00 | down |
| (+)-Gallocatechin (GC) | Flavanol | 1.33E+06 | 1.32E+06 | 8.92E+05 | 1.25E+07 | 1.23E+07 | 1.19E+07 | 1.14E+00 | 1.04E+01 | 3.37E+00 | down |
| Catechin-catechin-catechin | Flavanol | 1.63E+06 | 8.79E+05 | 5.56E+05 | 1.29E+07 | 1.33E+07 | 1.58E+07 | 1.21E+00 | 1.37E+01 | 3.78E+00 | down |
| Prodelphinidin B dimer (Epi)gallocatechin–(epi)gallocatechin | Flavanol | 4.33E+04 | 5.05E+04 | 3.79E+04 | 2.70E+05 | 3.79E+05 | 2.93E+05 | 1.04E+00 | 7.15E+00 | 2.84E+00 | down |
| Butin | Flavanone | 5.61E+04 | 1.00E+05 | 7.93E+04 | 1.70E+06 | 1.92E+06 | 2.30E+06 | 1.34E+00 | 2.51E+01 | 4.65E+00 | down |
| Naringenin 7-O-glucoside | Flavanone | 4.65E+03 | 7.82E+03 | 9.21E+03 | 6.80E+04 | 8.21E+04 | 6.88E+04 | 1.13E+00 | 1.01E+01 | 3.34E+00 | down |
| Neohesperidin | Flavanone | 1.77E+04 | 2.03E+04 | 2.45E+04 | 3.18E+05 | 3.55E+05 | 3.30E+05 | 1.24E+00 | 1.60E+01 | 4.00E+00 | down |
| Hesperidin | Flavanone | 6.88E+03 | 1.77E+04 | 1.14E+04 | 1.83E+05 | 1.33E+05 | 1.33E+05 | 1.18E+00 | 1.25E+01 | 3.64E+00 | down |
| Hesperetin 5-O-glucoside | Flavanone | 2.05E+03 | 7.61E+03 | 2.92E+03 | 1.35E+05 | 1.95E+05 | 1.84E+05 | 1.45E+00 | 4.09E+01 | 5.35E+00 | down |
| Prunin | Flavanone | 1.46E+03 | 1.64E+03 | 1.43E+03 | 1.34E+04 | 1.89E+04 | 2.01E+04 | 1.16E+00 | 1.16E+01 | 3.53E+00 | down |
| 2-Methoxybenzoic acid | Hydroxycinnamoyl derivatives | 9.00E+00 | 9.00E+00 | 9.00E+00 | 7.88E+05 | 5.97E+05 | 9.06E+05 | 2.51E+00 | 8.49E+04 | 1.64E+01 | down |
| Hydrocinnamic acid | Hydroxycinnamoyl derivatives | 9.98E+05 | 5.96E+05 | 8.33E+05 | 9.00E+00 | 9.00E+00 | 9.00E+00 | 2.51E+00 | 1.11E-05 | -1.65E+01 | up |
| p-Coumaraldehyde | Hydroxycinnamoyl derivatives | 2.20E+06 | 2.87E+06 | 4.48E+06 | 9.00E+00 | 4.82E+04 | 9.00E+00 | 2.18E+00 | 5.05E-03 | -7.63E+00 | up |
| 1-O-beta-D-Glucopyranosyl sinapate | Hydroxycinnamoyl derivatives | 1.24E+06 | 4.04E+06 | 2.02E+06 | 1.10E+05 | 1.08E+05 | 8.69E+04 | 1.29E+00 | 4.18E-02 | -4.58E+00 | up |
| Caffeic acid | Hydroxycinnamoyl derivatives | 1.68E+05 | 2.20E+05 | 3.56E+05 | 3.27E+04 | 4.51E+04 | 2.45E+04 | 1.02E+00 | 1.38E-01 | -2.86E+00 | up |
| trans-4-Hydroxycinnamic Acid Methyl Ester | Hydroxycinnamoyl derivatives | 2.23E+05 | 1.99E+05 | 1.76E+05 | 3.10E+06 | 2.24E+06 | 2.75E+06 | 1.20E+00 | 1.35E+01 | 3.76E+00 | down |
| Methyl p-coumarate | Hydroxycinnamoyl derivatives | 5.37E+05 | 4.73E+05 | 5.72E+05 | 7.47E+06 | 5.70E+06 | 6.34E+06 | 1.18E+00 | 1.23E+01 | 3.62E+00 | down |
| O-Feruloyl hydroxylcoumarin | Coumarins | 1.12E+06 | 7.05E+05 | 9.00E+00 | 9.00E+00 | 9.00E+00 | 9.00E+00 | 1.74E+00 | 1.48E-05 | -1.60E+01 | up |
| Scopoletin (7-Hydroxy-5-methoxycoumarin) | Coumarins | 1.59E+06 | 1.44E+06 | 1.61E+06 | 9.00E+00 | 9.00E+00 | 9.00E+00 | 2.59E+00 | 5.82E-06 | -1.74E+01 | up |
| Esculin (6,7-Dihydroxycoumarin-6-glucoside) | Coumarins | 8.90E+06 | 1.13E+07 | 6.35E+06 | 1.32E+06 | 8.51E+05 | 1.48E+06 | 1.04E+00 | 1.38E-01 | -2.86E+00 | up |
| Procyanidin B1 | Proanthocyanidins | 7.85E+05 | 8.22E+05 | 7.97E+05 | 1.47E+07 | 1.50E+07 | 1.64E+07 | 1.28E+00 | 1.92E+01 | 4.26E+00 | down |
| procyanidin A | Proanthocyanidins | 6.16E+04 | 5.78E+04 | 6.50E+04 | 1.68E+06 | 1.93E+06 | 1.91E+06 | 1.37E+00 | 2.99E+01 | 4.90E+00 | down |
| Procyanidin B | Proanthocyanidins | 4.83E+05 | 4.71E+05 | 5.16E+05 | 7.99E+06 | 8.93E+06 | 8.77E+06 | 1.26E+00 | 1.75E+01 | 4.13E+00 | down |
|  |  |  |  |  |  |  |  |  |  |  |  |
| **DFMs in DAP110 vs.DAP90** |  |  |  |  |  |  |  |  |  |  |  |
| Compounds | Class | DAP110-1 | DAP110-2 | DAP110-3 | DAP90-1 | DAP90-2 | DAP90-3 | VIP | Fold_Change | LogFC | type |
| trans-Cinnamaldehyde | Phenylpropanoids | 8.15E+04 | 4.80E+04 | 7.48E+04 | 2.12E+05 | 2.27E+05 | 1.76E+05 | 1.04E+00 | 3.01E+00 | 1.59E+00 | down |
| Caffeoyl shikimic acid | Phenylpropanoids | 5.86E+04 | 7.71E+04 | 5.22E+04 | 4.72E+03 | 7.06E+03 | 5.23E+03 | 1.56E+00 | 9.05E-02 | -3.47E+00 | up |
| Benzamidine | Phenylpropanoids | 5.14E+05 | 5.46E+05 | 5.14E+05 | 5.69E+04 | 5.64E+04 | 6.17E+04 | 1.49E+00 | 1.11E-01 | -3.17E+00 | up |
| 2,4-Dihydroxybenzoic acid | Benzoic acid derivatives | 1.64E+06 | 2.41E+06 | 2.03E+06 | 5.17E+05 | 4.42E+05 | 5.06E+05 | 1.19E+00 | 2.41E-01 | -2.05E+00 | up |
| Benzoic acid | Benzoic acid derivatives | 1.45E+07 | 1.61E+07 | 1.37E+07 | 1.02E+06 | 2.18E+06 | 2.62E+06 | 1.44E+00 | 1.31E-01 | -2.93E+00 | up |
| Xanthohumol | Chalcone | 1.87E+04 | 2.20E+04 | 1.67E+04 | 5.07E+03 | 5.35E+03 | 7.15E+03 | 1.09E+00 | 3.06E-01 | -1.71E+00 | up |
| Dihydroquercetin (Taxifolin) | Dihydroflavonol | 9.00E+00 | 1.90E+04 | 9.00E+00 | 9.00E+00 | 9.00E+00 | 9.00E+00 | 1.07E+00 | 1.42E-03 | -9.46E+00 | up |
| Aromadedrin (Dihydrokaempferol) | Dihydroflavonol | 5.81E+05 | 7.86E+05 | 5.53E+05 | 1.79E+05 | 1.59E+05 | 1.64E+05 | 1.15E+00 | 2.61E-01 | -1.94E+00 | up |
| Cyanidin-3,5-O-diglucoside (Cyanin) | Anthocyanin | 1.39E+03 | 1.58E+03 | 9.53E+02 | 5.36E+03 | 6.89E+03 | 7.91E+03 | 1.28E+00 | 5.14E+00 | 2.36E+00 | down |
| 6,8-di-C-glucoside apigenin | Flavone | 5.40E+04 | 1.13E+05 | 9.39E+04 | 2.23E+04 | 1.96E+04 | 1.75E+04 | 1.18E+00 | 2.28E-01 | -2.13E+00 | up |
| Luteolin 7-O-glucoside (Cynaroside) | Flavone | 6.51E+05 | 3.70E+05 | 3.43E+05 | 3.58E+06 | 2.93E+06 | 3.22E+06 | 1.41E+00 | 7.13E+00 | 2.83E+00 | down |
| Chrysoeriol 7-O-hexoside | Flavone | 3.84E+04 | 1.86E+04 | 1.82E+04 | 9.45E+04 | 6.74E+04 | 6.03E+04 | 1.01E+00 | 2.95E+00 | 1.56E+00 | down |
| Luteolin-3',7-di-O-glucoside | Flavone | 4.59E+03 | 2.93E+03 | 3.89E+03 | 2.47E+04 | 2.28E+04 | 2.72E+04 | 1.38E+00 | 6.55E+00 | 2.71E+00 | down |
| Tricetin O-malonylhexoside | Flavone | 1.10E+03 | 1.42E+03 | 1.55E+03 | 9.31E+03 | 8.39E+03 | 7.57E+03 | 1.36E+00 | 6.21E+00 | 2.63E+00 | down |
| Tricetin O-hexoside | Flavone | 5.04E+05 | 1.01E+06 | 4.94E+05 | 3.52E+06 | 3.43E+06 | 3.42E+06 | 1.29E+00 | 5.16E+00 | 2.37E+00 | down |
| Apigenin 7-O-rutinoside | Flavone | 9.05E+03 | 9.91E+03 | 2.23E+03 | 1.52E+05 | 1.37E+05 | 9.53E+04 | 1.72E+00 | 1.81E+01 | 4.18E+00 | down |
| Luteolin 5-O-hexoside | Flavone | 6.35E+04 | 5.56E+04 | 3.76E+04 | 2.16E+05 | 1.79E+05 | 1.94E+05 | 1.15E+00 | 3.76E+00 | 1.91E+00 | down |
| Apigenin 7-O-neohesperidoside (Rhoifolin) | Flavone | 3.00E+03 | 9.06E+02 | 1.41E+03 | 5.11E+04 | 6.75E+04 | 3.69E+04 | 1.86E+00 | 2.93E+01 | 4.87E+00 | down |
| Luteolin | Flavone | 1.17E+04 | 1.26E+04 | 2.04E+04 | 4.45E+03 | 6.14E+03 | 4.13E+03 | 1.02E+00 | 3.29E-01 | -1.60E+00 | up |
| Luteolin O-sinapoylhexoside | Flavone | 1.56E+03 | 2.90E+03 | 1.50E+03 | 6.48E+03 | 6.50E+03 | 5.56E+03 | 1.06E+00 | 3.11E+00 | 1.64E+00 | down |
| Acacetin O-Glucuronic acid | Flavone | 2.32E+03 | 1.44E+03 | 1.55E+03 | 5.68E+03 | 3.73E+03 | 1.06E+04 | 1.06E+00 | 3.77E+00 | 1.91E+00 | down |
| Chrysoeriol O-hexoside | Flavone | 6.22E+04 | 1.37E+05 | 8.60E+04 | 2.50E+05 | 3.05E+05 | 2.61E+05 | 1.01E+00 | 2.86E+00 | 1.52E+00 | down |
| Luteolin O-rutinoside | Flavone | 2.42E+06 | 1.38E+06 | 2.59E+06 | 5.75E+06 | 6.96E+06 | 5.85E+06 | 1.02E+00 | 2.90E+00 | 1.54E+00 | down |
| Isorhamnetin O-acetyl-hexoside | Flavonol | 3.44E+03 | 9.00E+00 | 2.58E+03 | 1.97E+04 | 1.28E+04 | 1.81E+04 | 1.59E+00 | 8.39E+00 | 3.07E+00 | down |
| Astragalin | Flavonol | 4.54E+05 | 5.43E+05 | 4.35E+05 | 1.66E+06 | 1.35E+06 | 1.39E+06 | 1.06E+00 | 3.07E+00 | 1.62E+00 | down |
| Myricetin 3-O-galactoside | Flavonol | 1.37E+05 | 8.09E+04 | 1.23E+05 | 1.11E+06 | 5.68E+05 | 5.07E+05 | 1.33E+00 | 6.41E+00 | 2.68E+00 | down |
| Morin | Flavonol | 1.51E+06 | 8.81E+05 | 1.09E+06 | 6.95E+06 | 4.79E+06 | 6.02E+06 | 1.28E+00 | 5.10E+00 | 2.35E+00 | down |
| Quercetin | Flavonol | 3.09E+05 | 2.28E+05 | 4.44E+05 | 1.37E+06 | 8.41E+05 | 9.67E+05 | 1.06E+00 | 3.24E+00 | 1.70E+00 | down |
| Kaempferitrin | Flavonol | 3.54E+04 | 3.09E+04 | 3.05E+04 | 3.79E+05 | 6.17E+05 | 5.00E+05 | 1.66E+00 | 1.55E+01 | 3.95E+00 | down |
| Quercetin O-hexoside | Flavonol | 3.71E+06 | 7.53E+06 | 4.32E+06 | 2.06E+07 | 2.04E+07 | 2.03E+07 | 1.18E+00 | 3.94E+00 | 1.98E+00 | down |
| Protocatechuic acid | Flavanol | 1.44E+05 | 2.33E+05 | 2.34E+05 | 6.17E+04 | 9.00E+00 | 4.92E+04 | 1.58E+00 | 1.82E-01 | -2.46E+00 | up |
| Protocatechuic aldehyde | Flavanol | 1.55E+06 | 9.00E+00 | 9.00E+00 | 9.00E+00 | 3.87E+06 | 2.95E+06 | 1.29E+00 | 4.40E+00 | 2.14E+00 | down |
| (+)-Gallocatechin (GC) | Flavanol | 1.33E+06 | 1.32E+06 | 8.92E+05 | 6.27E+06 | 7.25E+06 | 3.10E+06 | 1.19E+00 | 4.69E+00 | 2.23E+00 | down |
| Epigallocatechin (EGC) | Flavanol | 9.89E+05 | 6.44E+05 | 5.47E+05 | 5.00E+06 | 3.99E+06 | 2.25E+06 | 1.24E+00 | 5.16E+00 | 2.37E+00 | down |
| L-Epicatechin | Flavanol | 3.83E+06 | 2.56E+06 | 2.08E+06 | 8.61E+06 | 8.98E+06 | 9.58E+06 | 1.08E+00 | 3.21E+00 | 1.68E+00 | down |
| Catechin-catechin-catechin | Flavanol | 1.63E+06 | 8.79E+05 | 5.56E+05 | 1.54E+07 | 1.51E+07 | 1.40E+07 | 1.66E+00 | 1.45E+01 | 3.86E+00 | down |
| Pinobanksin | Flavanone | 5.75E+05 | 9.02E+05 | 1.16E+06 | 4.06E+05 | 1.85E+05 | 2.61E+05 | 1.01E+00 | 3.23E-01 | -1.63E+00 | up |
| Eriocitren | Flavanone | 5.94E+04 | 5.83E+04 | 4.86E+04 | 1.93E+05 | 2.42E+05 | 1.91E+05 | 1.15E+00 | 3.76E+00 | 1.91E+00 | down |
| Butin | Flavanone | 5.61E+04 | 1.00E+05 | 7.93E+04 | 3.65E+05 | 3.11E+05 | 3.28E+05 | 1.21E+00 | 4.27E+00 | 2.09E+00 | down |
| Naringenin 7-O-glucoside | Flavanone | 4.65E+03 | 7.82E+03 | 9.21E+03 | 4.25E+04 | 4.61E+04 | 5.00E+04 | 1.37E+00 | 6.39E+00 | 2.68E+00 | down |
| O-methylnaringenin C-pentoside | Flavanone | 7.54E+03 | 1.01E+04 | 6.45E+03 | 3.56E+04 | 5.11E+04 | 4.00E+04 | 1.29E+00 | 5.26E+00 | 2.39E+00 | down |
| 6-Prenylnaringenin | Flavanone | 3.12E+03 | 7.19E+03 | 3.52E+03 | 1.94E+04 | 2.10E+04 | 1.88E+04 | 1.21E+00 | 4.28E+00 | 2.10E+00 | down |
| Neohesperidin | Flavanone | 1.77E+04 | 2.03E+04 | 2.45E+04 | 8.91E+04 | 8.44E+04 | 5.52E+04 | 1.12E+00 | 3.66E+00 | 1.87E+00 | down |
| Hesperetin 5-O-glucoside | Flavanone | 2.05E+03 | 7.61E+03 | 2.92E+03 | 8.67E+04 | 7.19E+04 | 5.21E+04 | 1.70E+00 | 1.67E+01 | 4.07E+00 | down |
| Prunin | Flavanone | 1.46E+03 | 1.64E+03 | 1.43E+03 | 9.32E+03 | 1.12E+04 | 1.95E+04 | 1.46E+00 | 8.83E+00 | 3.14E+00 | down |
| Naringenin | Flavanone | 2.74E+04 | 3.47E+04 | 3.28E+04 | 4.43E+03 | 6.76E+03 | 4.96E+03 | 1.34E+00 | 1.70E-01 | -2.55E+00 | up |
| 2-Methoxybenzoic acid | Hydroxycinnamoyl derivatives | 9.00E+00 | 9.00E+00 | 9.00E+00 | 2.20E+05 | 1.33E+05 | 9.00E+00 | 2.17E+00 | 1.31E+04 | 1.37E+01 | down |
| Hydrocinnamic acid | Hydroxycinnamoyl derivatives | 9.98E+05 | 5.96E+05 | 8.33E+05 | 9.35E+04 | 1.70E+05 | 2.25E+05 | 1.25E+00 | 2.01E-01 | -2.31E+00 | up |
| Syringin | Hydroxycinnamoyl derivatives | 1.93E+05 | 3.23E+05 | 4.26E+05 | 9.00E+00 | 7.79E+04 | 9.00E+00 | 2.41E+00 | 8.27E-02 | -3.60E+00 | up |
| Caffeic acid O-glucoside | Hydroxycinnamoyl derivatives | 1.87E+06 | 1.53E+06 | 1.65E+06 | 6.02E+05 | 4.98E+05 | 5.81E+05 | 1.05E+00 | 3.33E-01 | -1.59E+00 | up |
| 1-O-beta-D-Glucopyranosyl sinapate | Hydroxycinnamoyl derivatives | 1.24E+06 | 4.04E+06 | 2.02E+06 | 3.37E+05 | 6.78E+05 | 5.60E+05 | 1.14E+00 | 2.16E-01 | -2.21E+00 | up |
| trans-4-Hydroxycinnamic Acid Methyl Ester | Hydroxycinnamoyl derivatives | 2.23E+05 | 1.99E+05 | 1.76E+05 | 6.33E+05 | 4.36E+05 | 8.24E+05 | 1.04E+00 | 3.17E+00 | 1.66E+00 | down |
| Procyanidin B1 | Proanthocyanidins | 7.85E+05 | 8.22E+05 | 7.97E+05 | 1.24E+07 | 1.13E+07 | 1.22E+07 | 1.66E+00 | 1.49E+01 | 3.90E+00 | down |
| procyanidin A | Proanthocyanidins | 6.16E+04 | 5.78E+04 | 6.50E+04 | 1.19E+06 | 1.27E+06 | 1.19E+06 | 1.74E+00 | 1.98E+01 | 4.31E+00 | down |
| Procyanidin B | Proanthocyanidins | 4.83E+05 | 4.71E+05 | 5.16E+05 | 5.68E+06 | 5.83E+06 | 5.81E+06 | 1.58E+00 | 1.18E+01 | 3.56E+00 | down |
