# Supplementary figures and images for: Association of Bitter Metabolites and Flavonoid Synthesis Pathway in Jujube Fruit
Source: Front Nutr. 2022 May 31;9:901756. doi: 10.3389/fnut.2022.901756 (PMC9194943; doi:10.3389/fnut.2022.901756)

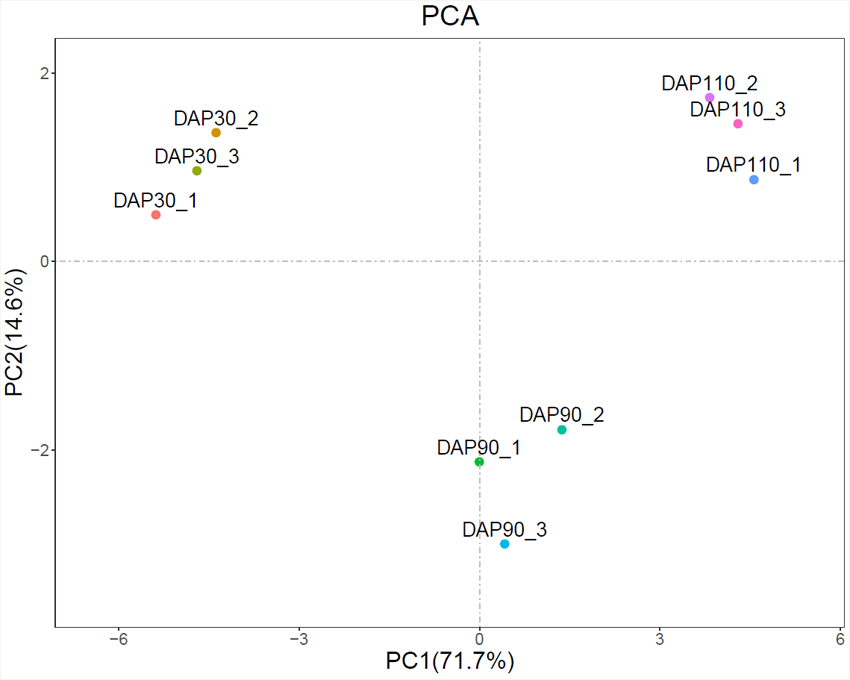

Supplement: Supplementary Figure 1 — Principal component analysis (PCA) of jujube skin metabolites at different development stages. [file Image_1.TIF]

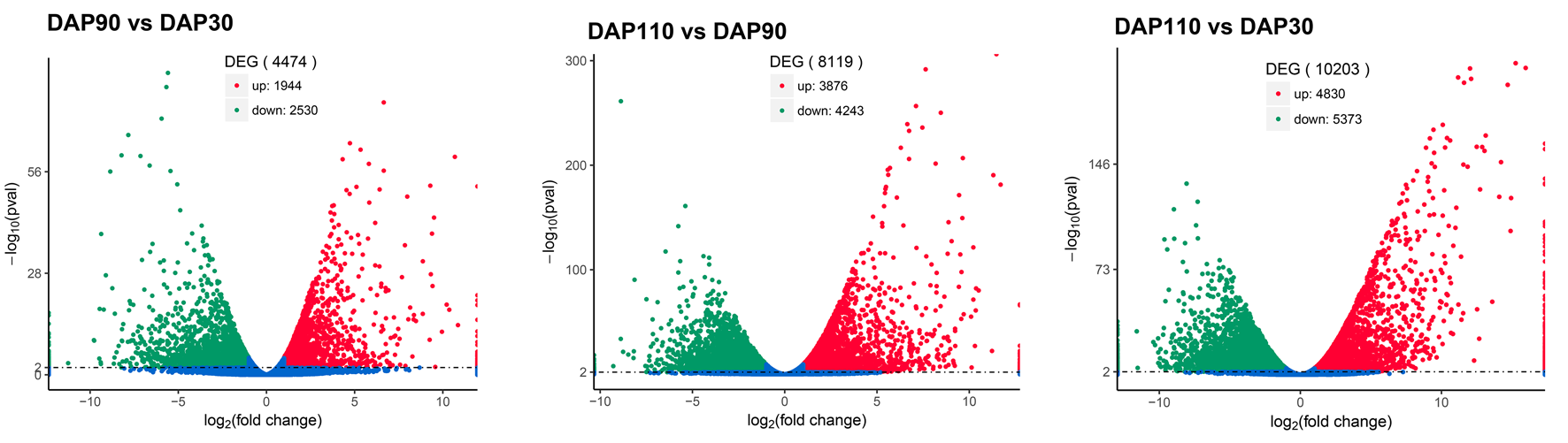

Supplement: Supplementary Figure 2 — Volcano plot of the genes in the fruit skins of the cultivars ‘Junzao’ in this comparison group of DAP90 vs DAP50, DAP110 vs. DAP50 and DAP110 vs DAP90. [file Image_2.TIF]

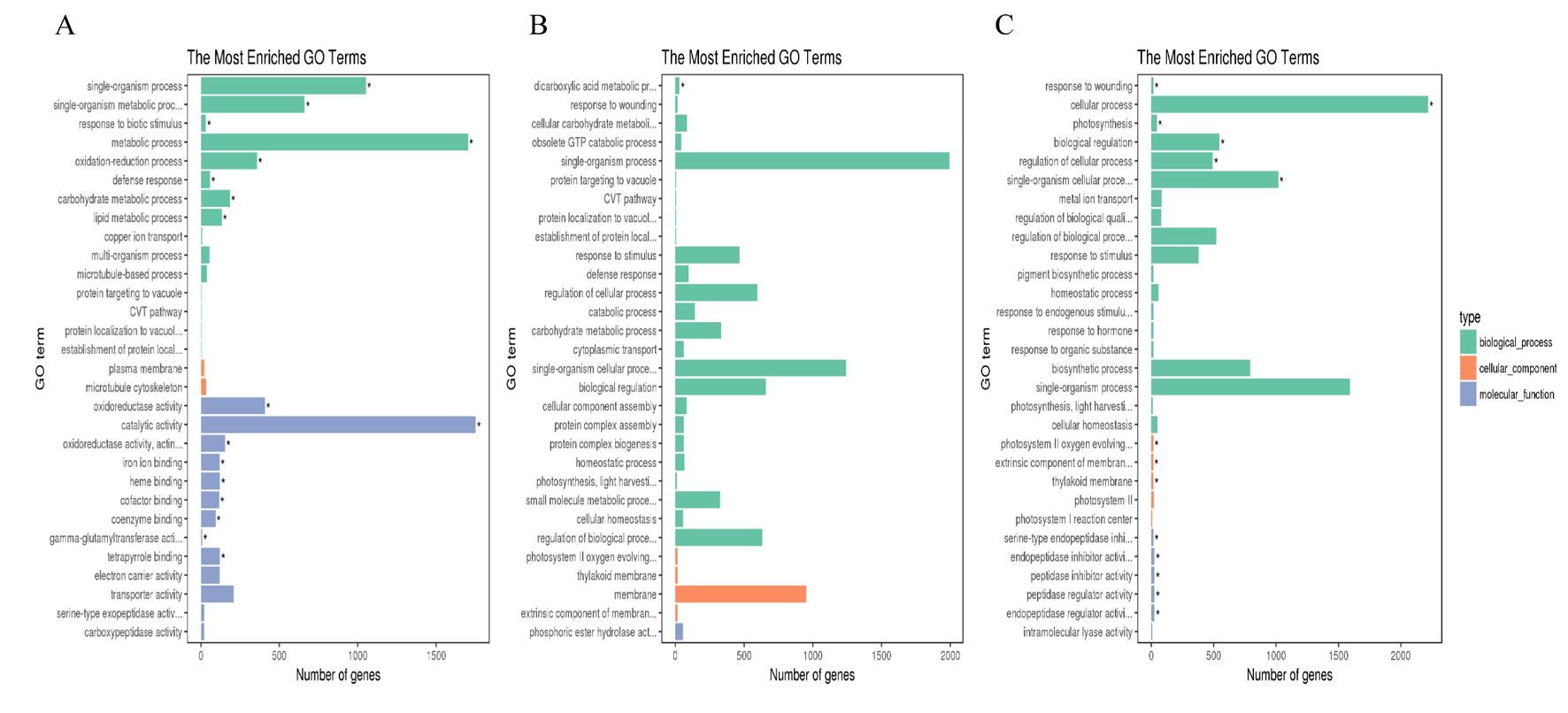

Supplement: Supplementary Figure 3 — Enrichment analysis of GO term in differentially expressed genes. (A) DAP90 vs. DAP50, (B) DAP110 vs. DAP50, (C) DAP110 vs. DAP90. [file Image_3.TIF]

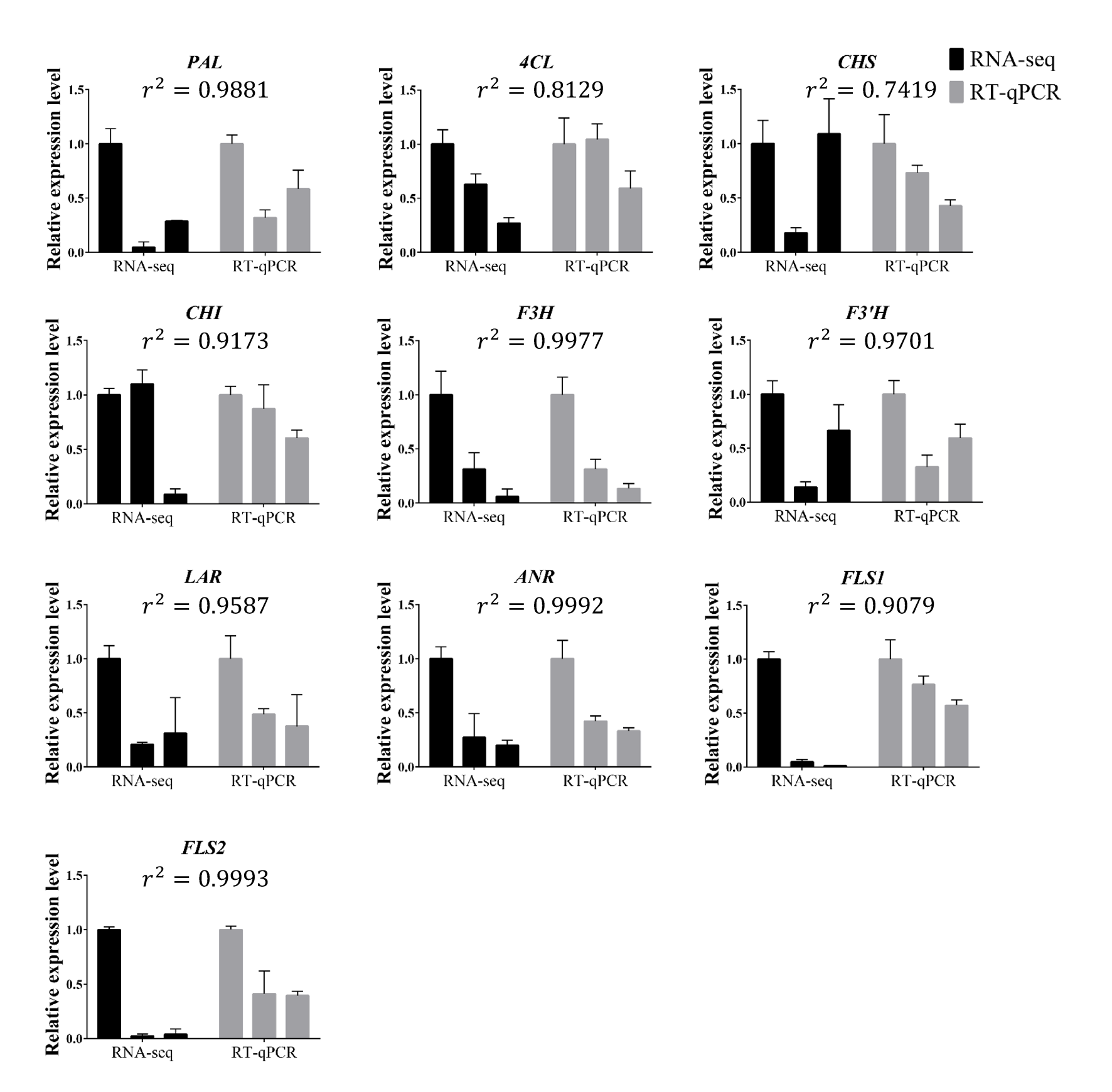

Supplement: Supplementary Figure 4 — Relative expression levels of flavonoid synthesis pathway genes in jujube skins at different developmental stages. The R2 is the square of Pearson’s correlation coefficient. [file Image_4.TIF]
